# Air Pollution Monitoring with Nanoscaled Materials in Chemoresistive Gas Sensors

**DOI:** 10.1021/acsami.5c24099

**Published:** 2026-03-05

**Authors:** Reinaldo S. Theodoro, Gustavo S. M. Santos, Matteo D’Andria, Henrique S. Gropelo, Sebastian Kravecz, Andreas T. Güntner, Diogo P. Volanti

**Affiliations:** † Laboratory of Materials for Sustainability, São Paulo State University, Rua Cristóvão Colombo 2265, 15054-000 São José do Rio Preto, Brazil; ‡ Human-Centered Sensing Laboratory, Department of Mechanical and Process Engineering, 27219 ETH Zurich, CH-8092 Zurich, Switzerland

**Keywords:** toxic gases, nanotechnology, electronic materials, semiconductors, metal oxides, devices

## Abstract

Air pollution is a pressing global concern due to its negative effects on human health and our ecosystem. For instance, pollutants, including particulate matter, volatile organic compounds, nitrogen- and sulfur-based gases, and ozone, are known to increase the incidence rates of respiratory, cardiovascular, and various cancer types, among others. Comprehensive monitoring of key gaseous pollutants is, therefore, critical to enforce adherence to regulatory limits or to control personal exposure. In this review, we analyze the progress on nanostructured and porous chemoresistive gas sensors over the last five years and critically compare their performance to air pollution guidelines. We start with a discussion of the major outdoor and indoor pollutants, describing their main sources and the associated health effects arising from short- and long-term exposures to concentrations exceeding national and regional limits. Thereafter, we describe the working mechanism of chemoresistive gas sensors along with their key performance parameters, followed by a literature survey of several nanoscaled porous materials for such applications. We highlight different engineering strategies focused on structural, morphological, and electronic control through heterostructures, surface functionalization, and metal–organic framework templates that tune air pollutant adsorption, catalytic conversion on their surfaces, and sensor signal generation. Finally, we briefly discuss the integration of these gas-sensing technologies into functional devices to translate material and surface innovation into environmental monitoring platforms for consumer electronics, wearables, and smart-home solutions.

## Graphical abstract

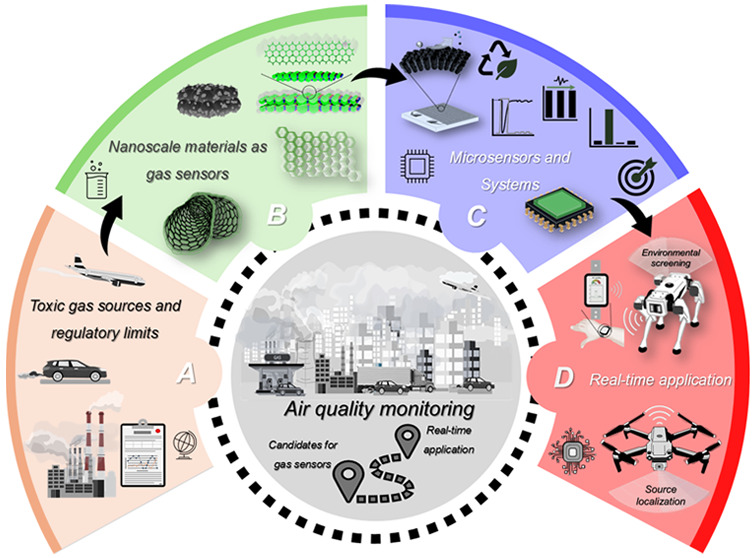

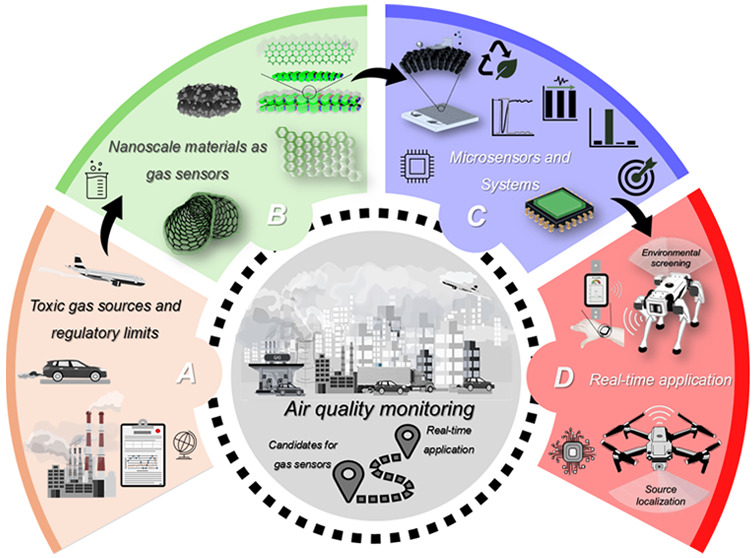

## 1 Introduction

Air pollution is a major social and environmental concern, causing severe impacts on both human health and the environment. Data published by the World Health Organization (WHO) on air quality standards indicate that 99% of the global population is routinely exposed to indoor and outdoor environments with pollution levels exceeding the recommended air quality limits.^1^ This is particularly critical in urban settings due to intense transportation and concentrated industrial activities.^2^ In 2019, air pollution was associated with approximately 6.7 million premature deaths worldwide,^3^ with a higher prevalence in developing countries, particularly in the Southeast Asian and Western Pacific regions.^4^

Groups strongly susceptible to the adverse effects of air pollution include children and the elderly, middle-aged adults,^5^ pregnant women,^6^ and individuals with pre-existing health conditions, such as asthma,^7^ hypertension, respiratory and cardiovascular diseases.^8^ Furthermore, studies have shown that older women and African Americans living in urban areas of the United States are more vulnerable to respiratory problems associated with air pollution caused by wildfires and the generation of particulate matter (PM).^9^ Further studies indicate that socioeconomic factors play a role; thus, low-income populations tend to be at higher risk.^10^

The main air pollutants include CO, O_3_, NO*_x_*, SO*_x_*, Pb, Cd, and fine particulate matter (PM_2.5_ and PM_10_),^4^ volatile organic compounds (VOCs), and pathogens (fungi, bacteria, and viruses).^11^ Long- and short-term exposure to high levels of air pollutants has been linked to an increased risk of respiratory and cardiovascular diseases,^12^ headaches, dizziness, irritation of the mucous membranes, skin, eyes, and allergic reactions.^13^ Furthermore, several compounds have toxic effects on the body, including neurotoxicity, neurodegenerative conditions, and carcinogenicity.^14^ Among the carcinogenic^15^ compounds are benzene, formaldehyde, acetaldehyde, naphthalene, ethylbenzene, tetrachloroethylene, and styrene that have been associated with the development of lung, breast, and thyroid cancer as well as leukemia.^16,17^ Air pollutants are also responsible for climate change and damage to ecosystems. Acid rain, particulate matter deposits^18^ and other air pollutants interfere with plant metabolism and physiology,^19^ promoting acidification and altered nutrient cycling in soils^20^ that results in economic loss.

The development and application of innovative sensor materials and devices for monitoring and mitigating gaseous pollutants are of fundamental importance. However, most available air quality sensors lack the required performance to track critical pollutants.^21^ Figure 1 illustrates the key performance and economic metrics that air quality sensors need to meet. Detection challenges involve: (1) achieving a satisfactory lower limit of detection (LLOD) to recognize air pollutants at their typical trace concentrations, (2) selectivity to discriminate between different chemical species, (3) fast and reversible response needed for continuous monitoring,^22,23^ (4) operational stability and (5) robustness against environmental conditions, including temperature and relative humidity fluctuations.^24,25^ Furthermore, economic considerations encompass the scalable manufacturing and use of sustainable, low-cost materials with minimal energy consumption.

**Figure 1:**
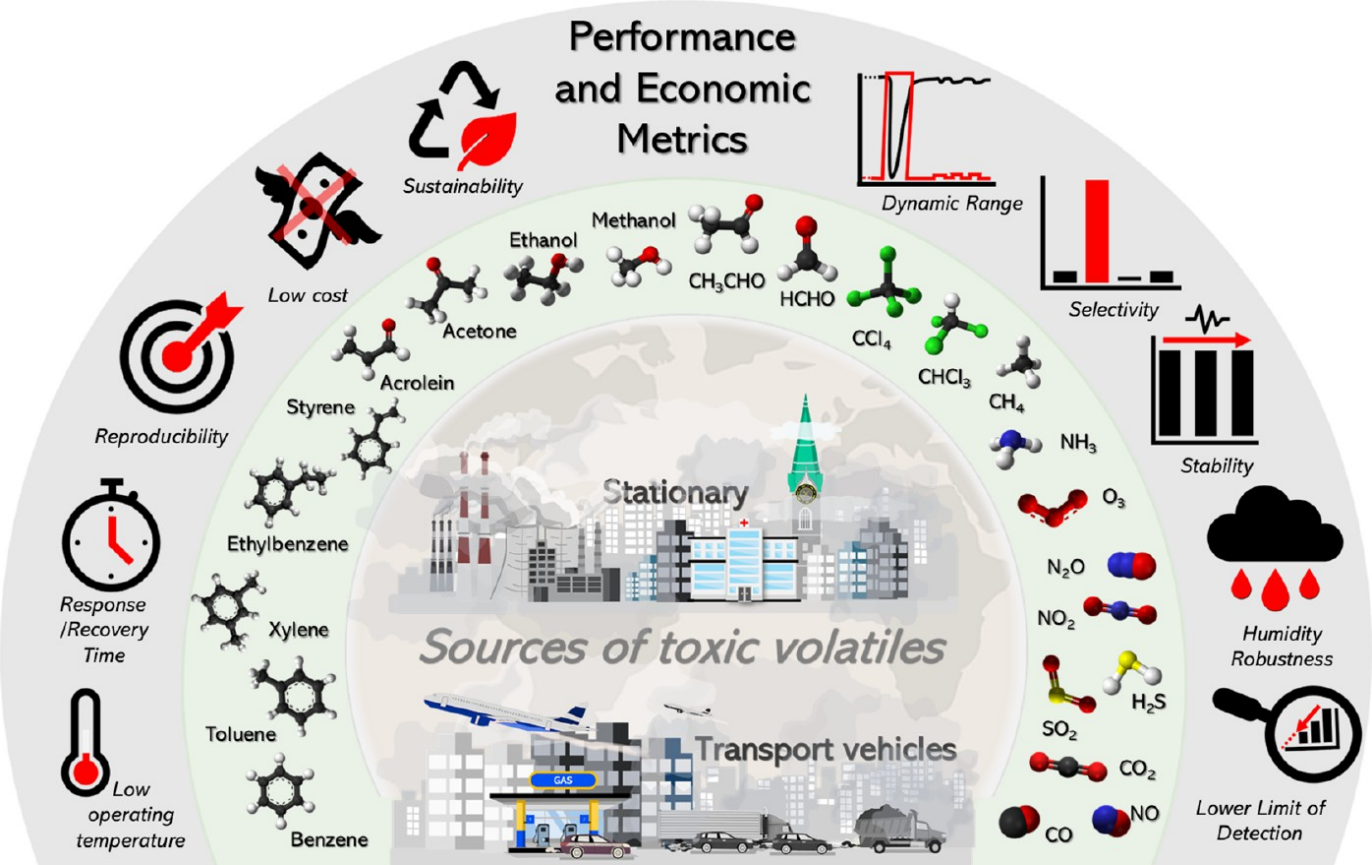
Schematic illustration of the main air pollutants, environmental sources, performance metrics, and economic aspects of chemoresistive sensors.

In solid-state chemoresistive sensors, surface interactions with gaseous molecules cause variations in the electrical conductivity of the material,^26,27^ enabling the detection of chemical species.^28^ In this context, the response to air pollutants depends entirely on the interaction between these molecules and the active surface of the sensor, resulting in signal transduction at the interface. Promising strategies to design high-performance gas sensors rely on the deployment of nanometric porous materials (1–100 nm),^29–31^ semiconductor metal oxides,^32–39^ nitrides,^40,41^ sulfides,^42,43^ bromides,^44,45^ metal–organic frameworks (MOFs),^46–51^ zeolites,^52,53^ functionalized materials based on carbon nanotubes,^54^ graphene,^55,56^ noble metals, and composites.^57,58^ The nature of the physical and chemical interactions between the VOCs and the sensor surface affects the detection mechanism.^59^ Further, structural factors such as nanoparticle morphology, film architecture, specific surface area, density of active sites, grain size, and porosity influence the electrical gas responses of semiconductors.^60,61^ Altogether, these highlight the need for a thorough analysis of the intertwined physicochemical and mesoscale features that ultimately govern gas–solid interactions and charge-transport pathways.

Previous reviews have explored the development of chemoresistive gas sensor materials with high porosity, surface area,^62–64^ activated adsorption sites, gas diffusion ability,^65,66^ catalytic properties,^67^ and material defects—oxygen and metal vacancies^68–71^ —very often responsible for improving sensing performance of many material types. These reviews covered diverse synthesis technologies, such as hydrothermal and solvothermal (including microwave-assisted),^72^ coprecipitation,^73^ chemical reduction,^74^ sol–gel,^75^ flame spray pyrolysis (FSP),^76^ electrospinning,^77^ and others to obtain different chemoresistive gas sensor materials. Also, operational concepts (e.g., light activation)^78^ and signal processing strategies^79^ have been covered. A wide range of applications is targeted, among health/medical,^80–88^ space^89^ and air quality,^90^ with different approaches to environmental monitoring based, for instance, on emerging “Internet of Things” (IoT) and Artificial Intelligence (AI) capabilities.^91–94^ Yet, none of them has offered a detailed comparison of sensor performance against current exposure guidelines from agencies such as the EPA (U.S.), EEA (Europe), and MEE (China).^95–98^

This review provides a comprehensive overview of state-of-the-art chemoresistive sensor technologies developed over the past five years. We assess recent innovations based on their adherence to national and regional air quality exposure guidelines to benchmark progress and identify opportunities for further research and development. We begin by summarizing the main toxic volatile compounds, their emission sources, the recommended exposure limits for each analyte, and the associated health effects of prolonged exposure (Section 2). Based on these regulatory requirements, we identify chemoresistive gas sensors (published within the last five years) that achieve the required performance metrics (Section 3, Table 2). Additionally, we elaborate on the fundamental sensing mechanisms of chemoresistive materials, such as metal oxides (MO*_x_*), transition metal dichalcogenides (TMDs), transition metal nitrides (TMNs), transition metal halides (TMHs), MXene composites, metal–organic frameworks (MOFs) and their derivatives, conductive polymers, and organic materials, highlighting the key physicochemical properties and interactions that govern their sensing performance. From this analysis, we identify strategies to overcome existing limitations in sensing performance and in the practical implementation of these materials into functional devices (Sections 4). To summarize, Figure 2 shows a systematic overview of the transition from nanoparticles and porous materials fabrication to real functional devices, emphasizing the need for engineering across multiple length scales to preserve the sensing performance while ensuring reliable operation in real-time air quality monitoring under real conditions.

**Figure 2:**
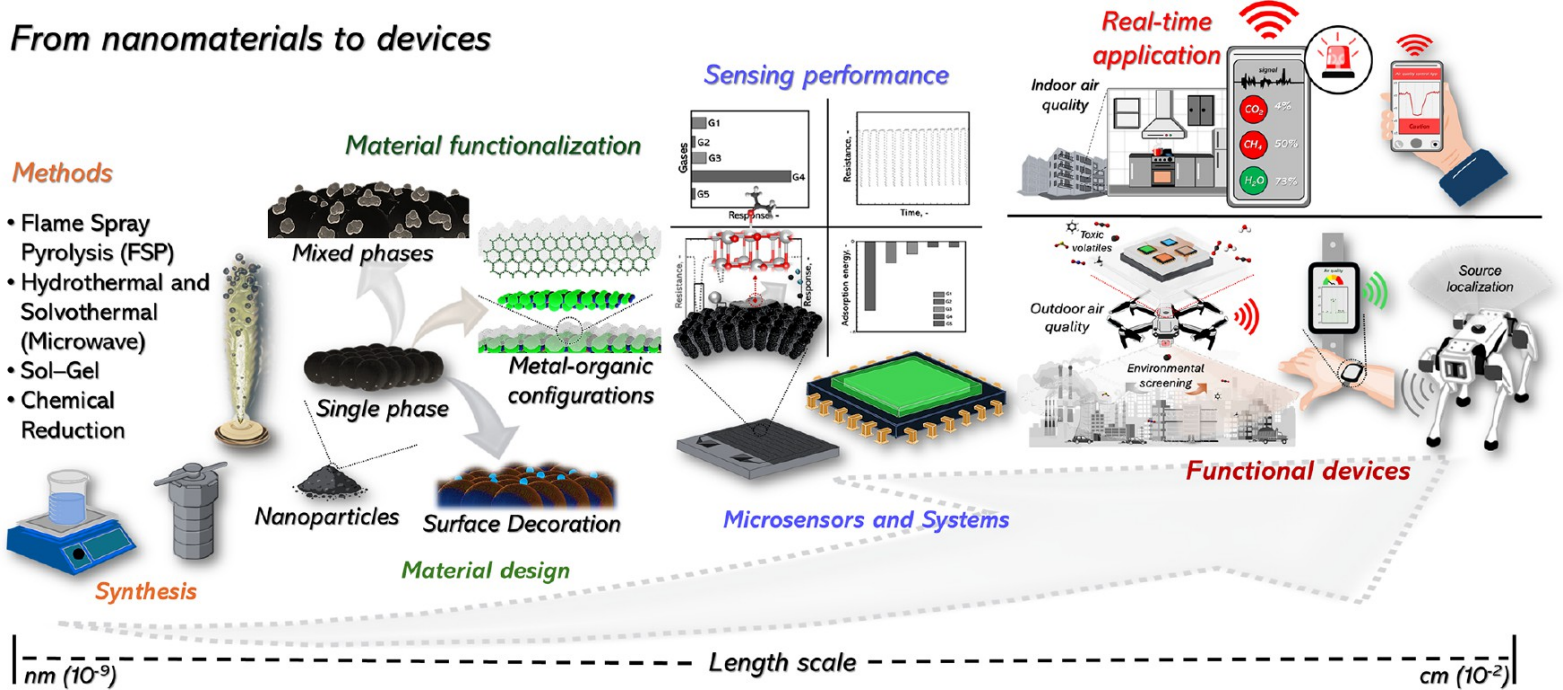
Schematic overview illustrating the pathway from nanoscale material synthesis (nanoparticles and porous materials) to sensor functionalization, performance evaluation, microsensor integration, and real-time air quality monitoring applications.

## 2 Air Pollutants: Origin, Health Effects, and Exposure Limits

### 2.1 Classes of Gaseous Pollutants

Air pollutants are classified as primary and secondary, according to their origin. Primary pollutants, such as SO_2_, NO*_x_*, CO, CO_2_, CH_4_, NH_3_, VOCs, and particulate matter, are emitted directly into the atmosphere, primarily by industrial activities, fossil fuel combustion, mobile source emissions, agricultural and deforestation activities, as well as petroleum and mining processes.^99^ In turn, secondary pollutants, such as ozone, nitrites, nitrates, peroxyacetyl nitrate, sulfates, and aldehydes, result from chemical reactions between primary pollutants and the atmosphere,^100^ intensifying the greenhouse effect and contributing to photochemical smog.^101,102^

Although there is extensive discussion of environmental pollution guidelines focused on particulate matter and inorganic compounds, little emphasis is given to the importance and impact of VOCs.^103^ These encompass a diverse class of hydrocarbon-based organic chemical compounds that have relatively high vapor pressure and low molecular weight,^15^ as well as resistance to spontaneous degradation.^104,105^ Furthermore, VOCs can be classified according to their volatility and intensity of emission into the environment, highlighting: *highly volatile* compounds, such as methyl chloride, propane, and butane; *volatile* compounds, such as alcohols, ketones, formaldehyde, and aromatic organic compounds (benzene, toluene, xylene, etc.); and *semivolatile* compounds, such as pesticides, organophosphates and PAHs.^101,106^

Naturally occurring VOCs result from atmospheric emissions, forest fires, volcanic and geothermal activity, microbiological processes, dust, and aerosols. On the other hand, the main toxic VOCs are associated with emissions from anthropogenic sources, stationary sources, transport vehicles, and intense human activity, as represented in Figure 1.^101^ Studies indicate that many synthetic materials and human activities increase the release of toxic volatile compounds into indoor environments, resulting in VOC concentrations that are often higher than those observed outdoors.^107,108^ This difference stems from the diverse sources of VOCs, such as acetone, ethanol, benzene, toluene, xylene, formaldehyde, and ethylbenzene, present in indoor environments, with approximately 52% derived from building materials alone.^15^ Furthermore, the use of cleaning, hygiene, and cosmetic chemicals, furniture and decorative materials, paints, heaters, cooking gas, and smoking primarily contribute to this effect.^104,109^

This section reviews the main types of air pollutants, emphasizing their main emission sources, adverse effects on human health and in animal models, as available in the literature, and the exposure limits established by different regulatory bodies. The information presented in Table 1 was compiled from databases provided by internationally recognized institutions such as the World Health Organization (WHO), the United States Environmental Protection Agency (US EPA), the National Institute for Occupational Safety and Health (NIOSH), the Centers for Disease Control and Prevention (CDC), the National Institutes of Health (NIH), the Occupational Safety and Health Administration (OSHA), the American Conference of Governmental Industrial Hygienists (ACGIH), the International Agency for Research on Cancer (IARC), and the American Lung Association (ALA). Furthermore, comparisons were made with the European Environment Agency (EEA), European Chemicals Agency (ECHA), Health and Safety Executive (HSE), Institute for Occupational Safety and Health of German Social Accident Insurance (IFA), Chinese Ministry of Ecology and Environment (MEE), and Chinese National Health Commission (NHC), as specified by the respective citations.

**Table 1:** Summary of Key Gaseous Pollutants with Toxic Effects and Typical Concentration Ranges^a^

		Exposure guidelines		
Classes of gaseous pollutants		U.S.A. and Canada	European Union	China
Alcohols	Methanol	0.5 mg/kg/d or 10.0 mg/m^3^ (RfD based on increased liver enzymes and decreased brain weight in rats). The odor perception threshold is 2.0 ppm (2.62 mg/m^3^) (EPA).^110^	260 mg/m^3^ (200 ppm) long-term exposure (ECHA/OELs).^111^	50 mg/m^3^ short-term exposure and 25 mg/m^3^ - 8 h (MEE, NHC).^112^
	Ethanol	1900.0 mg/m^3^ (1000 ppm) long-term exposure (10 h) (NIOSH, OSHA, CDC).^113,114^	1907 mg/m^3^ (1000 ppm) (EU-IOEL).^115^	–
Ketones	Acetone	594.0 mg/m^3^ (250 ppm) long-term exposure (10 h) (ACGIH).^116^	1210.0 mg/m^3^ (500 ppm)—8 h (ECHA).^117^	450 mg/m^3^ short-term exposure and 300 mg/m^3^—8 h (MEE, NHC).^112^
Aldehydes	Formaldehyde	0.12 mg/m^3^ (0.1 ppm) long-term exposure and 0.004 mg/m^3^ (0.003 ppm) based on respiratory effects (ACGIH).^118^ The odor perception threshold is 0.83 ppm (EPA).^119^	0.37 mg/m^3^ limit value. (IFA, AGW).^120^ 0.37 mg/m^3^ (0.3 ppm) long-term exposure and 0.74 mg/m^3^ (0.6 ppm) short-term exposure (dermal sensitization). (ECHA).^121^	0.5 mg/m^3^ maximum allowable concentration (MAC).
				≤0.08 mg/m^3^ average hour indoor (MEE, NHC).^112,122^
	Acetaldehyde	45 mg/m^3^ (25 ppm) recommended limit.	25 ppm short-term exposure.	0.3 mg/m^3^ maximum allowable concentration (MAC) (MEE).^112^
		The odor perception threshold is 0.05 ppm (0.09 mg/m^3^) (EPA, ACGIH).^123,124^	91 mg/m^3^ (50 ppm) (MAK, WHO)^125^	
	Acrolein	Recommended limit 0.1 ppm.	0.05 mg/m^3^ (0.02 ppm) long-term exposure and 0.12 mg/m^3^ (0.05 ppm) short-term exposure (ECHA/OELs).^126^	0.3 mg/m^3^ maximum allowable concentration (MAC) (MEE).^112^
		0.003 ppm (0.007 mg/m^3^, MRL - based on respiratory effects in humans). 0.0005 mg/Kg/d, (RfD - based on reduced survival after oral exposures in an animal study).		
		The odor perception threshold is 0.25 ppm (0.60 mg/m^3^) (NIOSH, EPA, OSHA).^127,128^		
Aromatic hydrocarbons	Benzene	0.02 ppm long-term exposure (ACGIH).^129^	0.66 mg/m^3^ (0.2 ppm) limit value (ECHA).^130^	10 mg/m^3^ short-term exposure and 6 mg/m^3^ - 8 h. ≤0.03 mg/m^3^ average hour indoor (NHC, MEE).^112,122^
		0.03 mg/m^3^—RfC and 0.004 mg/Kg/d—RfD based on hematologic effects in humans. The odor perception threshold is 1.5 ppm (5.0 mg/m^3^) (EPA).^131^	Acceptable concentration: 0.2 mg/m^3^. Tolerable concentration: 1.9 mg/m^3^ (IFA, TRGS 910).^130,132^	
			5 μg/m^3^ - 1 year (EC).^133^	
	Toluene	20 ppm long-term exposure recommended limit (ACGIH).^134^	192 mg/m^3^ (50 ppm) - Long-term exposure and 384 mg/m^3^ (100 ppm) - Short-term exposure (through skin absorption) (ECHA/OELs).^135^	100 mg/m^3^ short-term exposure and 50 mg/m^3^ - 8 h. ≤0.20 mg/m^3^ average hour indoor (NHC, MEE).^112,122^
		5.0 mg/m^3^ (RfC - based on neurological effects in humans). 0.08 mg/Kg/d (RfD - based on increased kidney weight in rats). The odor perception threshold is 2.9 ppm (EPA).^136^		
	Xylenes	Recommended limit 100 ppm (ACGIH).^137^ 0.4 mg/m^3^ (0.1 ppm MRL, based on neurological effects in occupationally exposed Workers); The odor perception threshold for m-xylenes is 1.1 ppm (EPA).^138^	o,p,m-Xylene: 221 mg/m^3^ (50 ppm) - 8 h and 442 mg/m^3^ (100 ppm) - Short-term exposure (through skin absorption) (IOELVs).^139^	100 mg/m^3^ short-term exposure and 50 mg/m^3^ - 8 h. ≤0.20 mg/m^3^ average hour indoor (NHC, MEE).^112,122^
	Styrene	10 ppm long-term exposure recommended (ACGIH).^140^	20 ppm (8 h) long-term inhalation exposure and 68 ppm short-term inhalation exposure. The odor perception threshold is 0.1 ppm (0.43 mg/m^3^) (Plastics Europe, 2024).^141^	100 mg/m^3^ short-term exposure and 50 mg/m^3^ - 8 h (NHC, MEE).^112^
		1.0 mg/m^3^ (DfC -based on CNS effects in occupationally exposed workers). The odor perception threshold is 0.32 ppm (EPA).^142^		
	Ethylbenzene	1.0 mg/m^3^ (DfC - based on the development in rats and rabbits); 0.1 mg/Kg/d (DfR - based on hepatic and renal toxicity in rats); The odor perception threshold is 2.3 ppm (EPA).^143^	442 mg/m^3^ (100 ppm) - 8 h and 844 mg/m^3^ (200 ppm) short-term exposure (through skin absorption) (HSE).^144^	150 mg/m^3^ short-term exposure and 100 mg/m^3^ - 8 h (NHC, MEE).^112^
Sulfur compounds	H_2_S	7 mg/m^3^ (5 ppm) short-term exposure limit and 1.4 mg/m^3^ (1 ppm) long-term exposure (OSHA, ACGIH).^145^	7.0 mg/m^3^ (5 ppm) long-term exposure and 14.0 mg/m^3^ (10 ppm) short-term exposure (ECHA/OELs).^146^	10 mg/m^3^ maximum allowable concentration (MAC) (NHC, MEE).^112^
Organochlorines	Chloroform	0.2 mg/m^3^ or 0.05 ppm for intermediate inhalation (ATSDR - based on worker exposures resulting in liver effects in humans); 0.1 mg/m^3^ or 0.02 ppm for chronic inhalation (MRL - based on liver effects in humans). The odor perception threshold is 85.0 ppm (EPA).^147^	10.0 mg/m^3^ (2 ppm) long-term exposure (8 h) (through skin absorption) (ECHA, IOELVs).^117,148^	20 mg/m^3^ - 8 h (NHC, MEE).^112^
	Carbon tetrachloride	31 mg/m^3^ (ppm) long-term exposure (ACGIH).^149^	6.4 mg/m^3^ (1 ppm) long-term exposure and 32.0 mg/m^3^ (5 ppm) short-term exposure (through skin absorption) (ECHA/OELs).^150^	25 mg/m^3^ short-term exposure and 15 mg/m^3^ - 8 h. (NHC, MEE).^112^
		1.3 mg/m^3^ or 0.2 ppm (ATSDR - based on liver effects in rats, inhalation acute); The odor perception threshold is 10.0 ppm (EPA).^151^		
Other gases	CH_4_	EEL of 5000 ppm (24 h) and CEL for 5000 ppm (90 days) (NIH).^152^	No single occupational exposure limit.	No single occupational exposure limit (Standard GBZ 2.1–2019).
	CO	4 mg/m^3^ (24 h) exposure limits. Recommended limit 35 ppm (NIOSH, WHO, IARC, ACGIH).^153–155^	23 mg/m^3^ (20 ppm) long-term exposure and 117.0 mg/m^3^ (100 ppm) short-term exposure (through skin) (ECHA, IFA, AGW). 0.5 μg/m^3^ - 8 h (EC).^120,133,156^	10 mg/m^3^ - 1 h or 4 mg/m^3^ - 24 h (NHC, MEE).^112,157^
	CO_2_	9000.0 mg/m^3^ (5000.0 ppm) (NIOSH).^158^	9000.0 mg/m^3^ (5000.0 ppm) long-term exposure (ECHA).^159^	18,000 mg/m^3^ short-term exposure and 9000 mg/m^3^ - 8 h. ≤0.10 mg/m^3^ average hour indoor (NHC, MEE).^112,122^
	NO_2_	25 μg/m^3^ (24 h) and 10 μg/m^3^ annually. 0.38 mg/m^3^ (0.2 ppm) - 8 h long-term exposure (WHO, IARC ACGIH).^153,154,160^	0.96 mg/m^3^ (0.5 ppm) long-term exposure and 1.91 mg/m^3^ (1.0 ppm) short-term exposure (ECHA).^161^	10 mg/m^3^ short-term exposure and 5 mg/m^3^ - 8 h. 80 μg/m^3^ annual or 200 μg/m^3^ (24 h) (NHC, MEE).^112,157^
			200 μg/m^3^ - 1 h or 40 μg/m^3^ - 1 year (EC).^133^	
	NH_3_	18.0 mg/m^3^ (25 ppm) long-term limit (10 h) (OSHA).^162^	14.0 mg/m^3^ (20 ppm) - 8 h or 36 mg/m^3^ (50 ppm) short-term exposure (IOELVs).^163^	30 mg/m^3^ short-term exposure and 20 mg/m^3^ - 8 h. ≤0.20 mg/m^3^ average hour indoor (NHC, MEE).^112,122^
	O_3_	0.1 ppm. 100 μg/m^3^ (8 h) exposure limit (ACGIH).^164^	Daily maximum of 120 μg/m^3^ - 8 h (EC).^133^	200 μg/m^3^ - 1 h or 160 μg/m^3^ - 8 h (MEE).^157^
	SO_2_	40 μg/m^3^ (2.0 ppm) - 24 h (ACGIH).^165^	350 μg/m^3^ - 1 h or 125 μg/m^3^ - 24 h (EC).^133^	10 mg/m^3^ short-term exposure and 5 mg/m^3^ - 8 h. 150 μg/m^3^ annual or 500 μg/m^3^ (24 h) (MEE).^157^

a Abbreviations: RfC—inhalation reference concentration; RfD—reference dose; MRL—acute minimal reference level.

#### 2.1.1 Alcohols

Methanol and ethanol are primarily present in distillation factories, motor fuels, pharmaceuticals, and pigments production.^166^ They are considered moderately toxic yet teratogenic compounds. Exposure can cause headaches, dizziness, and allergic reactions. Methanol is a widely used chemical feedstock in laboratories and chemical plants, posing a potential hazard for intoxication. Its ingestion, inhalation, or skin absorption leads to irreversible tissue damage to the eyes and nervous system, or even death.^167^ Especially in developing countries, methanol poisoning outbreaks occur frequently due to adulterated alcohol,^168^ such as in India with >90 deaths in February 2019.^169^ In 2025, a serious incident of methanol poisoning occurred in Brazil linked to the commercialization of adulterated alcoholic beverages. The outbreak resulted in severe symptoms in 209 suspected cases and 15 confirmed deaths as of the time of this discussion.^170^ Such events highlight the ongoing risks associated with illicit alcohol production and underscore the need for stricter regulatory monitoring and public awareness to prevent future occurrences.

#### 2.1.2 Ketones

Acetone is widely used as a solvent in various applications including chemical cleaning products, hygiene products, and paints. Long-term exposure can irritate mucous membranes and damage the respiratory and nervous systems. The National Institute for Occupational Safety and Health recommends a long-term (10 h) exposure of 250 ppm–594.0 mg/m^3^.^171^

#### 2.1.3 Aldehydes

Formaldehyde (CH_2_O) is a colorless and flammable gas produced by the oxidation of methanol or methane in the presence of a catalyst.^172^ Formaldehyde is a major indoor pollutant in various industries, including the production of cosmetics, paints, formaldehyde-based wooden products, and during the combustion of biofuels.^172–174^ However, formaldehyde is also a concern in outdoor environments due to the increase in forest fires and the large-scale consumption of biofuels in recent years. Exposure to formaldehyde can lead to various health issues in humans. Formaldehyde acute effects mainly occur by inhalation and can cause coughing, chest pain, irritation in different parts of the body, and wheezing. Other chronic disorders, such as respiratory infections, dermatitis, and skin irritations, can be observed after long-term exposure to formaldehyde.^119^ Acetaldehyde (CH_3_CHO) is a colorless gas, flammable, and exhibits a fruity odor at lower concentrations.^175^ The main sources of acetaldehyde are summarized in the production of resins, perfumes, and are used as a solvent in the rubber and paper industries.^176^ Acetaldehyde also plays an important role in the metabolism of plants and animals.^177^ Irritation of the eyes, skin, and respiratory tract is one of the acute effects caused by. Acetaldehyde is classified as a possible human carcinogen (Group B2) by the EPA.^178^

#### 2.1.4 Aromatic Compounds

Aromatic compounds, such as benzene, toluene, and xylene, exhibit a high degree of toxicity and carcinogenicity upon exposure. Benzene, for instance, is primarily released during gasoline handling at gas stations and is also found in building materials, tobacco smoke, and furniture.^179,180^ Loss of consciousness, headache, confusion, and drowsiness are some of the acute effects caused by exposure to benzene.^181^ Benzene is classified as a Group 1 carcinogen in humans by the International Agency for Research on Cancer (IARC) during chronic exposure. Other chronic effects may be observed in humans, such as lung cancer and leukemia.^182^ Toluene and xylenes are primarily found in the automotive industry, cigarette smoke, gas stations, refineries, and as solvents in adhesives and cleaning agents.^183,184^ These aromatic compounds also present high toxicity and carcinogenic levels. For instance, exposure to toluene may affect the central nervous system, causing headaches, fatigue, drowsiness, and cardiac arrhythmia.^185^ Upon exposure to xylene, similar effects can be observed in humans, including neurological issues, impacts on lung function, gastrointestinal function, and dyspnea.^186^

#### 2.1.5 Sulfur Compounds

Hydrogen sulfide (H_2_S) is a flammable and colorless gas characterized by the distinctive odor of rotten eggs. H_2_S is a gas of considerable environmental and occupational concern. It is generated primarily during processes such as oil and natural gas refining, the decomposition of human and animal waste, the treatment of industrial effluents, and the manufacturing of fertilizers and various chemicals.^187,188^ In addition, large amounts of H_2_S are naturally released in landfills as a byproduct of the anaerobic breakdown of organic matter.^189^ Due to its physicochemical properties, H_2_S is highly toxic and can be absorbed rapidly through the lungs upon inhalation. Acute exposure may result in severe irritation of the respiratory tract, often accompanied by neurological symptoms such as seizures, headaches, dizziness, and even apnea. At higher concentrations, the gas can impair cellular respiration by inhibiting cytochrome oxidase, leading to systemic toxicity and, in extreme cases, fatal outcomes.^190^ Exposure to H_2_S is a significant concern in public health, particularly due to the large number of industrial facilities that release this pollutant gas.

#### 2.1.6 Organochlorines

This class includes chloroform and carbon tetrachloride. Chloroform is derived from sources such as hydrochlorofluorocarbons, solvent use, water chlorination processes, pulp and paper mills, and landfills. Carbon tetrachloride is used in the manufacture of refrigerants, aerosols, solvents, rubbers, and paints. Among the acute effects, depression of the central nervous system, liver, and kidneys stands out, but exposure can also cause pulmonary edema.

#### 2.1.7 NO*_x_*, CO_x_, NH_3_, SO*_x_*, and O_3_

These pollutants comprise a group of gases released into the atmosphere on a large scale, primarily through human activities that intensify the greenhouse effect and contribute to the formation of photochemical smog. CO_2_ and carbon monoxide CO are primarily produced by the burning of fuels and wood, which is directly related to financial interests at the expense of sustainability. Other gases, such as NO*_x_* and SO*_x_*, are also released by combustion engines and coal-fired power plants. These compounds and VOCs can form secondary pollutants, including O_3_, through photochemical reactions when exposed to sunlight. In general, exposure to these highly toxic gases can cause pulmonary and cardiovascular diseases, allergic reactions, skin and mucous membrane irritation, difficult breathing, coughing, dizziness, and pulmonary edema, so prolonged exposure at high concentrations can be fatal.^153^

## 3 State-of-the-Art Gas Sensors for Air Pollutant Detection

Chemiresistive gas sensors are based on various classes of materials (Figure 3) and can be broadly divided into two groups: inorganic and carbon-based materials. The inorganic materials include semiconducting metal oxides (SMO*_x_*),^66,191^ transition metal dichalcogenides (TMDs),^192,193^ transition metal nitrides (TMNs),^194^ transition metal halides (TMHs, e.g., bromide compounds),^195^ and MXene-based materials^196^ (Figure 3, orange-shaded). On the other hand, the carbon-based materials comprise porous materials such as metal–organic frameworks (MOFs) and covalent–organic frameworks (COFs),^197–199^ graphene and reduced graphene oxide (rGO),^200^ carbon nanotubes (CNTs),^201^ conducting polymers, and other organic sensing materials^202,203^ (green-shaded). These materials can be combined to yield new material compositions and interfaces capable of tuning sensitivity, selectivity, stability, response and recovery times, and lower limit of detection (LLOD).

**Figure 3:**
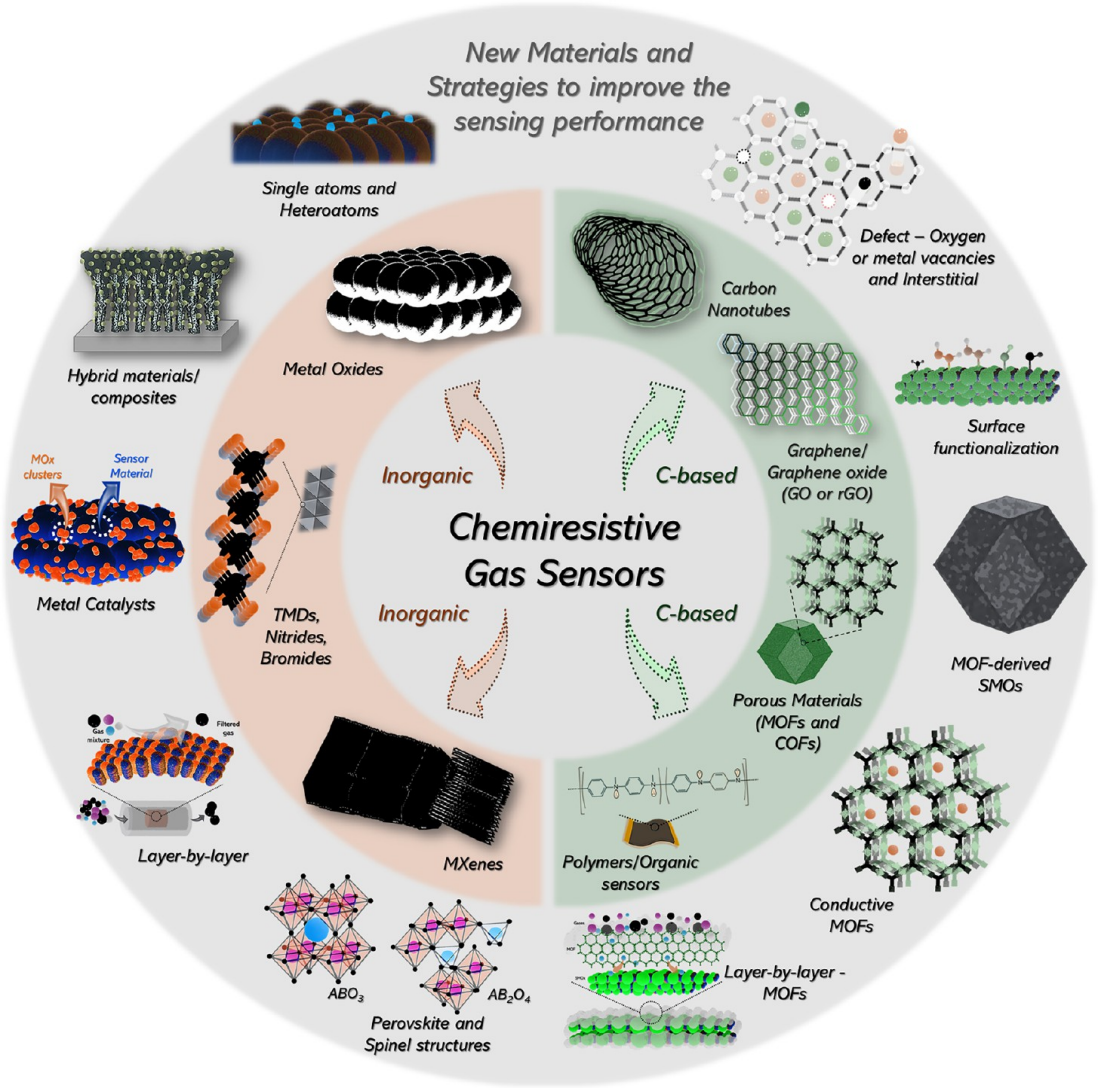
Overview of new materials and design strategies used to develop chemiresistive gas sensors from both carbon-based and noncarbon materials.

Over the past five years, the literature has explored various strategies to modify materials based on advances in engineering of fabrication processes followed by their assembly into sensing devices (Figure 2). These approaches aim to create novel materials with complex structural and electronic composition. For instance, metal oxides and their heterostructures can be manufactured with different approaches, surface-decorated with active single metal atoms or clusters of varying size^204^ and even Janus-like particles:^205^ these topics will be detailed in Sections 3.1.1–3.1.3. Some crystal configurations of chemiresistive materials, such as MXenes, perovskites, and spinel structures that define both the electronic and chemical surface properties, will be elaborated in Sections 3.2 and 3.3. Other materials such as zeolites, MOFs/COFs (conducting polymers and organic materials), and organic functionalizations (MO*_x_*/graphene and/or reduced graphene) are inherently microporous, a key consideration for molecular diffusion and active-site accessibility, as will be discussed in Sections 3.4–3.6. Collectively, these design features are engineered to fine-tune the material’s structural and electronic properties, including porosity, surface area, pore architecture, gas adsorption behavior, and defect types (oxygen and/or metal vacancies), resulting in enhanced detection performance as schematically illustrated in Figure 3.

This review analyzes the progress in the field of chemoresistive gas sensor material design in close comparison to the established exposure limits (Table 1). We elaborate on the merits of synthesis and characterization techniques along with diverse surface and morphology engineering strategies, which have greatly enhanced sensor development and performance. Table 2 identifies chemoresistive materials that have already met (or are promising to meet) air pollutants’ exposure guideline values under laboratory or even real-world conditions. In Table 2, we meticulously identify material composition, morphology, synthesis method, sensor response, target gas concentration (ppm), operating temperature (°C), and relative humidity (RH) conditions, selectivity, and lower limit of detection, as well as response (*t*_resp_) and recovery (*t*_rec_) times.

**Table 2:** Performance Comparison of Material-Based Sensors for Air Pollutants over the Last 5 Years[Table-fn t2fn14]

Toxic gas	Sensing material/morphology	Material synthesis/ Film deposition technique	Sensor response[Table-fn t2fn1] ^–^ [Table-fn t2fn8]	Conc. (ppm)[Table-fn t2fn9]	*T* (°C)/RH (%)[Table-fn t2fn9]	Selectivity[Table-fn t2fn10]	LLOD[Table-fn t2fn11]	*t*_resp_/*t*_rec_ [Table-fn t2fn12]	ref
CO	Pd–CuO Nanorods/SnSe_2_ Nanoflower	Hydrothermal/Spray	[Table-fn t2fn5]34.8	200	rt[Table-fn t2fn13]/10	CH_4_ (−), H_2_S (−), Acetone (−), SO_2_ (−)	–	13 s/58 s	^206^
	Hollow spherical-NiO/MXene	Hydrothermal-etching/Layer-by-layer coating	[Table-fn t2fn1]1.3	400	rt/20	H_2_ (−), CH_4_ (−), SO_2_ (−), CO_2_ (−)	–	8 s/16 s	^207^
	Nanoparticles NiCo_2_O_4_/Ti_3_C_2_O_2_ MXene nanosheets	Hydrothermal-etching/Paste	[Table-fn t2fn5]139.5	100	110	NO_2_ (−), H_2_S, SO_2_ (−), C_2_H_6_ (−), CH_4_ (−), C_3_H_8_ (−), C_2_H_4_ (−), H_2_ (−), CO_2_ (−)	10 ppm	39 s/234 s	^208^
	Boron subphosphide (B_12_P_2_)/-	Calcination in argon-Precipitation/Drop cast	[Table-fn t2fn1]4.013	50	500	Benzene, NH_3_ (−), SO_2_ (−), NO_2_ (−), NO (−)	–	–/–	^209^
	1D Cu_2_DADHA-F2 MOF/nanosheets	Solvothermal/Liquid–liquid interface assembly	[Table-fn t2fn5]63.3	40	rt/10	NO_2_ (4.7), CO_2_ (395.6), H_2_ (204.2), CH4 (6330), dimethyl carbonate (6330), diethyl carbonate (6330), ethyl methyl carbonate (6330)	^[t]^235 ppb	230 s/260 s	^210^
	Sn-Co-based MOF - Co_3_O_4_-SnO_2_/nanoparticle	Precipitation-Annealed/Spin-coating deposition	[Table-fn t2fn2]16.56	100	325/20	H_2_ (10.6), H_2_S (>10.6), NH_3_ (>10.6), CH_4_ (>10.6)	–	3 s/18 s	^211^
	Ga-Co_3_O_4_/nanosheets	Precipitation-ultrasonication-calcination/–	[Table-fn t2fn8]5.9	100	210	H_2_ (2.3), CH_4_ (3.9), NO_2_ (16.9), C_3_H_8_ (4.9), NH_3_ (10.1)	1 ppm	15 s/28 s	^212^
	CuO-SnO_2_/nanotubes	Layer-by-layer assembly on the carbon nanotubes (CNTs) templates, calcination/Drop cast	[Table-fn t2fn5]39.56	300	rt/50	H_2_ (−), NH_3_ (−), NO_2_ (−), H_2_S (−)	^[t]^159 ppb	56 s/23 s	^213^
	Pd-doped SnO_2_ nanoparticles	Flame spray pyrolysis/-	[Table-fn t2fn4]7	1	350/50	Acetone (−), ethanol (−)	^[t]^0.5 ppb	–/–	^214^
CO_2_	5 wt % rGO/CuO–MOF/sheets-nanoparticles	Solvothermal-thermal decomposition/-	[Table-fn t2fn1]39.6	500	rt/40	CO (−), NH_3_ (−), H_2_S (−), NO_2_ (−), CH_4_ (−), Ethanol (−), N_2_ (−)	^[t]^2 ppm	37 s/26 s (300 ppm)	^215^
	Li-Mg-MOF-74/Film	Solvothermal/Drop coating	∼ 90 Hz	500	rt/0	Acetone (>4.5), Methanol (>4.5), Ethanol (>4.5), NH_3_ (>4.5), CH_4_ (>4.5), NO_2_ (>4.5), H_2_ (>4.5),	300 ppm	84 s/69 s	^216^
	Urchin-like TiO_2_- 5 wt % 2D MXene/microsphere	Etching and delamination-Solvothermal-thermal decomposition (N_2_)/Drop casting	[Table-fn t2fn8]30.69	500	30/50	NH_3_ (5.5), Ethanol (10.6), NO_2_ (3.9), CH_4_ (9.3)	^[t]^10 ppm	82 s/92 s	^217^
	MOF-808-Pebax/octahedral shape-membrane	Hydrothermal-sonication/Drop coating	371.8 Hz	1000	rt/–	CH_4_ (−), NH_3_ (−), HCHO (−), Ethanol (−), Acetone (−)	300 ppm	41 s/20 s (5000 ppm)	^218^
	LaCoO_3_-MXene-15 wt %/microsphere-shaped-layers	Hydrothermal-etching/–	[Table-fn t2fn5]50.47	800	60/11	H_2_ (6.7), SO_2_ (9.3), C_2_H_4_ (12.7), CO (13.8), C_2_H_6_ (35.4), CH_4_ (38.1)	50 ppm	14 s/32 s	^219^
	MWCNTs decorated ZnO/nanograins	RF magnetron/e-beam deposition	[Table-fn t2fn2]5.7	5000	150/–	H_2_O_v_ (1.9), CH4 (2.5), NH3 (2.5), Propane (2.3), Toluene (2.1), dimethylformamide (2.3)	100 ppm	670 s/450 s	^220^
SO_2_	Ag-PANI-SnO_2_/nanoparticles	Solvothermal-in situ polymerization-self-assembly method/Paste coating	[Table-fn t2fn5]2010	50	20/0	CH_4_ (−), HCHO (−), Acetone (−), Methanol (−), NH_3_ (−)	<0.5 ppm	110 s/100 s	^221^
	1.0 wt % Cu-rGO-In_2_O_3_/nanosphere-sheet-nanorices	Microwave-assisted hydrothermal + impregnation/Spin coating	[Table-fn t2fn1]11.1	5	200/0	NO_2_ (−), NO (−), CO (−), H_2_S (−), Ethanol (−), C_2_H_4_ (−), H_2_ (−), NH_3_ (−)	<0.5 ppm	57 s/8.5 s	^222^
	Bi_2_O_2_Se/nanosheets	Hydrothermal/Drop cast	[Table-fn t2fn5]34	1	rt/11	NO (>6.8), NO_2_ (6.8), Cl_2_ (>6.8), H_2_ (>6.8), CH_4_ (>6.8), NH_3_ (>6.8), CO (>6.8), CO_2_ (>6.8)	20 ppb	100 s/292.6 s	^223^
	BBTBSe/coral-reef-like	Solvothermal/Drop cast	[Table-fn t2fn1]199.4	100	rt/57	H_2_S (10.7), NO_2_ (7.6), CO (7.97), CO_2_ (5.3), NH_3_ (9.49)	^[t]^0.23 ppb	60 s/70 s	^224^
	Fe_2_O_3_-decorated MoS_2_/nanoparticles-nanoflakes	–/Radio frequency (RF) magnetron sputtering-shadow mask deposition	[Table-fn t2fn5]18.72	1	150/20	CH_4_ (1.63), CO (1.54), NH_3_ (1.49), NO_2_ (1.33)	^[t]^22.8 ppb	152 s/114 s	^225^
	Microporous COF -NKCOF-12/Film	Melt polymerization/Direct fabrication	0.18–0.20	1000	rt/12	Ethanol (10), Acetone (>10), MeOH (>10), NH_3_ (>10), C_2_H_2_ (>10), CH_4_ (>10), CO_2_ (>10), O_2_ (>10), N_2_ (>10)	^[t]^86 ppb	3.47 min/4.03 min	^226^
NO_2_	Ag_2_Te/CeO_2_/nanocubes-hollow roll structure	Hydrothermal/–	[Table-fn t2fn5]30.39	1	65/20	NO (4.5), NH_3_ (14.87), SO_2_ (21.15), CO (−), CO_2_ (−), H_2_ (−), ethanol (−), methanol (−), acetone (−), Methylbenzene (−), benzene (−)	5 ppb	69 s/415 s	^227^
	0.1% Sb-doped SnO_2_/nanoparticles	Hydrothermal/Spin coating	[Table-fn t2fn1]2.65 × 10^4^	1	75/2	NH_3_ (−), H_2_S (−), CO (−)	20 ppb	153 s/11 s	^228^
	gold-black NPs-Ga_2_O_3_/nanorods	Hydrothermal/RF sputtering	[Table-fn t2fn1]5221.1	10	230/30	100 ppm - Ethanol (12), NH_3_ (10)	0.1 ppm	27.3 s/69.2 s	^229^
	WO_3_/W_18_O_49_/branched	Solvothermal/in situ growth	[Table-fn t2fn1]1038	10	50/30	Cl_2_ (−), CH_4_ (−), SO_2_ (−), C_2_H_4_ (−), H_2_S (−), NH_3_ (−), CO (−), CO_2_ (−)	10 ppb	50 s/38 s	^230^
	0.5 wt % HGO/In_2_O_3_/sheet	Hydrothermal/–	[Table-fn t2fn1]2776	1	62.5/20	CO (−), NH_3_ (−), Ethanol (−), Acetone (−), Formaldehyde (−), H_2_S (−)	^[t]^ < 1 ppb	<10 min/<10 min	^231^
	SnSe*_x_*/spherical	Hydrothermal/Spin-coating	[Table-fn t2fn5]1430	5	rt/50	NO_2_ (−), H_2_S (−), SO_2_ (−), NH_3_ (−), Acetone (−), H_2_ (−)	^[t]^∼105 ppt	78 s/178 s	^232^
	Nb-MoS_2_/nanoflakes	Two-zone LP-CVD (Low-Pressure Chemical Vapor Deposition)	[Table-fn t2fn5]18	0.5	rt/80	NO_2_ (−), NO (−), NH_3_ (−), CH_4_ (−), CO (−), CO_2_ (−), SO_2_ (−)	^[t]^0.117 ppb	105.5 s/162.3 s	^233^
	SHI-modified MoS_2_-film	Radio frequency (RF) magnetron sputtering	[Table-fn t2fn5]30.35	50	100/30	NH_3_ (3), H_2_S (8), H_2_ (10), CO_2_ (6)	–	59 s/152 s	^234^
	porous SnO_2_/nanopods	Sn-MOF–Precipitation + calcination/–	[Table-fn t2fn1]64	1	250/90	CO (−), H_2_S (−), Ethanol (−), NH_3_ (−), H_2_ (−), CH_4_ (−)	<10 ppb	15 s/20 s	^235^
	rGO-5% ZnO/-	Pechini Method/Drop cast	[Table-fn t2fn5]22.4	2.5	rt/50	CO (−), H_2_ (−), NH_3_ (−), Toluene (−), Benzene (−), Ethanol (−)	^[t]^2 ppb	12 min/–	^236^
	Cs_2_AgBiBr_6_/SnO_2_/ZnO film/nanorods	Spin coating- Hydrothermal-Vacuum evaporation	[Table-fn t2fn3]2.3	1	rt/60	CH_4_ (≫11), CO (≫11), H_2_ (>11), Ethanol (>11), Acetone (>11), NH_3_ (11), H_2_S (>11), SO_2_ (>11), NO (>11)	–	12 s/9 s	^237^
	TeNT/TeO_2_/nanotubes	Hydrothermal-Thermal oxidation	[Table-fn t2fn5]38.8	0.6	rt/10	NH_3_ (−), Acetone (−), Ethanol (−), Methanol (−), HCHO (−), Toluene (−)	–	39 s/49 s	^238^
	C-MoS_2_/nanosheets	Hydrothermal/Drop cast	[Table-fn t2fn5]2500	10	rt/–	CO_2_ (−), H_2_ (−), H_2_S (−)	^[t]^0.13 ppm	43.1 s/301.2 s	^239^
	Black TiO_2_/burr-like nanorods	Liquid precipitation/Drop coating	[Table-fn t2fn2]3.5	3	rt/30–90	H_2_ (−), Acetone (−), NH_3_ (−), Isopropanol (−), Ethanol (−), HCHO (−), CO_2_ (−)	50 ppb	38 s/–	^240^
	Cu_3_N/nanoparticles	Flame-spray pyrolysis + dry nitridation/–	[Table-fn t2fn8]2.95	1	75/50	Xylene (737), Toluene (>10^3^), Benzene (>10^4^), H_2_ (421), Acetone (>10^3^), Ethanol (>10^3^), NO (3.6), NH_3_ (73.5), H_2_S (>10^3^)	^[t]^0.1 ppb	11 min/99 min	^241^
	Porous WS_2_ Films	Flame spray pyrolysis + dry sulfidation/–	[Table-fn t2fn4]4.5	1	rt/50	NH_3_ (164), NO (217), Acetone (361), H_2_S (>10^3^), CO (>10^3^), N_2_O (>10^3^), Benzene (>10^3^), Toluene (>10^3^), Methanol (>10^3^), Ethanol (>10^3^)	1 ppb	60 s/32 min	^242^
	Porous WO_3_ Films	Flame spray pyrolysis/ flame-deposited directly	[Table-fn t2fn3]110	0.1	125/50	H_2_ (>10^4^), NH_3_ (>10^5^), CH_4_ (>10^4^), Methanol (>10^5^), Ethanol (>10^5^), Acetone (>10^4^), CO (>10^5^), H_2_S (835), Formaldehyde (>10^3^)	3 ppb	7.8 min/43.8 min	^243^
	Carbon nanotube-based	E-beam metal evaporation and in situ chemical vapor deposition	5.6 μW	1	23/45	–	9 ppb	5 min/1 min	^244^
	MoS_2_/RGO-4 heterostructure	Infiltration/–	[Table-fn t2fn5]16.0	1	100/50–60	NH_3_ (−), CO (−), H_2_S (−), Ethylene (−), Formaldehyde (−)	^[t]^0.2 ppb	195 s/279 s	^245^
	Au@ZnO/rGO-2 nanospheres	Solvothermal/–	[Table-fn t2fn1]67.38	1	60/30	Ethanol (11), Acetone (30), NH_3_ (16), Methanol (30), SO_2_ (16), CO_2_ (16)	^[t]^0.138 ppb	248 s/170 s	^246^
	rGO/GO Interfaces	Laser microfabrication reduction/–	[Table-fn t2fn5]18.23	100	rt/dry	H_2_S (−), NH_3_ (−), Acetone (−), Ethanol (−)	^[t]^230 ppb	–/–	^247^
NO	Cu-hemin MOF/rGO	One-pot construction: solvothermal/Drop-drying method	[Table-fn t2fn2]3.42	1	rt/0–75	NH3 (3.2), Acetone (3.3), Ethanol (3.1), Methanol (3.0), Benzene (−), Toluene (−), Ethylbenzene (−), Xylene (−)	0.05 ppm	43 s/367 s	^248^
	Hollow multishelled structured-WO_3_	Emulsion polymerization, hydrothermal calcination/-	1.7	0.05	rt/–	NH3 (11.3), SO2 (17), CO (24.3), H_2_S (−)	^[t]^2.52 ppb	–/–	^249^
	Ni_0.48_Co_0.52_MOF-74/7%-CNT-polyacrylonitrile (PAN)/nanoflowers	Hydrothermal/in situ growth-physical deposition	[Table-fn t2fn5]59	50	rt/40	H_2_S (−), NH_3_ (−), Acetone (−), Ethanol (−), Methanol (−), C_2_H_4_ (−), Hexanal (−), CO_2_ (−)	^[t]^18.6 ppb	39 s/70 s	^250^
	2 mol% Ta-WO_3_/nanoparticles	Solvothermal-calcination/packaging	^a^∼1.75–2.00	0.05	175/–	NH_3_ (−), H_2_S (−), Isoprene (−), Ethanol (−), Acetone (−), CO_2_ (−), CO (−), H_2_ (−), Acetonitrile (−), Acetaldehyde (−), NO_2_ (−), O_2_ (−)	5 ppb	30 s/10 s	^251^
	WO_3_ Thin film/nanorods	Hydrothermal/-	^e^76.95	10	50/–	H_2_ (−), NO2 (−), CO (−)	–	56 s/79 s	^252^
N_2_O	CuO-TiO_2_/nanorods	GLAD method-thermal annealing/-	^e^0.011	1	rt/0	CH_4_ (>5.3), NH_3_ (>5.3), CO (>5.3), NO_2_ (−), H_2_ (5.3), Ethanol (1.9), Acetone (2.0), Toluene (>2.0)	50 ppb	36 s/50 s	^253^
	BaMoO_4_/nanoparticles	Coprecipitation method-calcination/Double-layer YSZ electrolyte (porous layer/dense layer)-coated	25.30 mV	2	375/0–11	CH_4_ (11.1), CO_2_ (9.2), H_2_ (8.9), NH_3_ (6.2), NO (4.5), NO_2_ (5.0)	200 ppb	98 s/552 s	^254^
	TCN(II)-KOH-rGO/CF/2D rod-like amorphous crystals	Hydrothermal/Drop coating	–36.5 μA cm^2^	10	rt	SO_X_ (3.2), CO_2_ (>3.2), Acetone (>3.2), Benzene (>3.2), Tetrahydrofuran (>3.2)	1 ppm	–/–	^255^
NH_3_	BA/MXene/PANI-HCl aerogel/fiber	Wet spinning and etching techniques/Coated	^e^807	100	20/45	Ethanol (−), Methanol (−), Acetone (−), Methanal (−), Toluene (−)	1 ppb	24.1 s/2.2 s	^256^
	Gr/TAPPPANI (GT-2-P)/nanorod-like and lamellar structures and nanoparticles	Polymerization/Drop cast deposition	^e^132.3	100	rt/25	O_2_ (25.4), CO_2_ (186.3), H_2_ (3.8), Ar (57.0), N_2_ (1017.7)	^[t]^1.99 ppm	108 s/1310 s	^257^
	Co-doped ZnFe_2_O_4_-rGO/nanosheets and nanorods	Spray pyrolysis/Spin-coating	^f^81	1	rt/–	Acetone (−), Ethanol (−), isopropanol (−), benzene (−), formaldehyde (−), xylene (−), acetaldehyde (−)	^[t]^0.004 ppb	65 s/18 s	^258^
	Ti_3_C_2_T*_x_*-V_2_O_5_-Ag/open accordion-like lamellar-nanosheet structures and nanoparticles	Hydrothermal and chemical reduction method/Spin coating	^b^19.12	10	rt/25	Toluene (18.4), Acetone (14.2), Isoacetone (18.7), Methanol (17.8), Ethanol (14), H_2_S (12.9)	0.5 ppm	8 s/289 s	^259^
	Bimetallic-Pt_2_Ru_3_@SnO2/nanoparticles-nanosol	Hydrothermal-photochemical reduction/Spin coating	8.2	2	195/80	Acetone (−), H_2_S (−), CO (−), Ethanol (−), H_2_ (−), SO2 (−)	^[t]^5.4 ppb	15 s/532 s	^260^
	PANI/PAN fabric sensor/nanofibers	Electrospinning and preoxidation/Growth	^e^∼160	50	rt/30–40	Ethanol (>30.6), Acetone (>30.6), HCHO (30.6), Methanol (>30.6), IPA (>30.6)	2 ppm	100 s/851 s	^261^
	5CC-COOH-Ti_3_C_2_T*_x_*-PANI/layers	minimally intensive layer delamination (MILD) and molecular self-assembly methods/dip-coated	^e^214.7	80	rt/23	–	^[t]^539 ppb	122.42 s/–	^262^
	P-BNT/needle-shaped	-/coated	^b^32000	40	rt/0	TEA (5694), Ethanol (6882), H_2_S (7240), Toluene (8355), SO_2_ (8579), NO_2_ (13559), Acetone (15385), Avantin (15534), Methanol (18605)	–	40 s/–	^263^
	BN-H/P-BNT/fibrous and needle-shaped	-/coated	^b^718	40	rt/0	TEA (138.1), Ethanol (178.2), H_2_S (101.3), Toluene (388.1), SO_2_ (128.0), NO_2_ (293.1), Acetone (635.4), Avantin (432.5), Methanol (565.4)	^[t]^13 ppb	65 s/25 s	^263^
	SnO_2_ QDs-SnS_2_/nanosheets and quantum dots	Solvothermal/Spin-coating	^b^11.1	100	rt/35	NO_2_ (11.1), CH_4_ (11.1), H_2_ (11.1), Acetone (7.9), Ethanol (6.5)	^[t]^143 ppb	5 s/876 s	^264^
	Fe_2_Mo_3_O_8_-MoO_2_@MoS_2_-900 °C/disks-polyhedral Structures	Hydrothermal-Annealed/Dripped	^e^57	1	rt/5	H_2_S (9.4), NO_2_ (9.5), Methanol (10.3), CO_2_ (10.3), Ethanol (10.8), Acetone (12), Acetaldehyde (15.1), allicin (39.8) and n-hexane (125)	^[t]^3.7 ppb	3 min/60 min	^265^
	Porous CuBr films	Flame spray pyrolysis + dry bromination/–	^c^276	5	rt/90	Isoprene (>30), Ethanol (>38), CH_4_ (>48), Acetone (>56), H_2_ (>57), Acetic acid (>58), Methanol (>65), Formaldehyde (>184), CO (>260)	^[t]^0.210 ppb	2.2 min/50 s (1 ppm)	^45^
O_3_	Cs_2_AgBiBr_6_ perovskite/microsheets	Precipitation/-	^f^1.38	2.3	rt/70	NO (−), H_2_ (−), CO_2_ (−), CH_4_ (−)	–	30 s/<120 s	^266^
	3 wt % Ag-SnO_2_ by MOF/spherical structure	Sol–gel/NASICON- sintered	232.36	0.420	rt/22	N_2_ (−), CO_2_ (−), Ethanol (−), Methanol (−)	180 ppb	–/–	^267^
	Hexagonal-orthorhombic h/o-WO_3_/nanosheets-flake-like structure	Solvothermal-annealed/Paste- hook-and-loop applicator	^a^320.8	3	120/-	NH_3_ (−), Acetone (−), Ethanol (−), Methanol (−)	40 ppb	35 s/29 s	^268^
	IGZO@Mn3O4/nanoparticles	Radiofrequency magnetron sputtering-SILAR methods	^h^4.06	5	rt/73	NH_3_ (−), H_2_ (−), CO (−), NO (−), NO_2_ (−)	0.1 ppm	78 s/184 s	^269^
	CuSCN/polygon-like	Commercial powder/Drop casting technique	^b^1.06	0.015	25/0	–	15 ppb	137.4 s/111 s	^270^
	Spinel CuCo_2_O_4_/nanosheets	Solution combustion method/Pasting	^b^27	1	90/70	NO_2_ (8.4), NH_3_ (>8.4), SO_2_ (>8.4), Acetone (>8.4), Ethanol (>8.4), Methanol (>8.4)	^[t]^8.8 ppb	–/–	^271^
	ZnO/rGO-ZnO/quasi-spheres	Polymerization reaction-Heat treatment/Drop casting	^a^35–40	0.135	250/–	NO_2_ (>27)	135 ppb	10 min/15 min	^272^
	porous CuO/loosely stacked porous structures-nanoparticles	Simple solution combustion method/Drop casting	^b^42	0.050	70/70	H_2_S (>4.5), SO_2_ (>4.5), NO_2_ (4.5), NH_3_ (>4.5), Ethanol (>4.5), Methanol (>4.5), Acetone (>4.5)	10 ppb	<100 s/<30 min	^273^
CH_4_	Cd–In_2_O_3_/porous hollow nanospheres	Impregnation–calcination approach with self-made carbon nanospheres as a hard template-hydrothermal/Slurry-brush	^b^9.5	500	200/–	CO (5.69), HCHO (∼6), Ethanol (∼3.5), Methanol (∼5), Acetone (∼4.5), NH_3_ (∼4.5), Toluene (∼6)	30 ppm	30 s/82 s	^274^
	Al-doped ZnO/nanorods	Precipitation/Slurry-brush	^e^42.4	500	280/0	CO (−), NH_3_ (−), NO_2_ (−), Ethanol (−), Acetone (−), Toluene (−)	100 ppm	3.8 s/5 s	^275^
	N-3DrGO-CuO@Co_3_O_4_/core–shell from MOF	Solvothermal/Drop casting	^e^6.54	100	rt/35	CO_2_ (2.5), H_2_ (2.4), NO_2_ (5.1), Ethanol (4.4), Acetone (3.5)	39.5 ppm	16 s/11 s	^276^
	5.0% Pt-SnO_2_ 45 min/nanospheres	Photochemical deposition-Solvothermal/Drop-coating	^b^30.9	5000	400/20	H_2_ (4.9), Ethanol (>4.9), NH_3_ (>4.9), Toluene (>4.9), Methanol (>4.9)	^[t]^1.18 ppm	1 s/–	^277^
	Pd-PdO-CeO2/Hollow nanospheres-nanodots	Bubble confinement and in situ photochemical deposition/drop-coated	^e^∼70	500	250/74	H_2_ (−), CO (−), NH_3_ (−), NO_2_ (−), H_2_S (−)	^[t]^0.1 ppm	3 s/12 s	^278^
	Pd_2_Pt@m-SnO_2_/Mesoporous-nanoalloys	Solvent evaporation-induced coassembly method/-	^b^9.19	1000	400/–	H_2_ (6.9), CO (7.3), NH_3_ (7.0)	^[t]^0.175 ppm	3 s/37 s	^279^
	SnO_2_-Zn_2_SnO_4_/nanosheets	Hydrothermal/Paste	^b^4.16	500	480/11	SO_2_ (−), H_2_ (−), NO_2_ (−), and CO (−)	5 ppm	1 s/9 s	^280^
	V_2_O_5_-NiO/Nanorods	–	^e^57	4000	200/30	Ethane (−), H_2_ (−), SO_2_ (−), CO (−)	50 ppm	72 s/113 s	^281^
	VO_2_-MoTe_2_/Layered and the silver ear-like special structures	Hydrothermal/drop coating	^e^13	500	rt/11	NH_3_ (−), Benzene (−), Methanol (−), Ethanol (−), Ethane (−), propane (−), H_2_ (−)	500 ppm	75 s/110 s	^282^
	In_2_O_3_/Belts-like	Electrospinning/-	^b^1.1	90	100/0	CO (−), Ethene (−), NH_3_ (−), CO_2_ (−), SO_2_ (−)	^[t]^0.69 ppm	36 s/44 s	^283^
	ZnO/spheres	Hydrothermal/Paste	^b^20.57	5000	rt/30	CO (−), H_2_S (−), NH_3_ (−), Methanol (−)	–	6 s/134 s	^284^
	Pt-SnO_2_-ZnO/double layer structure	Hydrothermal/-	^b^3.36	800	rt/35	H_2_ (2.5), CO (>2.5), NO_2_ (>2.5)	^[t]^12.92 ppm	147 s/132 s	^285^
	AuAg-ZnO/microspheres	Hydrothermal/-	^b^62.61	5000	rt/38	H2 (43.8), CO (50.5), H_2_S (44.7), NH_3_ (40.9), Methanol (12.4)	–	5 s/105 s	^286^
Acetone	SnO_2_-MoS_2_/Nanoparticles	Hydrothermal/Drop casting	^e^13.56	0.1	rt/90	Ethanol (6.6), Methanol (6.6), Toluene (4.4), Benzene (4.4), HCHO (2.2), NH_3_ (1.7)	26 ppb	–/–	^287^
	La_0.8_Ca_0.2_Fe_0.98_Pt_0.02_O_3_ (LCFP) perovskite oxide nanofibers decorated Pt–Fe2O3 nanoparticles	Solvothermal-Electrospinning-Calcination/Drop-coated	39.8	10	250/40	–	0.16 ppm	–/10 min	^288^
	5 wt % Pt-NiFe_2_O_4_/nanorods	Hydrothermal-one-step impregnation/Dripped	221	100	180/50	Toluene (>5.45), *n*-Hexane (>5.45), NO_2_ (>5.45), Methanol (>5.45), HCHO (>5.45), Acetonitrile (5.45)	500 ppb	12 s/13 s (0.5 ppm)	^289^
	B-TiO_2_-SnS_2_/Nanosheets	Hydrothermal/Paste	^b^15.1	20	rt/30	NH_3_ (−), CO (−), NO_2_ (−), Ethanol (>6), H_2_S (−)	^[t]^757 ppb	6.7 s/9.8 s	^290^
	Ti_0.5_Sn_0.5_O_2_/Nanoparticles	Hydrothermal/Slurry-coating	^b^26.83	100	200/–	Propylene glycol (7.5), Ethylene glycol (15.8), Acetaldehyde (10.8), NH_3_ (22.4)	100 ppb	1 s/12 s	^291^
	MgCr_2_O_4_/Spherical	Sol–gel-calcination/Hollow cylindrical alumina tube-Spin coating	^e^1570	5	160/0	Ethanol (−), NH_3_ (−), CO (−), Isoprene (−), H_2_S (−)	100 ppb	8.5 s/–	^292^
	WO_3_-MoS/flower-like sphere	Hydrothermal/-	^b^9.4	100	132/15	Methanol (−), Ethanol (−), Toluene (−), NO_2_ (−), NH_3_ (−)	^[t]^79.46 ppb	4 s/7 s	^292^
	Co_0.57_Fe_2.43_O4/Nanoparticles	Hydrothermal/-	^b^348.6	100	150/50	Methanol (>5.4), Ethanol (>5.4), Glycol (>5.4), HCHO (5.4)	^[t]^44.7 ppb	–/85 s	^293^
	3DIO NiO-SnO_2_/3D ordered macroporous architecture	Assembly-Photonic crystal template-Calcination/Paste-coating	^b^202	100	198.5/25	H_2_ (−), NH_3_ (−), CH_4_ (−), Methanol, Ethanol (>4)	^[t]^0.136 ppm	3 s/57 s	^294^
	MnFeCoNiCu (HEA)-loaded SnO_2_/Nanoparticles with granular morphology	Low-temperature oil-phase synthetic strategy-wet impregnation/–	^b^4.17	0.5	230/40	H_2_ (2.96), N_2_O (3.29), NO_2_ (3.13), Ethanol (2.16), HF (2.78), NH_3_ (2.55), CHCl_3_ (2.70), HCHO (2.67), H_2_S (2.90)	^[t]^30 ppb	4.6 s/5 s	^295^
	B–Co_3_O_4_/Wrinkled layered structures	Precipitation-Thermal treatment/Slurry-coating	^a^105.6	100	190/–	Ethanol (−), HCHO (−), Methanol (−), NH_3_ (−)	20 ppb	291 s/83 s	^296^
	β-Bi_2_Sn_2_O_7_-ZnO/Bilayer	Ultrasonic spray pyrolysis-Hydrothermal-Sintering/Spin coating	^b^17.2	50	280/30	Ethanol (2.7), Benzene (9.8), HCHO (7.2), Toluene (6.1), Methanol (4.8)	^[t]^11.4 ppb	13 s/– (5 ppm)	^297^
	0.5 wt % Ir-loaded In_2_O_3_/Nanoparticles	Flame spray pyrolysis/Spin coating	^b^6.02	1	300/0	Methanol (>2.5), Ethanol (>2.5), H_2_S (>2.5), Methyl mercaptan (>2.5), Dimethyl sulfide (>2.5), NO_2_ (>2.5), CH_4_ (>2.5), C_2_H_2_ (>2.5), H_2_ (>2.5), NH_3_ (>2.5), CO_2_ (>2.5), Propionic acid (>2.5), Acetic acid (>2.5), Butyric acid (>2.5)	^[t]^10.7 ppb	42 s/–	^298^
	MOF-derived Ce-NiO/Nanowalls	Hydrothermal-Annealed/-	^a^4.2	10	175/20	Methanol (−), Ethanol (−), NH_3_ (−), Acetaldehyde (−), Isoprene (−), Toluene (−)	1 ppm	8 s/10 s	^299^
	0.5% Al-W_18_O_49‑x_/2D circular nanorod arrays	Solvothermal/-	^b^65	50	200/30	Triethylamine (>3), Toluene (>3), 3H-2B (>3), HCHO (>3), Ethanol (>3), Methanol (>3), NH_3_ (>3), H_2_ (>3), H_2_S (>3)	10 ppb	8 s/24 s	^300^
	3DOM-Co_5%_O_x_ *-*ZnO/Macroporous structure	Impregnation-Crystallization-Pyrolysis-Annealed/Slurry-brushed	^b^322	30	rt/35	Isopropanol (>8), Toluene (>8), Acetic acid (>8), Formic acid (>8), Formaldehyde (>8), NH_3_ (>8), Methanol (>8), Ethanol (>8), H_2_S (>8), cyclohexanone (>8)	^[t]^10.6 ppb	8 s/12 s	^301^
	Co(OH)F-CQDs/hexagonal structure with hollow center	Hydrothermal/-	^a^13	100	120/40	Butanone (4), Formic acid (6.8), NH_3_ (7.6), Ethanol (4.2), Toluene (7.2)	200 ppb	130 s/135 s	^302^
	K-Sn-Co_3_O_4_/porous microsphere	Solvothermal reaction-Annealing treatment/Slurry	^a^100	100	110/18	Methanol (−), HCHO (−), Nitrobenzene (−), Benzene (−), NH_3_ (−), CO_2_ (−)	100 ppb	20 s/25 s	^303^
	SrFeO_3_-Ti_3_C_2_T*_x_*/Wrinkled and layered morphology associated with delaminated MX nanosheets	Electrostatic self-assembly method/Drop casting	^e^99.7	100	rt/30	NH_3_ (2.6), H_2_O (5.7), Ethanol (7.5), Methanol (8.5), Formalin (8.4)	250 ppb	7 s/17 s	^304^
	ZnO@MWCNTs-PANI/Nanoparticles-nanotubes	*In situ* oxidative polymerization-flame spray pyrolysis/Hot-spraying method	^d^0.26	20	150/2	Toluene (−), Ethanol (−)	0.2 ppm	150 s/250 s	^305^
	CoFe_2_O_4_-TiO_2_@MXene-C/Spherical nanoparticles-layers	Hydrothermal/Paste	^b^52	100	185/25	NH_3_ (−), HCHO (−), Methanol (−), Ethanol (−), Dimethylfumarate (−)	^[t]^70 ppb	12 s/58 s	^306^
	Single-atom 8%Sn-doped ZnO/nanosheet	Ball-milling, followed by transformation and post-treatment-calcination/Drop casting	^b^213	10	290/20	Ethanol (2.2), Methanol (4), HCHO (9.1), CO_2_ (27.3), N_2_ (53.9)	^[t]^0.52 ppb	550 s/72 s	^307^
	SnO_2_-MoS_2_/Nanoparticles	Hydrothermal/Drop casting	^e^13.56	0.1	rt/90	Ethanol (6.6), Methanol (6.6), Toluene (4.4), Benzene (4.4), HCHO (2.2), NH_3_ (1.7)	^[t]^26 ppb	–/–	^287^
	La_0.8_Ca_0.2_Fe_0.98_Pt_0.02_O_3_ (LCFP) perovskite oxide nanofibers decorated Pt–Fe2O3 nanoparticles	Solvothermal-Electrospinning-Calcination/Drop-coated	^h^39.8	10	250/40	–	0.16 ppm	–/10 min	^288^
	5 wt % Pt-NiFe_2_O_4_/nanorods	Hydrothermal-one-step impregnation/Dripped	^b^221	100	180/50	Toluene (>5.45), *n*-Hexane (>5.45), NO_2_ (>5.45), Methanol (>5.45), HCHO (>5.45), Acetonitrile (5.45)	500 ppb	12 s/13 s (0.5 ppm)	^289^
	B-TiO_2_-SnS_2_/Nanosheets	Hydrothermal/Paste	^b^15.1	20	rt/30	NH_3_ (−), CO (−), NO_2_ (−), Ethanol (>6), H_2_S (−)	757 ppb	6.7 s/9.8 s	^290^
	Ti_0.5_Sn_0.5_O_2_/Nanoparticles	Hydrothermal/Slurry-coating	^b^26.83	100	200/–	Propylene glycol (7.5), Ethylene glycol (15.8), Acetaldehyde (10.8), NH_3_ (22.4)	100 ppb	1 s/12 s	^308^
	MgCr_2_O_4_/Spherical	Sol–gel-calcination/Hollow cylindrical alumina tube-Spin coating	^e^1570	5	160/0	Ethanol (−), NH_3_ (−), CO (−), Isoprene (−), H_2_S (−)	100 ppb	8.5 s/–	^292^
	Pt/PtO_x_-decorated Al_2_O_3_ + Si/WO_3_ nanoparticles	Flame spray pyrolysis/–	^d^15.8	1	rt/90	Acetaldehyde (315), H_2_ (789), Isoprene (263), CO (>1000), Methanol (263), Ethanol (>1000), Formaldehyde (225), 2-propanol (>1000)	^[t]^2 ppb	–/–	^309^
	Pt/Al_2_O_3_ + Si/WO_3_ nanoparticles	Flame spray pyrolysis/–	^d^4.3	1	135/90	Ethanol (>500), Isoprene (>1000), H_2_ (>250), CO (>1000), NH_3_ (>1000)	^[t]^5.5 ppb	55 s/100 s (500 ppb)	^310^
CH_2_O	PtCu/In_2_O_3_ Hexagonal Hollow Nanotubes	MOF-derived solvothermal and bimetallic loading/–	^a^148.29	50	90/–	NH_3_ (−), Benzaldehyde (−), Acetone (−), Toluene (−), Methanol (−), Ethanol (−), Acetaldehyde (−), Benzene (−), H_2_S (−)	50 ppb	6 s/9 s	^311^
	In_2_O_3_@TiO_2_ Double-Layer Nanospheres	Water-bath using a carbon template/Coated (slurry)	^a^3.8	1	rt/25	Acetone (−), NH_3_ (−), Methanol (−), Ethanol (−), Toluene (−), Benzene (−)	100 ppb	28 s/50 s	^312^
	Pt/Rh/SnO_2_ Hollow Nanotubes	Hydrothermal using a carbon template/Coated	^a^265.8	25	200/–	NH_3_ (−), Ethanol (−), Methanol (−), Acetone (−), Acetaldehyde (−), Benzaldehyde (−), Triethylamine (−), Trimethylamine (−)	1000 ppb	2.6 s/6.1 s	^313^
	Ru-doped CeO_2_ Nanoparticles	Chemical coprecipitation/Drop-casting-enabled spin-coating method	^f^5.04	5	25/–	Methanol (3), Ethanol (3), Propane-2-ol (2), Aniline (2), Ethylamine (2)	10 ppb	3.33 s/3.58 s	^314^
	SnO_2_/SnSe_2_ Honeycomb	Hydrothermal/Coated	^a^13.47	10	150/–	Acetone (−), Trimethylamine (−), H_2_S (−), Ethanol (−), CO (−), NO_2_ (−), benzene (−)	100 ppb	63 s/12 s	^315^
	La_0.9_Fe_x_Sn_1‑x_O_3_ Hollow Microspheres	Hydrothermal/Paste	^a^161	20	200/–	Benzene (−), Toluene (−), Xylene (−), Acetone (−), Methanol (−), Ethanol (−)	500 ppb	52 s/248 s	^316^
	Mixed-Phase In_2_O_3_ Nanoparticle	Solvothermal/Paste + coated	^a^330	50	120/–	Methanol (6.6), Ethanol (6.1), Triethylamine (3.5), Toluene (5.9)	^[t]^11 ppb	12 s/355 s	^317^
	CuO_x_ clusters/Co_3_O_4_ Nanoparticles	Flame spray pyrolysis/flame-deposited directly	^c^4.6	1	75/50	Acetone (7.3), Toluene (19), Ethanol (5.5), NH_3_ (58), CO (89), CH_4_ (52)	3 ppb	26–51 min (3–1000 ppb)	^318^
	PEI-doped In_2_O_3_ Nanospheres	Hydrothermal/Paste	^b^420.1	105	110/–	Ethanol (>30), Methanol (>30), Acetone (>30), Acetaldehyde (>30), Acetic acid (>30), Formic acid (>30), NH_3_ (>30), H_2_S (>30)	50 ppb	1.9 s/233 s	^319^
	Flower-like Microsphere ZnCo_2_O_4_/In_2_O_3_	Hydrothermal/Coated	^b^107.3	100	258/–	Acetone (>4.68), Ethanol (>4.68), Methylbenzene (>4.68), NH_3_ (≫ 4.68), Cyclohexane (≫ 4.68)	^[t]^157 ppb	51 s/52 s	^320^
	In-doped LaFeO_3_ Nanoparticles	Sol–gel/Paste	^a^122	100	125/–	Ethanol (4.9), Toluene (≫ 4.9), Xylene (≫ 4.9), Acetone (>4.9), NO (≫ 4.9), NO_2_ (≫ 4.9), CO (≫ 4.9), NH_3_ (≫ 4.9)	1 ppb	36 s/40 s	^321^
	Tb-doped SnO_2_ Composite	MOF-derived Solvothermal/Paste + coated	^b^209.3	10	200/80	Methanol (>10), Acetone (>10), Triethylamine (>5), Ethanol (>5)	^[t]^0.251 ppb	28 s/135 s	^322^
	ZnO Quantum Dots/ZnSnO_3_ Nanocubes	Sol–gel/–	^b^613.3	100	70/–	Formic acid (>10), Ethanol (>10), Acetaldedhyde (>10), Methanol (>10), NH_3_ (>10), Acetone (>10), Acetic acid (>10)	100 ppb	2 s/436 s	^323^
	Laminar SnO_2_	Sn-MOF@SnO_2_-derived Water bath/Screen printing	^b^10000	10	120/–	Triethylamine (>70), Ethanol (>534), Toluene (≫ 534), Acetone (≫ 534), Xylene (≫ 534), Methanol (≫ 534), NH_3_ (≫ 534), NO_2_ (≫ 534)	<10 ppb	33 s/142 s	^324^
	Au Nanocage/In_2_O_3_ Nanoparticle	Solvothermal/Slurry + coated	^b^75.6	50	140/–	Methanol (≫ 15), Ethanol (≈15), Acetone (>15), NH_3_ (≫ 15), Benzene (≫ 15)	25 ppb	25.6 s/68.1 s	^325^
	Polypyrrole-Encapsulated MoO_3_ Hollow Nanostructures	Hydrothermal and cation-exchange-assisted Kirkendall effect/Slurry + drop coating	^b^6.5	100	rt/65	Triethylamine (−), Ethanol (−), Methanol (−), Acetaldehyde (−), Acetone (−), NH_3_ (−), NO_2_ (−), CO (−), Benzene (−), Toluene (−), Xylene (−)	500 ppb	13.3 s/46.37 s	^326^
	SnMOF/SnO_2_@TiO_2_ Nanotube Arrays	Solvothermal/–	^b^1.7	6	rt/–	Methanol (>3), Ethanol (>3), Benzyl alcohol (>3), Acetonitrile (>3), Acetone (>3)	^[t]^80 ppb	4 s/2.5 s	^327^
Acetaldehyde	NiO Nanosheets-WO_3_ Nanorods	Hydrothermal/–	^e^22.4	100	250/–	CO_2_ (−), CO (−), NO_2_ (−), H_2_S (−)	–	1177 s/632 s	^328^
	SnO_2_ Nanoparticles	MOF-derived hydrothermal/Coated	^b^10.69	40	100/–	Formaldehyde (1.6), Ethylene glycol (2.3), Acetone (>2.3), Acetic acid (>2.3), Ethanol (>2.3)	50 ppb	3 s/4 s	^329^
	CuO NPs/rGO Composite	Hydrothermal/–	^e^82.67%	100	rt/70	CO (6.2), NO_2_ (19.6), Methanol (), Acetone (>19.6), Ethanol (>19.6), Isoacetone (>19.6), n-Propionaldehyde (>19.6), n-Butyraldehyde (4.7), NH_3_ (3.1)	^[t]^95.1 ppb	22 s/159 s	^330^
	MoS_2_ QDs/PEDOT: PSS	Hydrothermal and in situ polymerization/Drop coating	^e^78.8%	100	rt/–	Methanol (−), Ethanol (−), Acetone (−), Tetrahydrofuran (−), Formaldehyde (−)	1000 ppb	137 s/99 s	^331^
H_2_S	CsPbBr_3_ perovskite	Antisolvent/Drop casting	^a^4.76	5	rt/–	NO_2_ (>4), NH_3_ (>4), Ethylene (>4), CO (>4), SF_6_ (>4)	200 ppb	73.5 s/275.6 s	^332^
	Ru@SnO_2_ Nanospheres	Solvothermal and deposition–precipitation/Drop coated	^b^310.1	20	160/–	Acetone (−), NH_3_ (−), NO_2_ (−), SO_2_ (−), CH_4_ (−), H_2_ (−), Ethanol (−), Trimethylamine (−)	100 ppb	<1 s/47 s	^333^
	BVO/Ag spindles	Hydrothermal and wet impregnation/Paste - brush	^b^31.2	50	100/–	CO_2_ (>10), CO (>10), SO_2_ (>10), NH_3_ (>10), H_2_ (>10), CH_4_ (>10), Acetone (>10), Ethanol (>10)	100 ppb	24 s/27 s	^334^
	Co_3_O_4_/ZnO Hollow Nanofibers	MOF-derived electrospinning/-	^b^45	0.2	325/–	NH_3_ (−), Ethanol (−), Xylene (−), NO_2_ (−), SO_2_ (−), Formaldehyde (−), Acetone (−)	200 ppb	88.7 s/110.6 s	^335^
	CuWO_4_-WO_3_ Nanofibers	Electrospinning combined with a sacrificial template/Drop-coating	^b^60.8	5	200/–	CO (>50), Acetone (>50), H_2_ (>50), Toluene (>50)	100 ppb	283 s/151 s	^336^
	Cu_2_O-CuFe_2_O_4_ Nanoarrays	Electrochemical in situ assembly/Vacuum ion sputtering	^g^243.5	0.01	rt/20–90	Ethanol (≫ 50), Methanol (≫ 50), Butanol (≫ 50), H_2_ (≫ 50), Ethylene glycol (≫ 50), NH_3_ (≫ 50), CO (≫ 50), SO_2_ (≫ 50), Acetone (≫ 50), NO_2_ (>50)	10 ppb	283 s/–	^337^
	WO_3_/CuO Nanocomposite	Electrospinning/Brush-coated	^e^70	5	150/–	Ethanol (−), NH_3_ (−), Trimethylamine (−), Acetone (−)	500 ppb	24 s/78 s	^338^
	CdS-Co_3_O_4_ Porous Nanoflower Spherical	Hydrothermal and liquid-borne ultrasound assistance/Drop-coated	^e^104	10	rt/43	NO_2_ (−), H_2_ (−), CO (−), NH_3_ (−), Methanol (−), Ethanol (−), Acetone (−), Xylene (−), Triethylamine (−), DMF (−)	200 ppb	86 s/51 s	^339^
	Fe_2_O_3_/Ti_3_C_2_ Nanostructure	In situ self-assembly/Paste - brush	^e^118	20	rt/30	NH_3_ (−), Methanol (−), Acetone (−), Ethanol (−), Benzene (−), CO (−), NO_2_ (−), CH_4_ (−), Formaldehyde (−)	10 ppb	<10 s/<15 s	^340^
	Flower-petal-like Au/SnO_2_ Nanostructure	Sol–gel/Spin-coated	^g^270	0.5	rt/17.5	Acetone (−), NH_3_ (−), CO (−), N_2_ (−), NO (6.95), NO_2_ (3.39)	2 ppb	30 s/126 s	^341^
	MoSe_2_@SnO_2_ Nanocomposite	Electrospinning and hydrothermal/Drop casted	^e^19.24	500	rt/–	NH_3_ (−), NO_2_ (−), CO_2_ (−), CH_4_ (−)	^[t]^15 ppb	35 s/64 s	^342^
	ZnO Nanoparticles	Coprecipitation/Slurry - loaded	^b^26	1	220/–	Ethanol (−), CHN (−), CF_2_H_2_ (−), Acetone (−), Propane (−), Toluene (−), CH_4_ (−), NH_3_ (−), CO (>13), Ethylene (−), Butane (−)	50 ppb	72 s/29 s	^343^
	CuO-WO_3_ Microflowers	Hydrothermal/–	^b^3511.1	10	60/–	SO_2_ (−), CO (−), NO_2_ (−), Methanol (−), Ethanol (−), Ethylene glycol (−)	^[t]^1 ppb	4 s/–	^344^
	CuO Nanoflowers	Laser ablation/Electrohydrodynamics inkjet printed	^a^2.85	0.1	rt/–	CO (−), NH_3_ (−), Ethanol (−), Acetone (−), NO_2_ (−)	10 ppb	250 s/–	^345^
	MoO_3_ Nanobelts decorated with MnO_2_ Nanoparticles	Hydrothermal/Drop-casted	^b^32.51	100	rt/–	H_2_ (−), NO_2_ (−), CO (−), NH_3_ (−), SO_2_ (−)	^[t]^4.58 ppb	40 s/42 s	^346^
Methanol	Carbon Nanofibers/NiCo_2_O_4_ Films	Hydrothermal/–	^e^36.4	50	150/–	Ethanol (5.9), Xylene (8.5), Acetone (7.9), Hexane (9.6), Butanol (10.4), Acetidin (10.4), Dichloromethane (19.2)	–	96 s/116 s	^347^
	Ag-LaFeO_3_@ZnO-Pt Core–shell Sphere	Hydrothermal/Screen printing	^a^453.02	5	86/–	Formaldehyde (>3.6), Ethanol (≫ 3.6), NH_3_ (≫ 3.6), Toluene (≫ 3.6), Acetone (≫ 3.6), Benzene (≫ 3.6), Xylene (≫ 3.6), Triethylamine (≫ 3.6)	^[t]^3.27 ppb	81 s/79 s	^348^
	In_2_O_3_ Nanocubes/Ti_3_C_2_T*_x_* MXene Composites	Hydrothermal self-assembly/Slurry - brush	^e^29.6	5	rt/–	Acetone (−), Xylene (−), Triethylamine (−), Trimethylamine (−), Toluene (−)	–	6.5 s/3.5 s	^349^
	ZnO Quantum Dots Decorated Carbon Nanotubes	Sparking and thermal chemical vapor/CNT - directly grown + drop-coated	^e^15.8	500	rt/–	Acetone (−), Dimethylformamide (−), NH_3_ (−), Ethanol (−), Formalin (−), Toluene (−)	–	49 s/26 s	^350^
	Hollow Urchin-like Ag-doped In_2_O_3_ Nanomaterial	Solvothermal/Paste - coated	^b^10.9	50	200/–	Ethanol (−), Isopropanol (−), Ethanediol (−), NH_3_ (−), Toluene (−), Acetone (−)	–	2.4 s/9 s	^166^
	0.1CeO_2_-coated SnO_2_ monolithic bilayer	Ultrasonic spray pyrolysis and screen-printing	^d^3.5	5	400/–	Ethanol (16.8), Formaldehyde (−), Acetone (−), CO (−), NH_3_ (−)	^[t]^0.021 ppm	4 s/–	^351^
	Porous LaFeO_3_ nanoarchitectures	Precipitation and calcination/Thin-film coating + micro-welding process	^a^300	100	150/40	Triethylamine (>20), NH_3_ (>250), Xylene (>100), Acetone (>100), Ethanol (>100), Formaldehyde (>100), Isopropanol (>50)	–	23 s/25 s	^352^
	Pd-SnO_2_ microsensor	Microfabricated film integrated with a Tenax TA separation column/–	^d^1.72	148 (breath sample)	rt/100	Ethanol (−) (breath sample)	10 ppm	<2 min/15 min (10–1000 ppm)	^353^
Ethanol	Fe_2_O_3_/Fe_2_(MoO_4_)_3_ Composite	Microwave-assisted and *in situ* solid-state reaction/Paste	^b^12.8	50	200/–	Methanol (>4), NO (>4), CO (>4), NH_3_ (>4), H_2_S (>4), Isopropanol (>4), Formaldehyde (>4), Benzene (>4)	1000 ppb	5 s/30 s	^354^
	In_2_S_3_/In_2_O_3_/In_2_S_3_ Hollow Nanofibers	Electrospinning and postvulcanization/Slurry - coated	^b^23	100	200/–	NO_2_ (1.82), Methanol (2.49), Acetone (11.79), NH_3_ (18.25), Toluene (19.66)	–	1 s/25 s	^355^
	Au Nanoparticle-Adsorbed ZnO Nanorod Arrays	Hydrothermal and photochemical deposition/RF sputtering + HTM process + Ag evaporation	^e^86.9	100	270/–	Methanol (−), Isopropanol (−), Acetone (−)	–	3.39 s/179.38 s	^356^
	Mesoporous In_2_O_3_-ZnO Hierarchical Structure	Hydrothermal/Paste - brush	^b^35	100	225/–	NO_2_ (>3.1), Acetone (>3.1), Methanol (≫ 3.1), Benzene (≫ 3.1), Toluene (≫ 3.1), CO (≫ 3.1), H_2_ (≫ 3.1), CH_4_ (≫ 3.1)	200 ppb	4 s/90 s	^357^
Benzene	Au-ZnO/exfoliated WSe_2_	Hydrothermal and liquid-phase exfoliation/Layer self-assembly technique	^e^520	50	rt/–	Toluene (>6), NH_3_ (>6), Xylene (>6), Ethanol (>6), Formaldehyde (>6), Methanol (≫ 6), Acetone (≫ 6), H_2_S (≫ 6), NO_2_ (≫ 6), SO_2_ (≫ 6), O_3_ (≫ 6)	–	47 s/70 s	^179^
	Pd doped CoTiO_3_/TiO_2_ Nanospheres	Hydrothermal/Screen printing	^a^33.46	50	rt/–	NH_3_ (−), Formaldehyde (−), Ethanol (−), Acetone (−)	100 ppb	49 s/9 s	^358^
	Raisin(Pd-Co_3_O_4_)-Bread(SnO_2_) Structure Film	Ultrasonic spray pyrolysis/Screen-printing	^d^24.3	5	325/–	Ethanol (>2.15), Formaldehyde (>2.15), CO (≫ 2.15), Toluene (2.15), Xylene (>2.15)	^[t]^4.4 ppb	8 s/50 s	^359^
	Sr-CeO_2_ Nanopetals	Coprecipitation/Drop-casting aided spin-coating technique	^f^5.40	50	rt/–	Aniline (4.15), Isopropanol (3.75), Methanol (3.25), Ethanol (3.14), Acetone (2.95)	–	28 s/29 s	^360^
	Au-Pt Nanoparticle-supported ZnO Porous Nanobelts	In situ thermal oxidation/Coated	^b^39	50	300/–	Dimethylformamide (−), Methanol (−), Formaldehyde (−), Ethyl ether (−), Acetone (−), NH_3_ (−)	<100 ppb	8 s/30 s	^361^
	WO_3_-Pd/SnO_2_ nanoparticles	Flame spray pyrolysis/–	^d^2.1	1	260/50	Xylene (>200), Toluene (>200), Acetone (>200), Acetaldehyde (>200), Isoprene (>200), Methanol (>200), Ethanol (>200), CO (>200), H_2_ (>200), Ethylbenzene (>200)	0.013 ppm	36 s/47 s (100 ppb)	^362^
	CoCu_2_O_3_-Pd/SnO_2_ nanocrystals	Flame spray pyrolysis/–	^h^1.35	1	170/50	Toluene (−), Xylene (−), Isoprene (−), Acetone (−), Ethanol (−), Methanol (−), H_2_ (−), NO (−), CO (−), NO_2_ (−), Formaldehyde (−), N_2_O (−)	12 ppb	3.8 ± 0.7 min/7.3 ± 0.9 min	^363^
Toluene	Co_3_O_4_/ZIF-67 composite	Microwave-assisted hydrothermal and reflux/Drop casting	^a^61.22	100	250/–	Methanol (>5.43), Acetone (>5.43), 2-butanone (5.43), m-xylene (>5.43), Benzene (>5.43)	–	93.2 s/224.3 s	^364^
	SnO_2_@Co_3_O_4_ Nanospheres	Hydrothermal/Drop casting	^a^10.1	100	300/–	Methanol (1.8), Ethanol (2.3), Acetone (2.3), H_2_S (3.9)	^[t]^831 ppb	25 s/10 s	^365^
	Au/ZnO-Al	Deposition–precipitation/Dropping	^b^18.3	5	275/–	Ethanol (3.1), Acetone (3.5), NH_3_ (3)	^[t]^1.1 ppb	–	^366^
	Urchin-like ZnFe_2_O_4_ Spheres	Solvothermal/Paste coated - brush	^b^79	100	250/–	Acetone (−), Ethanol (−), Methanol (−), Formaldehyde (−), Xylene (−)	200 ppb	3 s/208 s	^367^
Xylene	CVD ZnO Nanorods	Thermal CVD/–	^b^44.6	200	300/–	Formaldehyde (−), Ethanol (−), Benzene (−), Toluene (−), Methylamine (−), Dimethylamine (−)	–	16 s/48 s	^368^
	NiO-yolk Triple-shell Microspheres	MOF-derived Microwave-assisted solvothermal/Drop casting	^e^217.5	100	350/dry	Methanol (5.53), 2-butanone (2.62), 3-methyl-1-butanol (5.53), Acetone (2.92), Ethanol (6.18)	^[t]^5.43 ppb	89 s/191 s	^369^
	CuFe_2_O_4_ Nanotubes	Electrospinning and liquid precipitation/Coating	^b^38.1	100	260/30	Methanol (>3), Ethanol (>2), Acetone (>2), Formaldehyde (>3), Benzene (4), Toluene (2)	^[t]^380 ppb	<5 s/(594 ± 40) s	^370^
	Sn-SnO_2_ Nanocomposite	Solvothermal/Drop casting	^e^255	60	rt/40	Toluene (−), Ethylbenzene (−), Benzene (−), Ethanol (−), Acetone (−)	1.9 ppm	1.5 s/40 s	^371^
	Mo-doped Co_3_O_4_ Nanorods	MOF-derived solution preparation/Coating	^a^29.8	100	140/–	NH_3_ (>2.1), Ethanol (2.1), Isopropanol (>2.1), Acetone (>2.1), Toluene (>2.1), Methanol (>2.1), Formaldehyde (>2.1), CO (≫ 2.1), NO (≫ 2.1), NO_2_ (≫ 2.1)	500 ppb	232 s/744 s	^372^

a For the literature survey, we deployed Google Scholar and Web of Science search engines. Search terms: “gas sensor”, “chemoresistive”, “environmental monitoring” “hazardous analyte”, and the respective pollutant names, e.g., “NO_2_”, “CO”, “SO_2_”. Only articles from the last 5 years on chemoresistive gas sensors were considered, demonstrating LLODs compliant with the regulatory values given in Table 1. Note that different sensor response definitions are given in the literature, as identified by footnotes *b*–*i*. In cases where the response was not defined in the original report, no footnote is provided.

b *R*_g_/*R*_a_.

c *R*_a_/*R*_g_.

d *R*_g_/*R*_a_ – 1.

e *R*_a_/*R*_g_ – 1.

f [(*R*_g_ – *R*_a_)/*R*_a_] × 100.

g *I*_g_/*I*_a_.

h (*I*_g_ – *I*_a_)/*I*_a_.

i (*R*_g_ – *R*_a_)/*R*_a_.

j Conc. = concentration; *T* = temperature; RH = relative humidity.

k Selectivity.

l LLOD = lower limit of detection. Values with no superscript are experimental values; those with superscript “[t]” are theoretical values.

m *t*_resp_ = response time; *t*_rec_ = recovery time.

n rt = room temperature.

### 3.1 Metal-Oxide (MO*_x_*) Sensors and Strategies to Improve Toxic Volatile Detection

As can be noted in Table 2, either pristine or heterostructured/doped semiconductor MO*_x_*’s are widely deployed across most of the air pollutants, with the notable exception of CO_2_, where MO*_x_*’s are either interfaced with other materials classes (e.g., MOFs, MXenes, CNTs), or very different sensor concepts (e.g., optical^373^) are preferred. Next, we examine the theory behind chemoresistivity, with particular emphasis on the most studied MO*_x_*, which should share similarities in principle with other semiconductor materials relevant to quality monitoring. After discussing some fundamentals, we explain redox chemistry in *microscopic* relation to electronic band structure and space-charge effects, followed by a critical review of the mechanisms triggering such electrical responses, in light of recent *operando* spectroscopic investigations.

#### 3.1.1 Theory of MO*_x_* Semiconductors

In ideal, defect-free, and perfectly stoichiometric oxide crystals, the Fermi level (*E*_F_) lies approximately at midgap between the valence band maximum (VBM) and conduction band minimum (CBM), deviating only slightly—typically by a few tens of meV, toward one band edge, depending on the relative effective masses of holes and electrons in the VB and CB, respectively. Owing to the absence of native donors (e.g., oxygen vacancies in n-type MO*_x_*)^71^ or acceptors (e.g., cation vacancy in p-type MO*_x_*),^374^ such a purely *intrinsic semiconductor* would exhibit negligible free-carrier density and therefore function neither as an effective chemoresistor, nor as a suitable material for other surface-redox processes, such as heterogeneous catalysis^375^ or electrochemical energy conversion.^376^ In fact, for these applications, native defects are essential, as they (i) provide the donor–acceptor imbalance necessary for carrier modulation *via n*- or *p*-doping, and (ii) introduce intrinsic nonstoichiometry at the reactive surface, which typically fosters the adsorption, activation, and conversion of air pollutant molecules, i.e., the receptor function.^377^

Beyond native oxygen vacancies (*n*-doping) or interstitials (*p*-doping), doping small quantities of aliovalent metals into the MO*_x_* lattice also introduces donors or acceptors (depending on relative valence states), rendering the semiconductor a practical, so-called *extrinsic*, resistive device.^378^ For this purpose, a dopant concentration sufficiently larger than the *intrinsic* semiconductor carrier concentration (*n*_i_) is required (e.g., >1000-fold). Given that *n*_i_ is relatively low, and that a large fraction of *extrinsic* donor-/acceptor-states is usually ionized already at room temperature, doping is achieved at trace-level quantities (e.g., ppm-level atomic fractions). However, we note that the term “doping” is overused in the literature, being employed any time that a foreign element is added in any quantity (up to several wt%), and often even segregating into reactive domains on the surface. As a result, functional MO*_x_* are always either n- or p-type semiconductors, depending on whether *E*_F_ lies closer to the CBM (donor states filled) or VBM (acceptor states filled), resulting in electrons or holes being the majority charge carriers, respectively.

For both n- and p-type MO*_x_*, resistance changes are governed by redox-related electrostatic *surface* fields that shift *E*_F_ relative to vacuum (*E*_vacuum_), i.e., work function (Φ = *E*_vacuum_ – *E*_F_),^379^ thereby inducing a rigid shift of all semiconductor energy levels below *E*_F_, that is, those with positive binding energy. As shown in Figure 4a,b, *at the surface*, this can either bring these states farther from or closer to *E*_F_, depending on whether Φ decreases (“reducing agents”, surface donors, downward band bending) or increases (“oxidizing agents”, surface acceptors, upward band bending), respectively. The spatial extent (*z*_0_) to which such energy-band bendings extend into the material depends on the details of the space-charge layer and further assumptions (e.g., the Schottky approximation used in the “immobile ions” case). Therein, the Debye length (**λ**_D_) is a widely used order-of-magnitude estimate of the electrostatic screening length scale.

**Figure 4:**
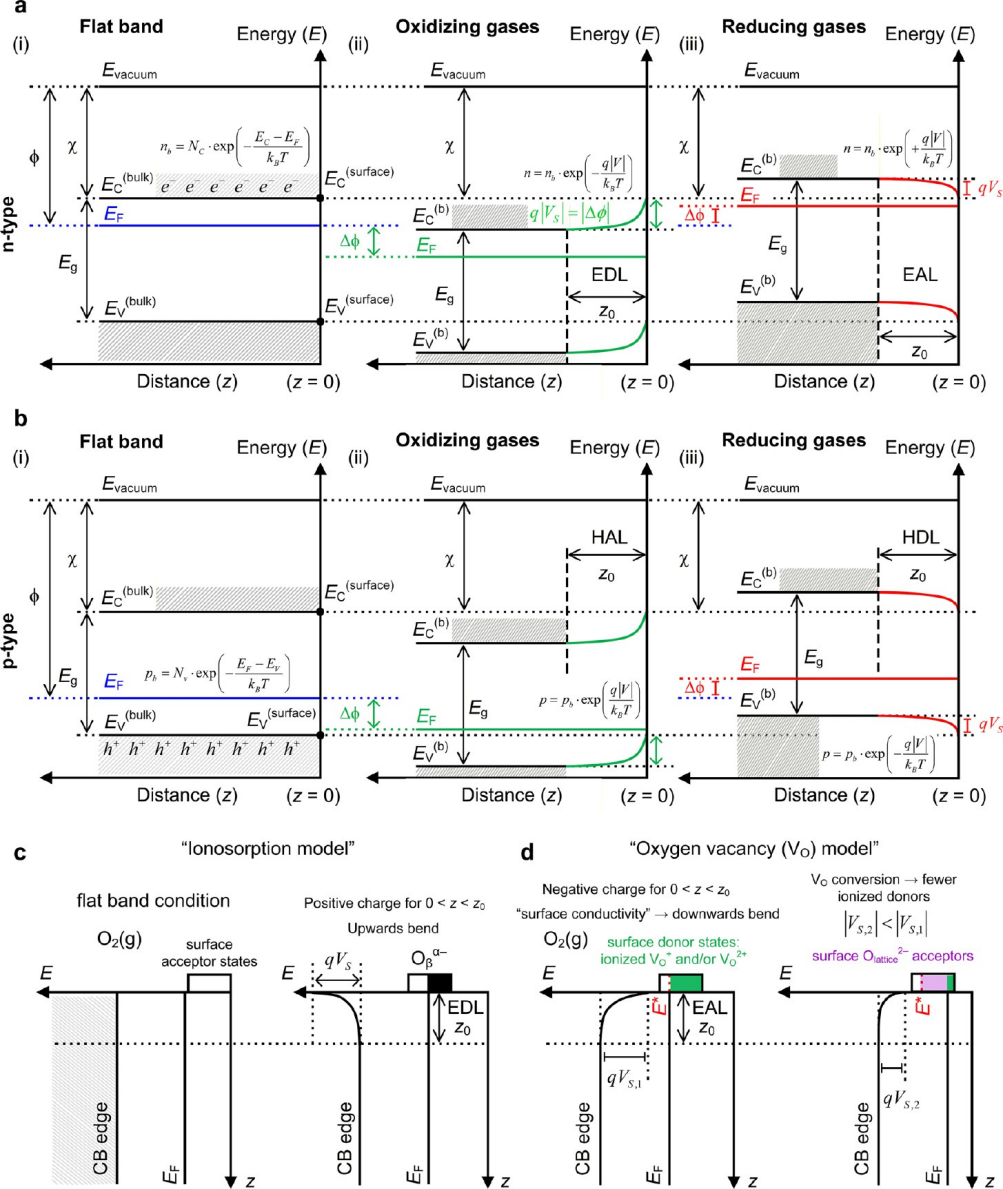
(a) Electronic energy band representation of a (i) pristine n-type semiconductor, along with the effect of (ii) oxidizing and (iii) reducing analytes, leading to the formation of an EDL and EAL, respectively, along with the diagram of a (b) pristine p-type semiconductor forming a HAL and HDL, respectively. EDL: electron depletion layer; EAL: electron accumulation layer; HAL: hole accumulation layer; HDL: hole depletion layer; *z*_0_: space-charge layer thickness. Note that, across the diagrams in (a) and (b), the energy axis has an identical scale, that is, with the same *E*_vacuum_ reference and surface CB/VB edge energies (χ = *constant*). Equilibrium charge-carrier population assuming Boltzmann statistics is provided, explaining the distinct resistance variations of *n*- and *p*-type semiconductors. *N*_C_: effective density of states in the CB; *N*_V_: effective density of states in the VB; *n*_b_: bulk electron concentration; *p*_b_: bulk hole concentration; qV: electrostatic energy profile (Poisson’s equation). Effect of O_2_ adsorption on semiconductor’s energy band structure from the point of view of the (c) “ionosorption model” and (d) “V_O_ model”. Therein, the occupation of surface O_β_^α–^-related acceptor and V_O_-related donor states is schematically illustrated.

Under the hypothesis that the sensing reaction proceeds without formation or consumption of any dipole-like adsorbates, the electron affinity (χ) remains constant, and the magnitude of *E*_F_-/Φ-shift exactly equals that of the upward or downward band bending potential.^380^ Such an assumption, i.e., Δ*χ* = 0, can certainly be debatable even in dry conditions, especially given that many typical reaction intermediates, such as carbonates from CO or hydrocarbons, and nitrates derived from NO*_x_*, may be dipolar (i.e., not ionized donors or acceptors) and remain adsorbed on the surface during sensing.^381^ Nevertheless, this simplification has proved particularly useful for elucidating the transducer function of semiconducting MO*_x_*, for instance, in rationalizing the generally higher sensitivity of n-type compared to p-type materials.^374^ Given the above *microscopic* analogies between *n*- and *p*-MO*_x_* classes, the main distinction is in the phenomenological (i.e., electrophysical) output of surface-potential modulation, owed to distinct space-charge layer configurations.^382^ Upward band bends yield higher and lower resistances for n-type (electron depletion) and p-type (hole accumulation) sensors, respectively; oppositely, downward band bends yield lower and higher resistances for n-type (electron accumulation) and p-type (hole depletion) sensors, respectively. With nanostructured particles of sufficiently small size (*d*), the resistance modulation of semiconductor films can be significant even when sensing extremely low analyte concentrations, largely owing to comparable *d* and λ_D_, meaning that the above modifications in charge-carrier population (i.e., depletion and accumulation) affect a large volume fraction of the material.

As illustrated in Figure 4a,b, such changes in charge-carrier concentrations occur at the surface (*z* = 0) as the result of *E*_F_-shifts relative to the relevant band edge, that is, the CB and VB edges for n- and p-type materials, respectively. In fact, for *n*-type MO*_x_*, upward (CB-)­bending locally *increases* the separation between *E*_F_ and the CB edge, reducing CB-electron concentration in the space-charge layer (electron depletion layer, EDL, higher resistance). For *p*-type MO*_x_*, instead, upward (VB-)­bending locally *decreases* the separation between *E*_F_ and the VB edge, increasing VB-hole concentration (hole accumulation layer, HAL, lower resistance). Opposite-wise occurs with downward bending, providing a microscopic explanation of phenomenological electrophysical measurements, substantiated by simple charge-carrier equilibrium populations in the hypothesis that Boltzmann statistics are valid (see charge-carrier statistics in Figure 4a,b). This assumption typically works in conditions far from the degenerate limit, which can be approached, for instance, by using SnO_2_-based materials to detect relatively high concentrations of H_2_ and/or CO in low-oxygen backgrounds.^383^

Advancing materials research for high-performing air-quality monitors requires bridging phenomenological sensor outputs with an atomistic-level view of the events occurring on the surface. Such insights are usually obtained from *in situ* X-ray absorption and photoemission spectroscopies,^384^ which are now increasingly performed also under *operando* conditions.^385–387^ Traditionally, in the so-called “ionosorption model”,^388^ chemoresponse generation in both n- and p-type MO*_x_* has been attributed to the presence of *extrinsic* reactive oxygen species (O_β_^α–^), which adsorb from molecular O_2_ in the air onto a heated semiconductor (typically at 100–400 °C), filling surface acceptors and trapping electrons from the material until equilibration of the (decreasing) *E*_F_ with the uppermost acceptor-level energy.^389^ Therein, the analyte’s redox chemistry, dominant under oxygen-rich conditions onto heated catalysts, alters the *extrinsic* O_β_^α–^ population by either (i) consuming (iono-)­sorbed oxygens (reducing analytes) or (ii) forming new ones (oxidizing analytes). However, direct experimental evidence for the involvement of O_β_^α–^ species is scarce,^388^ and is complicated by an extremely low amount needed for *E*_F_ equilibration (*<*10^–5^–10^–3^ monolayers, also known as Weisz limitation),^390^ which renders their spectroscopic identification extremely challenging.

Consequently, recent studies have proposed alternative explanations for conductivity modulation that rely on *intrinsic* oxygen species - namely, lattice oxygen ions (O^2–^) of the solid itself. Such participation of *intrinsic* oxygen is on par with the broader heterogeneous catalysis literature, which highlights the mobility and reactivity of lattice O^2–^,^391^ together with the critical role of oxygen vacancies (V_O_) in Mars-van Krevelen-type catalytic oxidations occurring on reducible oxides, like many common sensing materials (e.g., SnO_2_, WO_3_, Co_3_O_4_, etc.). Initially developed for n-type MO*_x_* in the perspective by Blackman,^392^ key to the latter “oxygen vacancy model” is the observation that the electrons localized in surface-V_O_ states are reasonably close to CBM (i.e., “shallow” V_O_ owed to the strong hybridized O­(2p)–metal­(d) character of low-lying CB states), and can be easily thermally ionized at sensor’s operating temperature.

Therein, modulation of surface-V_O_ population shifts *E*_F_ analogously to the *extrinsic* “O_β_^α–^ model”, that is, V_O_-consumption and formation leads to upward and downward band bending, respectively, as experimentally supported by *operando* X-ray photoelectron spectroscopy (XPS) studies on (n-type) SnO_2_,^387^ as well as in-plane Fermi surface mapping from angle-resolved photoemission spectroscopy (ARPES) on high-quality In_2_O_3_ single crystals.^393^ The viability of such a novel “V_O_ model” for p-type materials has likewise been proposed,^394^ with the main distinction being that surface-V_O_-related electron states are “deep” in the bandgap (i.e., close to VBM, owing to the strong O­(2p)–metal­(d) character of upper-VB states). Electrons in these deep V_O_ can recombine with VB holes, again leading to analogous *E*_F_-shifts upon exposure to oxidizing and reducing molecules.

The working principles of “O_β_^α–^ model” and “V_O_ model” are illustrated in the example of an *n*-type MO*_x_* in Figure 4c,d. In the former model, the pristine surface (i.e., prior to O_2_(g) adsorption) is in a flat band condition, assuming no *initial* band bending (Section 3.1.2) and the absence of any M^δ+^ surface trap (Tamm states, Section 3.1.3): (iono-)­sorption of oxygen introduces *extrinsic* species that bend the CB upward. In the “V_O_ model”, instead, inherent V_O_ states act as electron donors, resulting in a downward CB-bending according to the “surface conductivity” picture proposed by Blackman:^392^ dissociative oxygen adsorption consumes some V_O_’s (forming an equivalent amount of O_lattice_^2–^, purple-shaded in Figure 4d) reducing the population of donor states, and diminishing the magnitude of such CB-downward bending. For the sake of completeness, we also indicate the occupation of oxygen-related surface acceptor/donor states that directly pin the position of *E*_F_, affecting these space-charge layers. As mentioned above, the “V_O_ model” also works for *p*-type semiconductors,^394^ the main difference being that surface V_O_ donors (green-shaded in Figure 4d) are energetically closer to the VB edge, thereby directly coupling to the VB-hole density.

Building on this theoretical framework, we now turn to the question that has guided much of the recent literature: how can one enhance the sensitivity and selectivity of chemoresistive sensors? Almost by “default”, there are two widely adopted strategies: (1) the formation of heterojunctions and (2) the surface decoration of pristine MO*_x_* with metal clusters (noble or non-noble in various oxidation states). These approaches can be broadly understood through two complementary mechanisms: *electronic* sensitization,^395^ stemming from band alignment and space-charge redistribution at heterointerfaces, and *chemical* sensitization,^396,397^ which relies on catalytic promotion of surface reactions and charge exchange. While inseparable in practice, we discuss them individually for clarity, emphasizing experimental design principles that can help disentangle their respective roles.

#### 3.1.2 MO*_x_* Heterostructure Formation

As a *thermodynamic* requirement, when two semiconductors are brought into contact, any initial mismatch in their *E*_F_ drives electron redistribution across the interface until *E*_F_ becomes uniform throughout the entire solid.^398^ With a common *E*_vacuum_ reference, electrons flow from the semiconductor with a lower Φ (i.e., higher *E*_F_) to the one with a higher Φ (lower *E*_F_). Since the energy of all electronic states on both sides remains referenced to its initial alignment with *E*_vacuum_ (i.e., assuming no change in χ) this leads to upward band bending on the side with lower initial Φ, and downward band bending on the side with higher initial Φ. The result is the formation of two space-charge regions at the interface, each characterized by its own width and band bending magnitude. This so-called *initial* band bending arises purely from contact-induced charge transfer, without involvement of any surface chemistry or gaseous species.

The implications for sensing are profound: (i) the built-in junction potential largely governs the resistance of heterostructured semiconductors,^399^ providing a powerful means to modulate conductivity responses to air pollutants; and (ii) semiquantitative semiconductor calculations suggest that, in the presence of such *initial* band bending, a given modification of surface charge (*Q*_S_) produces a more pronounced change in electrical resistance.^58^ Figure 5a,b schematically illustrate the formation of a CuO-SnO_2_ p–n junction, which, owing to the lower initial Φ of SnO_2_ relative to CuO, manifests as an electron-depletion layer on the SnO_2_ side and a hole-depletion layer on the CuO side. The report by Wang et al.^213^ underscores these CuO-SnO_2_ heterostructures as highly effective CO sensors operating under mildly humid air (25% RH) and at room temperature, though exhibiting a pronounced response deterioration at higher humidity levels (up to 85% RH).

**Figure 5:**
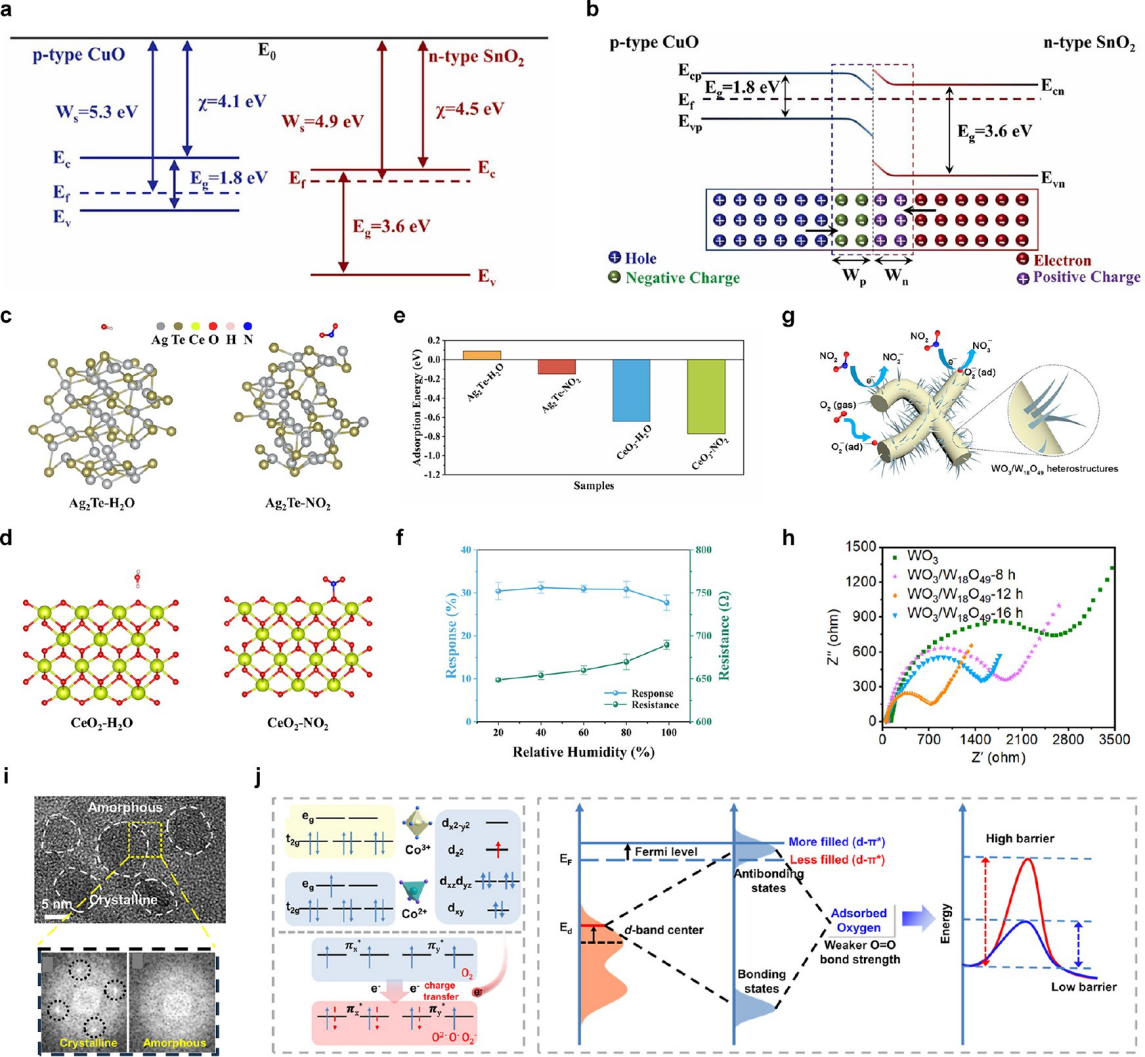
Improving the performance of MO*_x_* sensors by heterostructure formation. Energy-band schematics of a CuO-SnO_2_ heterointerface **(a)** before and **(b)** after E_F_ equilibration, after p–n junction formation. Indicated are the CB (*E*_c_) and VB (*E*_v_) edges, *E*_F_, the electron affinities (χ), work functions (*W*_s_), band gaps (*E*_g_) and the thickness of the space-charge layers on either side of the p–n junction (*W*_n_: electron depletion; *W*_p_: hole depletion). Adapted with permission from ref 213. Copyright 2024 Elsevier. Equilibrium DFT-geometries of H_2_O and NO_2_ molecules adsorbed on (c) Ag_2_Te and (d) CeO_2_, along with their respective (e) adsorption energies (*E*_ads_). (f) Effect of humidity on the sensor response of a 1:2 (by mole ratio) Ag_2_Te/CeO_2_ structure to 1 ppm NO_2_ at 65 °C (left axis), as well as on its baseline resistance (right axis). Reproduced from ref 227. Copyright 2025 American Chemical Society. (g) Schematic of W_18_O_49_ nanorods grown onto WO_3_ nanosheets. The WO_3_/W_18_O_49_ interface controls the conductivity (and its modulation) of the heterostructure, as verified by (h) EIS measurements. Reproduced from ref 230. Copyright 2024 American Chemical Society. (i) TEM image of NaBH_4_-treated (i.e., B-doped) Co_3_O_4_ followed by mild calcination at 225 °C, resulting in the formation of crystalline domains embedded in an amorphous matrix. (j) These abundant and V_O_-rich, amorphous/crystalline interfaces lift the Co d-band center (ε_d_) by ∼0.7 eV compared to purely crystalline B-doped Co_3_O_4_, leading to a lower barrier toward molecular O_2_ activation. Reproduced from ref 296. Copyright 2025 American Chemical Society.

The optimized CuO-SnO_2_ composition (Cu:Sn = 1:4) shows markedly improved response compared to pure SnO_2_ and CuO, with a 5–10-fold increase in sensor response and excellent response-recovery times of 56 and 23 s, respectively. Beyond minor contributions from textural effects (e.g., larger surface areas of the composite compared to pristine MO*_x_*), the authors associate enhanced responsiveness with more favorable CO-adsorption energetics onto the CuO side of the p–n junction. Therein, CO molecules interact primarily with surface Cu atoms: electrons are initially donated to the CuO side but quickly delocalize across the interface into SnO_2_, concurrently narrowing the hole- and electron-depletion widths (*W*_p_ and *W*_p_). Both effects decrease the overall electrical resistance, yielding the observed n-type sensing behavior and superior performance of the composite material. This represents a typical case of *electronic* sensitization, also referred to as “Fermi-level control”,^58^ often described in the literature as the *E*_F_ of SnO_2_ being “pinned” to that of CuO, which “re-routes” the charge accumulated upon CO adsorption across the interface.

In many cases relevant to air-quality monitoring, heterostructuring nanoscaled oxides is an effective strategy to leverage materials with rich redox chemistry that nevertheless would not function properly as standalone MO*_x_* sensors. A notable example is CeO_2_, a reducible n-type oxide with exceptional lattice-oxygen activation properties,^400^ making it a preferred catalyst for numerous selective oxidation reactions, aligning with the recent mechanistic perspectives emphasizing V_O_-mediated sensing (see the “V_O_ model”, Section 3.1.1). However, reports on pure CeO_2_ sensors are scarce, owing to its poor conductivity^401^ and substantiated by its *non*-electronic conduction type (transport of lattice O^2–^ ions). Zhou et al.^227^ fabricated Ag_2_Te/CeO_2_ nanostructures that featured impressively sensitive, selective, and humidity-robust NO*_x_* sensing capabilities at only 65 °C, which the authors integrated into an *Internet of Plants* (IoP) greenhouse environmental monitoring platform. The heterointerface with Ag_2_Te, i.e., a narrower *E*_g_, n-type semiconductor with lower Φ compared to CeO_2_, yields a device with measurable conductivity even at low temperature, dominated by the more conductive Ag_2_Te. Thereby, the strong chemical activity of CeO_2_ toward NO*_x_* molecules is exploited,^402^ which affects the overall resistance only indirectly through subsequent electron redistribution across the interface.

Upon n-n junction formation, electrons are transferred from Ag_2_Te (lower Φ) to CeO_2_ leading to an electron depletion layer on the telluride side (upward bend) and an electron accumulation layer on the oxide side (downward bend). NO*_x_* molecules bind preferentially to CeO_2_ (see also Figure 5c,d), and form surface acceptor states which further increase its Φ, driving *additional* electrons from the telluride into the ceria. In contrast to the CuO-SnO_2_ state-of-the-art CO sensors discussed above,^213^ this electron redistribution increases the space-charge widths, thereby enlarging the dominant electron-depletion layer on the Ag_2_Te-side and producing a significant response. This heterostructuring approach proves particularly powerful, as CeO_2_ not only preferentially binds NO*_x_* molecules but also interacts strongly with H_2_O (Figure 5e), which is ubiquitous in agricultural environments and a common cause of performance deterioration at high humidity. However, because the device resistance is dominated by the Ag_2_Te component (Figure 5f), the baseline remains highly robust against H_2_O interference, and the NO_2_ response is preserved even up to 99% RH. Several heterostructures between MO*_x_* and reduced graphene oxide (rGO) have also been proposed, wherein the bifunctional rGO-MO*_x_* interface similarly fosters device-level performance for air pollutant detection.^403^

In many cases presented in Table 2, system-level enhancements in sensing performance arise from the as-formed Schottky contact, which contributes significantly to the overall device resistance. Although this is a sensible assumption in most MO*_x_* heterostructures of interest for toxic gas detection, direct experimental evidence, typically obtained from electrochemical impedance spectroscopy (EIS)—is usually lacking. In a recent report, Zheng et al.,^230^ showed that the charge-transfer resistance (*R*_ct_) of optimized branched WO_3_/W_18_O_49_ heterostructures (Figure 5g) is substantially lower than that of pristine WO_3_, suggesting a dominant role of the interface between W_18_O_49_ nanorods and WO_3_ nanosheets, as revealed by analysis of the EIS-derived Nyquist plots shown in Figure 5h.

The authors deployed such WO_3_/W_18_O_49_ for NO_2_ detection, achieving exceptionally sensitive, selective, and low-temperature (50 °C) sensing performance. The response was attributed to NO_2_ oxidation by ionosorbed O_2_^–^, forming adsorbed nitrates (NO_3_^–^) as identified by *ex situ* N 1s XPS on NO_2_-exposed WO_3_/W_18_O_49_. This process relies on low-lying (i.e., free) CB electrons supplied by the electron accumulation layer on the W_18_O_49_ side of the n-n junction, wherein pristine W_18_O_49_ has a much higher Φ with respect to stoichiometric WO_3_. Similar to the Ag_2_Te/CeO_2_ system discussed in Figure 5c–f,^227^ the local *E*_F_-downshift on the W_18_O_49_ side drives additional electron withdrawal from the WO_3_ side, further widening both the electron-depletion and -accumulation regions. Because electron-depletion layers exhibit a stronger CB-bending dependence of conductivity than electron-accumulation layers—a key principle of *operando* Φ analysis summarized by Barsan et al.^382^—the overall resistance increases yielding the n-type behavior of WO_3_/W_18_O_49_.

An insightful outcome of heterostructure formation is the opportunity for *electronic structure engineering* of active metal centers. In particular, for transition metal oxides (TMOs) containing cations with partially filled d orbitals, theoretical arguments formulated by Nørskov and co-workers^404^ identify the position of the d-band center (ε_d_) relative to *E*_F_ as a key descriptor governing adsorbate energetics and catalytic trends. Zhao et al.^296^ fabricated B-doped Co_3_O_4_ sensors via NaBH_4_ treatment followed by low-temperature (225 °C) calcination, achieving exceptional sensitivity and selectivity toward acetone at 190 °C, with a detection limit as low as 20 ppb.

Comprehensive structural and spectroscopic analyses revealed that the resulting B-doped Co_3_O_4_ nanoparticles contain several crystalline domains embedded in an amorphous matrix (Figure 5j), characterized by abundant Co^2+^-like species and accompanying V_O_ formation. These features endow the crystalline–amorphous interfaces with a high density of Co sites in tetrahedral (*T*_*d*_) coordination. As shown by DFT calculations and schematically illustrated in Figure 5j, ε_d_ is upshifted by approximately 0.7 eV, approaching *E*_F_. This has a 2-fold effect: (i) it lowers the energy barrier toward O_2_ activation through enhanced d−π* interaction, and (ii) it facilitates acetone adsorption and activation, resulting in a much stronger binding (*E*_ads_ = −3.16 eV) compared to purely crystalline, interface-free B-doped Co_3_O_4_ (*E*_ads_ = −1.83 eV).

#### 3.1.3 Tuning the surface of MO*_x_* sensors

Another broadly applicable strategy to enhance the response of semiconducting MO*_x_*, regardless of composition or conduction type, is surface decoration with catalytically active clusters.^24^ Such secondary phases can profoundly modify surface chemistry either by (i) promoting oxygen activation (increasing, for instance, the concentration of O_β_^α–^ or lattice-oxygen lability, according to the “O_β_^α–^ model” vs “V_O_ model”, see Section 3.1.1), or by (ii) strengthening the binding and activation of analyte molecules. In practice, both contribute to what is often referred to as *chemical sensitization* mechanisms.^397^ When the added component exists as a distinct phase, interfacial equilibration of *E*_F_ and related *initial* band-bending effects are expected whenever electronic communication across the interface is established. These junction-like interactions coexist with catalytic promotion effects, making it experimentally challenging to disentangle purely *chemical* from *electronic* sensitization. To this end, adopting more systematic methodologies from heterogeneous catalysis, such as correlating active-site structure and reaction kinetics (product analysis, activation energies, and reaction orders), could help to bridge the gap between surface chemistry and macroscopic sensor outputs.

As schematically illustrated in Figure 6a, *chemical* sensitization through enhanced formation of O_β_^α–^ species proceeds via a classical “spillover” mechanism.^58^ A representative example is Au-black clusters supported on n-type Ga_2_O_3_,^229^ which exemplifies the cooperative action of both *E*_F_-control and catalytic promotion in toxic-gas detection. Upon formation of the metal–semiconductor (Schottky) junction, the higher Φ of Au-black drives electron transfer from Ga_2_O_3_ to the metal, generating an *initial* electron depletion layer in the oxide. During O_2_ exposure, electrons originating from the Ga_2_O_3_’s CB are captured into oxygen-related acceptor states at the Au surface, until electrostatic equilibrium across the Schottky interface is re-established, thereby increasing the local population of activated O_β_^α–^. These subsequently migrate, or “spill over”, onto the Ga_2_O_3_ surface, where their consumption by analyte molecules induces pronounced modulations of the surface potential and electrical resistance of the supporting oxide.

**Figure 6:**
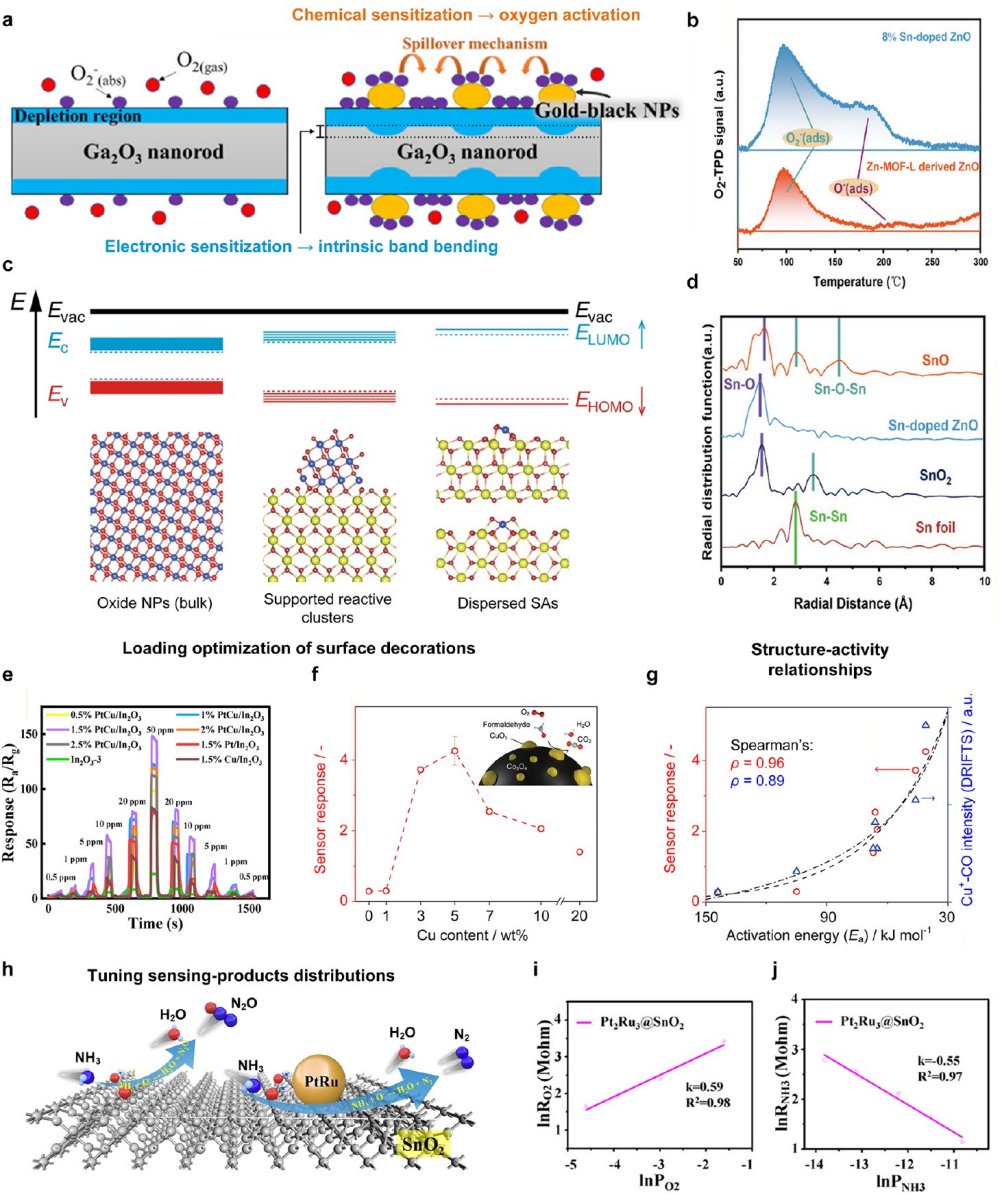
(a) O_β_^α–^ formation mechanism in surface-decorated MO*_x_* via the spillover mechanism. The higher work function of Au-black NPs drives electrons from Ga_2_O_3_, which are available to fill oxygen-related surface acceptor states. The as-formed O_β_^α–^ may spill over to the Ga_2_O_3_ increasing overall oxygen adsorption capabilities and, therefore, sensor signals. Reproduced from ref 229. Copyright 2023 American Chemical Society. (b) O_2_-TPD of pure ZnO and 8 wt % single-atom (SA) Sn-loaded ZnO, showcasing the O_β_^α–^ activation mechanism by the spillover effect. Adapted with permission from ref 307. Copyright 2025 Wiley. (c) From bulk supported nanoparticles (NPs) to single-site surfaces, showing the evolution from continuous band structure to discrete molecular orbitals. Reproduced from ref 400. Available under a CC-BY 4.0 license. Copyright 2020 The authors. (d) Sn-K EXAFS of (Zn-doped-)­SnO_2_ to confirm SA speciation. Adapted with permission from ref 307. Copyright 2025 Wiley. Optimizing the composition of (e) PtCu clusters on In_2_O_3_ and (f) CuO_x_ clusters on Co_3_O_4_ for formaldehyde sensing at low temperatures (<100 °C). The process is rather tedious, motivating systematic studies to bridge (g) active-site structure/speciation with catalytic and sensor performance. Reproduced from ref 311. Copyright 2025 American Chemical Society and Reproduced from ref 318. Available under a CC-BY 4.0 license. Copyright 2023 The authors. (h) Schematic of NH_3_ sensing reaction on the surface of pristine SnO_2_ and Pt_2_Ru_3_-decorated (noble-metal content of 0.5% mole fraction). Note that surface decoration shifts product distribution, largely preventing overoxidation to N_2_O, as also suggested by resistance measurements in (i) O_2_/N_2_ and (j) NH_3_/air, featuring similar fitting exponents in the power-law correlation. Reproduced from ref 260. Copyright 2024 American Chemical Society.

More abundant active O_β_^α–^ species are a key hallmark of *chemical* sensitization, and often probed indirectly through thermochemical methods such as temperature-programmed desorption of O_2_ (O_2_-TPD), as first shown in the seminal work of Yamazoe et al.^405^ Figure 6b compares the O_2_-TPD profiles of pure ZnO and 8 wt % Sn/ZnO nanoparticles,^307^ where the authors attributed the apparent O_β_^α–^ enhancement to a higher O_2_-desorption signal in selected temperature intervals. However, the assignment of these temperature ranges remains largely empirical, and, most importantly, chemisorption-based methods such as O_2_-TPD are inherently nonspecific: they quantify the *total* oxygen released from a MO*_x_* surface rather than the population of truly ionized oxygen acceptors. In principle, the desorbed oxygen signal overwhelmingly reflects neutral chemisorbed species, owing to the fraction of O_β_^α–^, i.e., those effectively involved in band bending, being vanishingly small as constrained by surface electrostatics considerations discussed in Section 3.1.1 (∼ 10^–5^–10^–3^ monolayers according to Weisz limitation).

In the search for new design motifs in chemoresistive sensing, tuning the dispersion state of catalytically active metals has emerged as a powerful strategy.^24^ In particular, single-atom (SA) catalysts^406^ have recently been explored as surface modifiers for MO*_x_* sensors, where the added element is dispersed as isolated atoms anchored onto the oxide lattice. Each SA site coordinates exclusively to lattice oxygen and neighboring cations of the support, achieving nearly 100% metal-atom utilization^204^ and thereby maximizing catalytic efficiency. This enhances molecular activation and turnover, amplifying the overall sensor response through the “catalytic effect”.

Furthermore, SA catalysts (SACs) exhibit distinct and often nonlinear reaction energetics compared with larger clusters, reflecting their discrete orbital interactions and tunable local coordination environments.^407^ SA-decorated sensors also represent a regime where the distinction between *electronic* and *chemical* sensitization becomes increasingly blurred: SAs are direct mediators of analyte adsorption and reaction, yet they modulate the semiconductor’s *E*_F_ differently from the extended *initial* band bending of heterostructures. As schematically illustrated in Figure 6c, the surface atomic site has discrete electronic structures compared with the continuous band structures found in clusters.^400^ Lacking an intrinsic band structure and a continuous interface, SAs cannot sustain a classical space-charge region; instead, they accept or donate charge through localized SA-ligand states, forming Tamm levels that induce nanoscale perturbations in the electronic structure of the supporting oxide.^378^ For instance, in the example of CuO–CeO_2_,^400^ the supporting cation, Ce^4+^, can withdraw electrons from Cu to reduce its ε_d_, which increases the electrophilicity of Cu species. The electron donation from Cu­(3d) to hybridized O­(2p)–Ce­(4f) increases the difference between the highest occupied molecular orbital (HOMO) and the lowest unoccupied molecular orbital (LUMO) of Cu, reaching the maximum splitting at the single-site, free-atom-like limit. This concept is directly applicable to other systems, including single Cu atoms in AgCu single atom alloys (SAAs),^408^ and TiO_2_-supported Cu SACs.^409^

Such SA-systems require thorough assessment of SA electronic and geometric speciation,^410,411^ typically achieved by metal L- and/or K-edge X-ray absorption near-edge structure (XANES) and extended X-ray absorption fine structure (EXAFS) spectroscopies, as illustrated for SA-Sn/ZnO^307^ with Sn-K EXAFS in Figure 6d. The authors attributed superior acetone-sensing capabilities (with a 10-ppm-response > 200, and a lower limit of detection below 1 ppb) to the “catalytic effect” of Sn SAs, which lower DFT-*E*_ads_ compared to pristine ZnO. As discussed above, an additional contribution from *electronic* sensitization cannot be ruled out, likely arising from hybridized Sn-O bonds that localize electrons in Tamm states within or just below the CB of ZnO.

Screening diverse material compositions for chemoresistor optimization remains a tedious yet routine task for sensor researchers. This is illustrated in Figure 6e,f for low-temperature (<100 °C) formaldehyde detection using several wt% of bimetallic PtCu on In_2_O_3_ ^311^ and 0–20 wt % CuO_x_ clusters on Co_3_O_4_,^318^ respectively. To extract useful design principles from such studies toward predictive engineering of chemoresistive materials, it is highly desirable to combine electrical measurements with spectroscopic insight into the active-site structure, obtained, for instance, from (*in situ*) XAS or IR spectroscopy. Coupling these data with oxidation kinetic analysis enables rationalization of sensor behavior and bridges catalyst structure, surface chemistry, and device-level performance, i.e., structure–activity relationships, potentially guiding chemoresistive design across related MO*_x_* families and compositions. In the example of CuO_x_–Co_3_O_4_ shown in Figure 6g, a good degree rank-correlation (Spearman’s) was found between the relative *surface* Cu^+^ amount (i.e., active site structure, obtained from IR of adsorbed CO) and both of the following: (i) oxidation kinetics (lower *E*_a_ for formaldehyde conversion), as well as (ii) formaldehyde chemoresponsive signal, serving as catalytic and sensing activity descriptors, respectively.

Product analysis, on par with reaction kinetics, provides valuable insight into the promotional effects of surface-decorative motifs. In a recent study, Wu et al.^260^ reported an NH_3_ sensor based on SnO_2_-supported bimetallic PtRu nanoparticles, discussing both the *electronic* sensitization that depletes electrons from the SnO_2_ side near the interface and the *chemical* effect of noble-metal decoration—the latter identified as dominant. By *online* FTIR analysis of the reaction products, the bimetallic PtRu sites were shown to suppress N_2_O formation (prevailing for Pt-only decoration) and promote selective oxidation to N_2_, as schematically depicted in Figure 6h.

The synergistic function of Pt and Ru in steering NH_3_ oxidation toward N_2_ is therefore key to enhanced sensing performance. *In situ*-generated N_2_O species can act as surface acceptors, which are neutralized by injected electrons upon NH_3_ exposure. The concurrent presence of oxidizing (N_2_O) and reducing (NH_3_) adsorbates leads to a “competition” between donor and acceptor states, as these have opposite effects on the surface charge balance. Thereby, the resistance modulation is partially compensated, which reduces the overall sensor response, as further supported by resistance measurements in the O_2_/N_2_ and NH_3_/air mixtures shown in Figure 6i,j, respectively. Similar regression coefficients (values of *k*) of the resultant lines in the log–log plots, that is, the values of the exponents in a power-law relation,^412^ suggest that PtRu species convert NH_3_ to N_2_ without overoxidation to N_2_O.

### 3.2 Transition Metal Dichalcogenides (TMDs), Nitrides, and Halide-Based Sensors

Beyond the MO*_x_* sensors reviewed in Section 3.1, many material classes have emerged for air pollutant detection. Transition metal dichalcogenides (TMDs) consist of a combination of transition metals (M) and chalcogen elements (X = S, Se, or Te) in a 1:2 stoichiometric ratio, forming compounds with the general formula MX_2_.^413^ Their two-dimensional (2D) layered structures—similar to MXenes—combined with high carrier mobility, large specific surface area, and rich active sites, make them highly promising candidates for high-performance chemoresistive sensing.^414^

Other materials that have attracted considerable attention are transition metal nitrides (TMNs) and halides (TMHs). TMNs show great potential for sensing applications due to their high chemical stability and electrical conductivity.^415^ Several binary M_x_Ns have been explored for chemoresistive sensing, including GaN, InN, AlN, and different combinations of ternary A_x_M_1‑x_Ns with A, M = In,Ga, and Al.^416^ Transition metal halides (TMHs) include simple metal halides (MH_x_) and metal halide perovskites (MHPs), which feature distinct chemical and physical properties, endowing them with tunable *E*_g_ and optoelectronic characteristics.^195,417^ TMHs also demonstrate exceptional stability under harsh environments, including elevated temperatures and high humidity, especially when integrated into heterojunctions or functionalized with noble metals such as Pd or Pt immobilized on their surfaces,^418–420^ similarly to MO*_x_* as discussed in Sections 3.1.2 and 3.1.3, respectively.

NO*_x_* emissions are the use case of choice for such structures. Zhao et al.^421^ reported the synthesis of binary titanium nitride (TiN_x_) nanoparticles via ammonolysis of the MOF precursor MIL-125. The resulting TiN_x_ nanoparticles were employed as highly selective sensors for NO_2_ detection, even in the presence of potent interfering gases such as NO (selectivity = 30). The experimental and theoretical LLODs were 50 and 2.4 ppb, respectively, satisfying the requirements set by the exposure limits (Table 1), attributed to TiN_x_’s high specific surface area and abundant nitrogen vacancies (V_N_) introduced through the MOF template that highlights the criticality of point defects when metal centers are in a crystallographic arrangement of N^3–^ ligands, similarly to the role of V_O_ in MvK-type oxidation over MO*_x_*-based chemoresistors. The analogy is substantiated by the electronic effect of surface V_N_ that can be thermally ionized under sensor operation, thereby acting as electron donor states directly coupled to the M_x_N’s electronic band structure (see the “V_O_ model” in Section 3.1.1).

A series of highly open-structured (porosity ≥ 84%) TMN films was fabricated by Baut et al.^241^ *via* FSP synthesis of the parent MO*_x_*_,_ followed by its nitridation in NH_3_ at high temperatures. Their dry synthesis–conversion strategy can be flexibly applied to produce porous nitrides of various metals, “on-demand”, as exemplarily shown with Cu_3_N, W_2_N, MoN_x_, TiN, and even TMHs (bromination in HBr) and TMDs (sulfidation in H_2_S), such as CuBr.^45^ and WS_2_ ^242^ for NH_3_ and NO_2_ detection, respectively. Cu_3_N also exhibited remarkable sensing performance toward NO_2_ (1 ppm) operating at a low temperature of 75 °C in 50% RH air, selectively over critical gases (e.g., xylene, toluene, benzene, H_2_, acetone, ethanol, NH_3_, and H_2_S), with, however, modest interference from NO (SI = 3.6). The experimental and theoretical LLODs were 50 and 0.1 ppb, respectively. Further TMH- and TMD-based studies reported NO_2_ sensors, including, for instance, C–MoS_2_ ^239^ that exhibited a significant response (>20) at room temperature, albeit for an exceedingly high 10 ppm NO_2_ (see Table 1 for maximum allowed concentrations), and Nb-doped MoS_2_ ^233^ with a theoretical LLOD below 0.5 ppb.

DFT calculations are increasingly leveraged to bridge sensing mechanisms with theoretical insights. Figure 7a–c shows the selectivity and DFT-equilibrated structures for bismuth oxyselenide (Bi_2_O_2_Se), which belongs to the oxychalcogenide class (M_2_O_2_X). Although Bi_2_O_2_Se is not a classical TMD (MX_2_), it shares some similarities, particularly in its layered structure and electronic properties.^422^ Bi_2_O_2_Se featured the highest response to SO_2_ over (NO, NO_2_, Cl_2_, H_2_, CH_4_, NH_3_, CO, and CO_2_), as shown in Figure 7a, and a LLOD of 20 ppb that is 100 times lower than the recommended threshold of 2000 ppb (Table 1). This was attributed to favorable binding to the Bi_2_O_2_Se surface (*E*_ads_ = −0.76 eV) of SO_2_ on Bi_2_O_2_Se surfaces, compared to other interfering gases such as H_2_ (−0.34 eV), NH_3_ (−0.22 eV), and CH_4_ (−0.21 eV) as illustrated in Figure 7b. In addition, a significant electron transfer (Bader charge calculation) of 2.205|*e*| occurred from the sensor material to SO_2_, on par with the SO_2_-electron withdrawal effect (surface acceptor) and indicative of enhanced adsorption (O-end, Figure 7c) onto surface Se lattice sites of Bi_2_O_2_Se.

**Figure 7:**
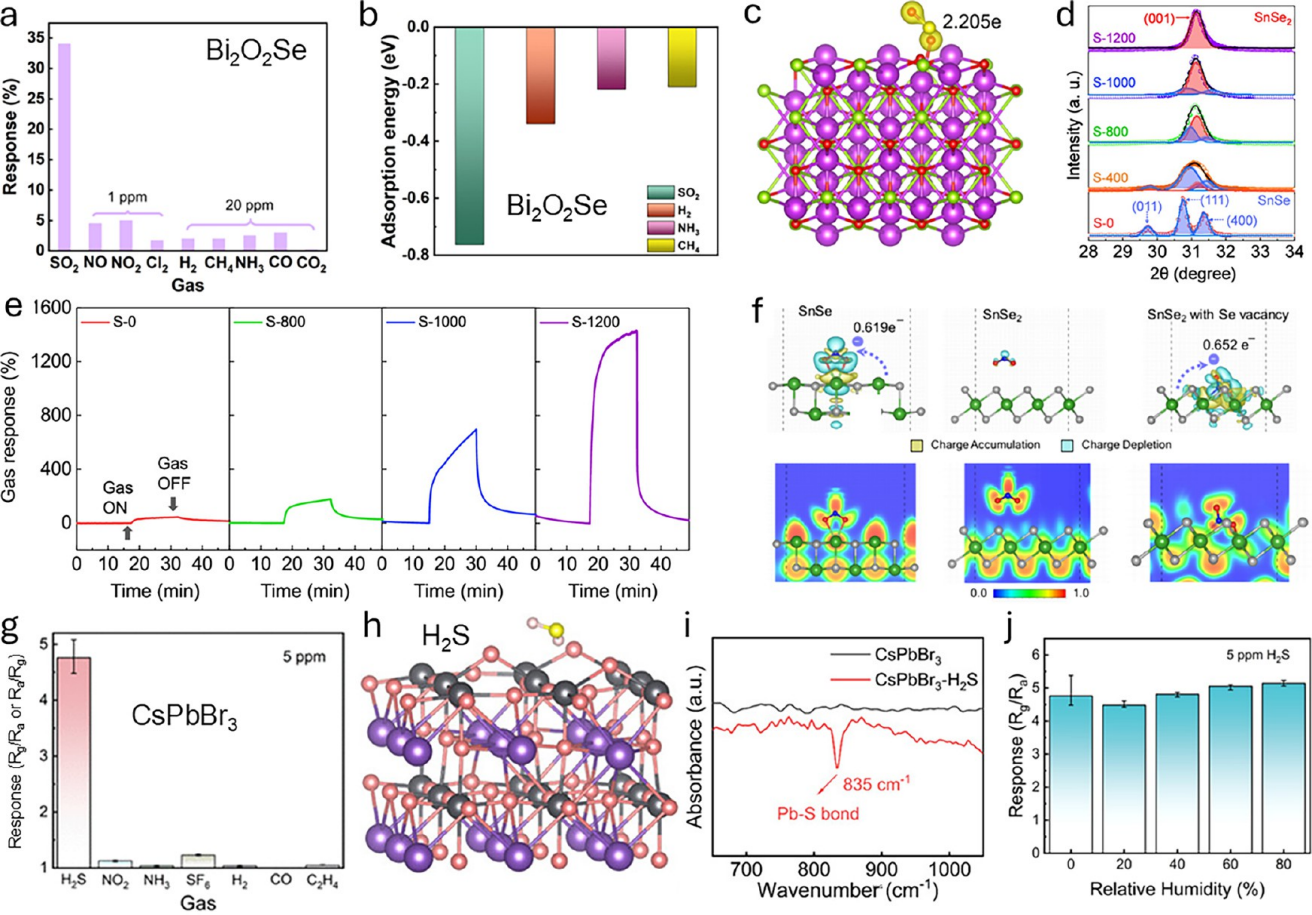
Sensing performance and DFT simulation results of recent works from the past five years that report state-of-the-art sensor materials based on TMDs, TMNs, and TMHs (a) Selectivity analysis of Bi_2_O_2_Se sensor to SO_2_ (1 ppm) and interfering gases such as NO (1 ppm), NO_2_ (1 ppm), Cl_2_ (1 ppm), H_2_ (20 ppm), CH_4_ (20 ppm), NH_3_ (20 ppm), CO (20 ppm), and CO_2_ (20 ppm). (b) The adsorption energy of Bi_2_O_2_Se to SO_2_, H_2_, NH_3_, and CH_4_. (c) The charge density difference and Bader charge of SO_2_ on the Bi_2_Se_2_O adsorption surface. Reproduced from ref 223. Copyright 2025 American Chemical Society. (d) Enlarged XRD diffraction patterns of the main peaks of SnSe*_x_* varying the volume of 1-DDT (0 to 1200 mL). (e) Gas sensing performance of SnSe*_x_* with varying volumes of 1-DDT to NO_2_ (5 ppm). (f) Charge density difference and electron localization function results for NO_2_ adsorption on SnSe, SnSe_2_, and SnSe_2_ with Se vacancy. Reproduced from ref 232. Copyright 2025 American Chemical Society. (g) Responses of the CsPbBr_3_ perovskite-based sensor to H_2_S, NO_2_, SF_6_, H_2_, CO, and C_2_H_4_ (5 ppm). (h) Optimization model for the direct adsorption of H_2_S on the CsPbBr_3_ sensor; (i) FTIR spectra of the Pb–S bonding between CsPbBr_3_ and H_2_S. (j) Humidity robustness analysis based on the response of the CsPbBr_3_ sensor under different relative humidity conditions (0–80% RH) to a 5 ppm H_2_S concentration. Reproduced from ref 332. Copyright 2025 American Chemical Society.

These materials further offer interesting opportunities for phase engineering and investigation of synthesis-phase/structure–activity relationships for air pollutant detection, as notably reported for SnSe*_x_* by Hwa et al.^232^ In their work, different SnSe*_x_* phases were fabricated *via* a hydrothermal method incorporating different volumes (0–1200 mL) of 1-dodecanethiol (1-DDT). Figure 7d shows the XRD patterns of the resulting chalcogenides, showing a gradual evolution from a pure orthorhombic SnSe in the absence of 1-DDT (S-0) to a pure hexagonal SnSe_2_ at sufficiently high 1-DDT content (S-1200), through the formation of mixed SnSe*_x_* under intermediate conditions. Regarding sensing performance, the pure hexagonal SnSe_2_ phase exhibits remarkable behavior for NO_2_ detection at room temperature compared to the orthorhombic SnSe and the mixed SnSe*_x_* phases, as shown in Figure 7e. Specifically, SnSe_2_ demonstrates an outstanding limit of detection of 105 ppt (∼2000 times lower than the recommended long-term limit of 200 ppb) and high selectivity against interferants such as H_2_S, SO_2_, NH_3_, acetone, and H_2_ with SI values of 42, 102, 108, 163, and 186, respectively that was attributed to stronger NO_2_ binding on surface Se vacancies (V_Se_) of SnSe_2_ compared to SnSe (Figure 7f), further reinforcing the defect-chemistry view of chemoresponse generation (i.e., “vacancy model” as discussed previously for V_O_ and V_N_), because the electron(s) at the neutral V_Se_ sites may relax to empty states in the upper-VB (i.e., hole recombination in p-type SnSe*_x_*).

An exemplary perovskite TMH is shown in Figure 7g–j,^332^ wherein the CsPbBr_3_ sensor exhibits outstanding sensitivity and selectivity for detecting H_2_S at room temperature (25 °C). Moreover, the sensor achieves an experimental LLOD below 200 ppb (∼25 times lower than the recommended short-term limit of 5 ppm, Table 1) and remarkable humidity robustness between 0–80% RH (Figure 7h), attributed to more favorable *E*_ads_ of H_2_S compared to H_2_O, which is critical for the application where RH interference is ubiquitous. DFT calculations showed that S-H bond activation was facilitated by S-end adsorption onto Pb sites, resulting in partial oxidation of H_2_S to SO_2_ (Figure 7i), as confirmed by the Pb-S stretches at 835 cm^–1^ during *in situ* IR (Figure 7j).

### 3.3 MXene-Based Sensors

MXenes are a class of two-dimensional (2D) transition metal carbides and nitrides with the general formula M_*n*+1_AX*_n_*.^423,424^ They are derived from MAX phases and are typically synthesized via selective etching methods, producing surfaces with rich chemical diversity and high hydrophilicity owed to several functional groups such as −OH, −O, and −F.^425^ Combined with their large specific surface area and high electronic conductivity, optical, plasmonic, and thermoelectric properties.^426^ MXenes exhibit greater electron mobility, a high density of active sites, and a stronger ability to absorb many toxic air pollutants than most other 2D or conventional materials.^85,427–429^

Despite their rich physicochemical properties, pristine MXenes exhibit limited sensing performance, mainly due to their tendency to self-assemble into stacked nanosheets which reduces the available adsorption sites and the effective electron-transfer ratio of MXene chemoresistive films.^430^ Therefore, MXenes are usually combined with various classes of sensing materials, such as graphene, SMOs, metals, polymers, TMDs, etc., to develop new MXene-based composites capable of enhancing sensing performance, similarly to, for instance, MO*_x_* heterojunctions with chalcogenides (e.g., Ag_2_Te/CeO_2_)^227^ discussed in Section 3.1.2. Over the past five years, most MXene-based composites have comprised MO*_x_*. Notable examples are In_2_O_3_/Ti_3_C_2_T*_x_* ^349^ that detected methanol down to 5 ppm with fast response/recovery times of 6.5/3.5 s, respectively, and SrFeO_3_/Ti_3_C_2_T*_x_* ^304^ with an acetone LLOD below 250 ppb, both operated at room temperature.

In general, Figure 8 provides an overview of some characterizations, *E*_ads_, and sensing performances of selected MXene-based composite materials. A promising sensor for carbon monoxide (CO) detection is the NiCo_2_O_4_/Ti_3_C_2_ composite, which combines spinel-type metal oxide nickel cobalite with MXene. Figure 8a shows the DFT-derived *E*_ads_ of CO calculated for Ti_3_C_2_O_2_ MXene (−0.149 eV), NiCo_2_O_4_ (−0.226 eV), and the NiCo_2_O_4_/Ti_3_C_2_O_2_ MXene heterostructure (−0.399 eV), also reflected by the higher sensor response of the composite (Figure 8b). In addition, the NiCo_2_O_4_/Ti_3_C_2_O_2_ MXene composite exhibits a LLOD of 10 ppm (Figure 8b), fulfilling permissible exposure limits and demonstrating high CO selectivity compared to other common interferants (Figure 8c).

**Figure 8:**
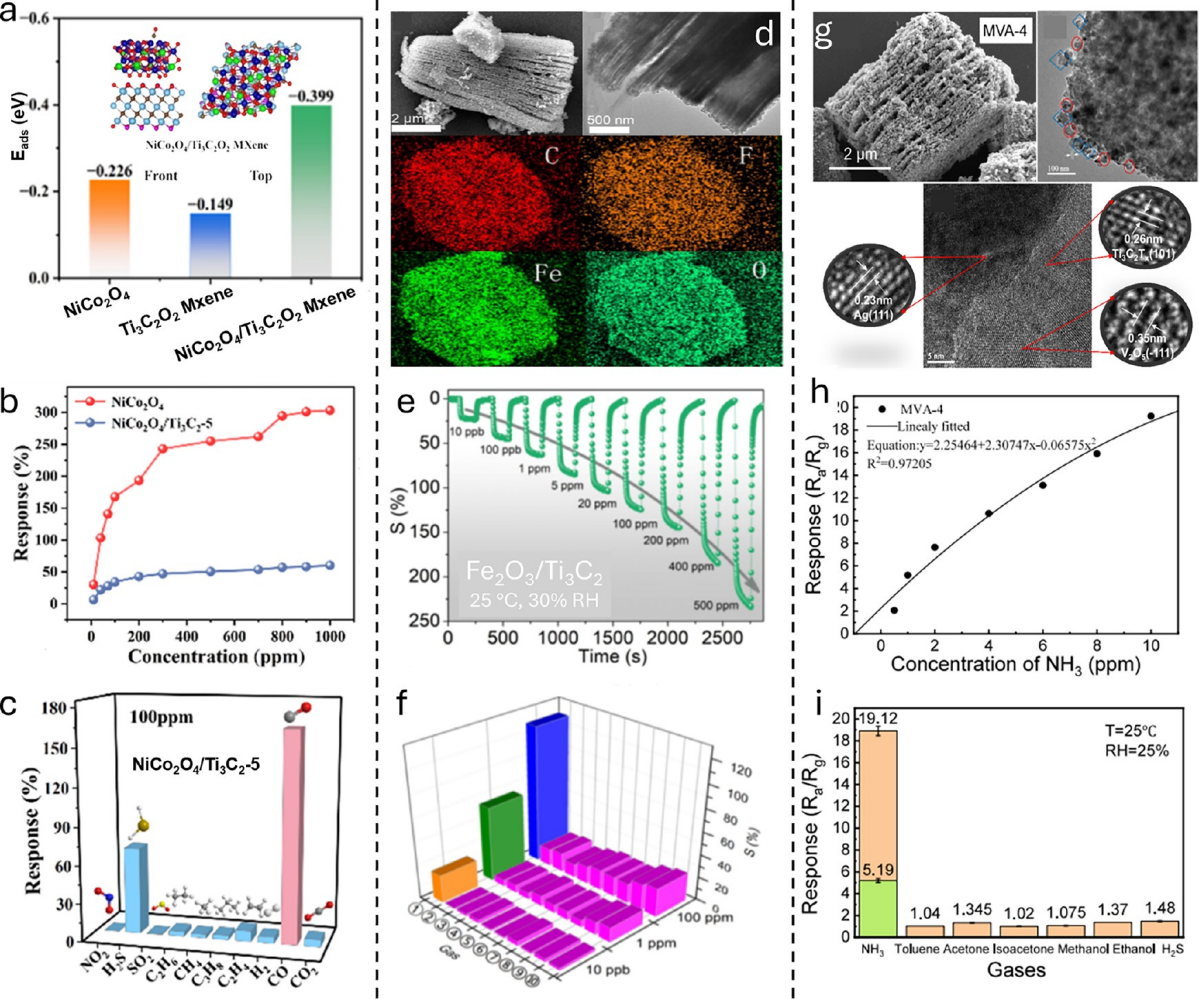
MXene-based composite sensors exhibiting the highest selectivity and lowest LOD values reported in the literature over the past five years. (a) DFT-*E*_ads_ of Ti_3_C_2_O_2_ MXene, NiCo_2_O_4_, and NiCo_2_O_4_/Ti_3_C_2_O_2_ MXene to carbon monoxide (CO). (b) Sensor response of NiCo_2_O_4_ and NiCo_2_O_4_/Ti_3_C_2_-5 wt % sensors upon exposure to 10–1000 ppm CO. (c) Selectivity of the NiCo_2_O_4_/Ti_3_C_2_-5 wt % sensor toward CO detection in the presence of various interfering gases. Reproduced from ref 208. Copyright 2025 American Chemical Society. (d) SEM, TEM, and corresponding EDX mapping images of Fe_2_O_3_/Ti_3_C_2_. (e) Transient response of the Fe_2_O_3_/Ti_3_C_2_ sensor to various H2S concentrations (10 ppb–500 ppm). (f) Selectivity of the Fe_2_O_3_/Ti_3_C_2_ sensor toward different interfering gases: (1) H_2_S, (2) NH_3_, (3) methanol, (4) acetone, (5) ethanol, (6) benzene, (7) CO, (8) NO_2_, (9) CH_4_, and (10) formaldehyde. Reproduced from ref 340. Copyright 2024 American Chemical Society. (g) SEM, TEM, and HRTEM analysis showing the surface morphology of MXene/V_2_O_5_/Ag nanosheets. (h) Sensor response of the MXene/V_2_O_5_/Ag sensor toward various NH_3_ concentrations (0.5–10 ppm). (i) Selectivity and sensitivity of the MXene/V_2_O_5_/Ag sensor toward different interfering gases. Reproduced from ref 259. Copyright 2025 American Chemical Society.

Figure 8d shows the morphology of a Fe_2_O_3_/Ti_3_C_2_ composite, where MXene stacked nanosheets are decorated with active Fe_2_O_3_ nanoparticles forming a uniform Fe_2_O_3_/Ti_3_C_2_ heterointerface. In transmission electron microscopy (TEM), the Ti_3_C_2_ surface exhibited numerous smaller, darker regions, suggesting the formation of the Fe_2_O_3_/Ti_3_C_2_ composite, as confirmed by elemental mapping *via* energy dispersive X-ray (EDX) spectroscopy, which revealed a homogeneous distribution of C, O, F, and Fe. Figure 8e shows the transient response of the Fe_2_O_3_/Ti_3_C_2_ sensor when exposed to different H_2_S concentrations ranging between 0.01–500 ppm. As observed, the sensor exhibits an LLOD below 0.01 ppm, which is 500 times lower than the maximum permissible exposure limit of 5 ppm for H_2_S. Furthermore, the MXene-based composite demonstrates remarkable selectivity toward H_2_S even in the presence of high concentrations of interfering gases (Figure 8f), endowing the Fe_2_O_3_/Ti_3_C_2_ heterojunction with great potential for monitoring indoor and outdoor air quality.

Figure 8g shows the morphological characteristics of yet another heterojunction, MXene/V_2_O_5_/Ag (MVA-4), analyzed by SEM, TEM, and high-resolution TEM (HR-TEM). As observed, the MXene in the MVA-4 composite exhibits an open, accordion-like lamellar structure with nanosheet delamination. Vanadium oxide (V_2_O_5_) and silver (Ag) nanoparticles are uniformly dispersed within the interlayers and on the surface of the MXene nanosheets, showing a high dispersion and minimal agglomeration.

TEM analysis shows that the surface of MXene in MVA-4 is completely covered by irregular polygonal Ag nanoparticles (highlighted in blue) and elliptical V_2_O_5_ nanoparticles (highlighted in red), both uniformly distributed across the material’s surface. The HR-TEM features distinct lattice spacings corresponding to (101), (−111), and (111) planes of the crystallographic MXene, V_2_O_5_, and Ag structures, respectively, in agreement with XRD analysis. The MXene/V_2_O_5_/Ag heterostructure exhibits a high response of ∼20 to 10 ppm NH_3_ at room temperature, with a rapid response time of 8 s and LLOD below 0.5 ppm, ∼50 times lower than exposure regulations—along with outstanding selectivity against critical gases (Figure 8h,i). We conclude that MXene-based materials, when combined with other material classes *via* heterointerface formation (Section 3.1.2) and/or surface functionalization (Section 3.1.3), hold great potential for the development of advanced gas sensors capable of efficiently detecting air pollutants.

### 3.4 MOF-Derived Sensors and Functionalized MOF-Based Sensors

Metal–Organic Frameworks (MOFs) are materials with remarkable potential for gas sensing due to their physicochemical properties, including high porosity, a large specific surface area, 3D-ordered structures, and well-established morphology control, which facilitates the adsorption of gaseous molecules.^431–434^ Pristine MOFs are used as precursors for the synthesis of tunable gas sensors. Still, they can also be directly combined to produce functionalized composite sensors^72,435^ that enhance sensing performance due to the catalytic/filtering effects resulting from MOF incorporation.

The architecture control enabled by MOF-templating is a key factor in developing porous structures with a high surface area, which is crucial for gas-sensing applications. In these applications, the synthesis conditions—e.g., pH, temperature, and solvent—are critical in determining the morphology.^436^ Several MOFs have already been applied in the form of chemoresistive materials’ templating. MOF-5 is a well-known porous cubic precursor for ZnO fabrication, consisting of terephthalic acid as an organic linker and Zn_4_O clusters.^61,437–439^ In contrast, zeolitic-imidazolate frameworks (ZIFs) represent a subclass of MOFs that contain the imidazolate linkers forming metal-linker-metal structures with characteristic *T*_*d*_ metal sites (e.g., Co, Zn, Cd, Cu, Fe).^440,441^ ZIF-67 and ZIF-8 are some examples of Co- and Zn-based MOFs that can be thermally decomposed to obtain Co_3_O_4_ and ZnO structures, respectively.

Over the past five years, the use of MOF-derived and functionalized MOF-based sensors has increased significantly. Zhen et al.^327^ synthesized a Sn-based MOF decorated on TiO_2_ nanotube arrays (NTA), followed by calcination to form an active SnO_2_/SnMOF interface for formaldehyde detection. The authors reported that the SnMOF/SnO_2_@TiO_2_ (annealed at 200 °C) exhibited a high formaldehyde response at room temperature, with fast response/recovery times of 4 and 2.5 s, respectively, albeit to a very high concentration of 6000 ppb. They experimentally quantified down to 750 ppb, which is still ∼10 and ∼100 times higher than, for instance, the WHO guideline (Table 1) and the French recommendation.^442^ The performance shown in Figure 9a,b was attributed to the mild sintering of SnO_2_ nanocrystals upon partial decomposition of the organic linkers, which improved electron transport while retaining the intended SnMOF structure, as confirmed by the homogeneous elemental EDX-map in Figure 9c.

**Figure 9:**
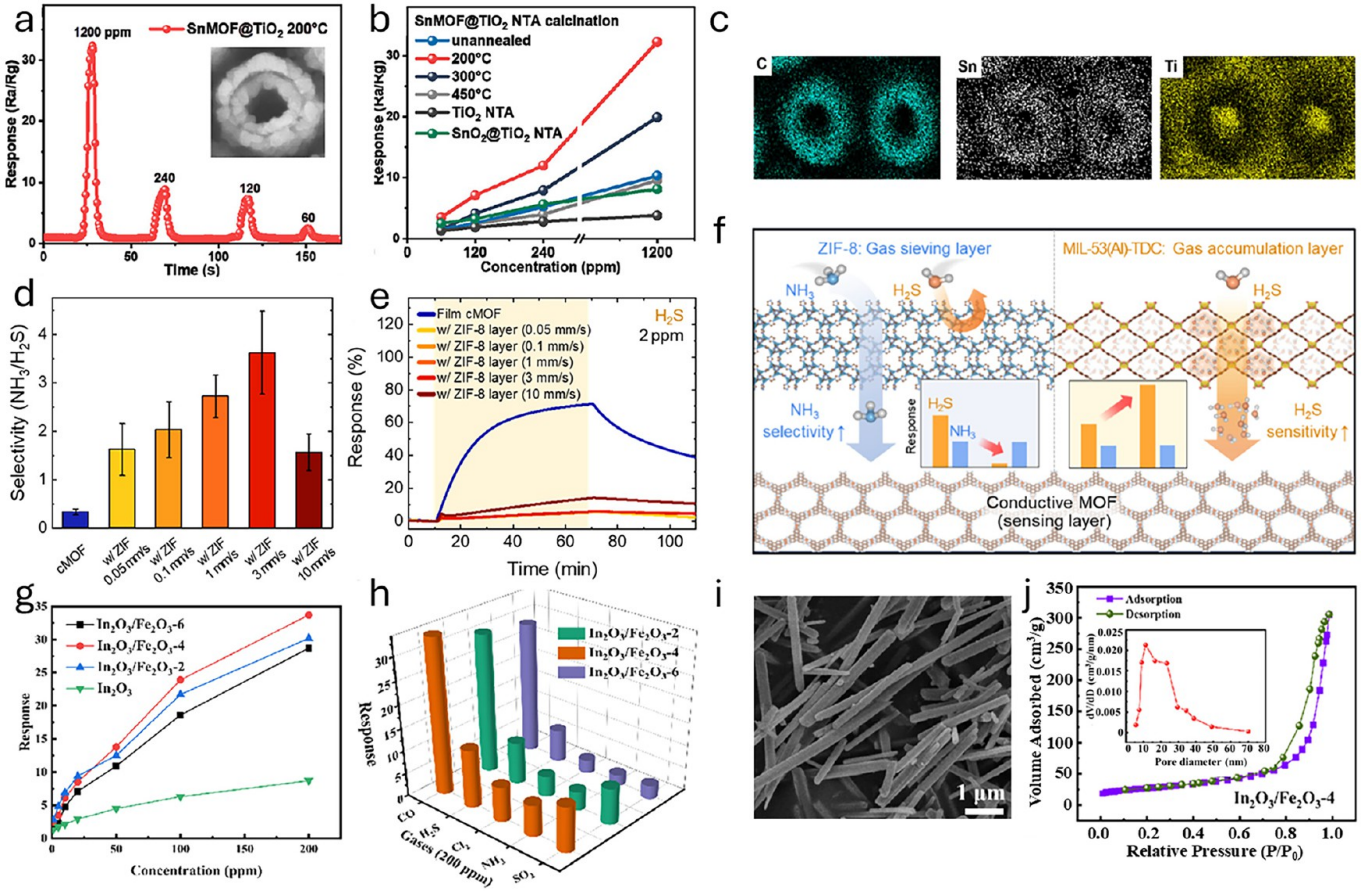
MOF-Derived Sensors and Functionalized MOF-Based Sensors. (a) Response of SnMOF@TiO_2_ after calcination at 200 °C toward different formaldehyde concentrations. (b) Formaldehyde sensing performance comparison between the synthesized materials. (c) EDX mapping of SnMOF@TiO_2_ NTA. Reproduced from ref 327. Copyright 2023 American Chemical Society. Responses toward 2 ppm NH_3_, (d) selectivity of NH_3_/H_2_S, and (e) responses toward 2 ppm H_2_S of the synthesized cMOFs thin films using ZIF-8 layers fabricated at various shearing speeds. (f) Schematic representation of the MOF-on-cMOF thin films mechanisms toward H_2_S and NH_3_ detection. Reproduced from ref 51. Copyright 2025 American Chemical Society. (g) detection performance toward different CO concentrations and (h) responses for the selected interfering gases of the synthesized In_2_O_3_/Fe_2_O_3_-based sensors derived from MOFs. (i) SEM image of In/Fe Bi-MOF. (j) Isotherms of N_2_ and pore size distribution analysis of In_2_O_3_/Fe_2_O_3_-4. Reproduced from ref 443. Copyright 2023 American Chemical Society.

Park et al.^51^ developed MOF-on-cMOF (conductive MOF) thin films using different MOFs and thicknesses controlled by the shearing speed, achieving a wide range of pore architectures that immediately resulted in tunable sensing performance. For instance, they could modify the MOF-on-cMOF response patterns toward NH_3_, H_2_S, and NO_2_ by varying the double-layer preparation using ZIF-8, MIL-53­(Al)-TDC, MIL-53­(Fe), and MFM-300­(Al). Most importantly, the previous approach is very flexible in that the cMOFs’ properties can be modified by choosing MOFs with *ad hoc* pore architecture, gas affinity, metal center, etc, as early demonstrated in Figure 9d,e and conceptually illustrated in Figure 9f as a general concept toward more efficient MOF-on-cMOF air pollutant monitors.

As an example for MOF-templating, Zhao et al.^443^ fabricated a bimetallic organic framework-derived (In/Fe Bi-MOF) In_2_O_3_@Fe_2_O_3_ core@shell nanotubes for CO detection. As shown in Figure 9g,h, the sensor exhibited enhanced performance compared to pure In_2_O_3_, with an approximate 4-fold increase in response to 200 ppm of CO at 260 °C. Beyond the heterojunction effect (M_1_O*_x_*/M_2_O*_y_* interface, as discussed in Section 3.1.2), this is also largely contributed to by improved textural properties, particularly the porous hollow structure, as commonly investigated by SEM (Figure 9i) and N_2_-physisorption (Figure 9j) methods. The latter features distinct isotherm types and hysteresis loops, where sorption branches can be appropriately combined with the Kelvin equation, adsorbate-layer thickness models, and pore-geometry assumption (most often cylindrical, i.e., Barret-Joyner-Halenda)^444^ to infer pore size distributions.^445^ Leveraging more sophisticated experimental protocol (e.g., differential hysteresis scanning) and nonlocal density functional theory (NLDFT) may further enable the determination of pore-network connectivity (i.e., amount of pyramidal, constricted, and occluded mesopores).^446^ Alternatively, other adsorptives than N_2_ (at 77 K) are customarily used, to either achieve higher resolution in the mesopore range (Ar at 87 K),^447^ or to probe the smaller mesopores and down to micropores range (Kr at 87 K),^448^ owed to the quadrupolar moment of N_2_ that can interact with heterogeneous surfaces and compromise accurate mesopore-size discrimination.^445^

### 3.5 Carbon-Based Gas Sensors

Carbon-based materials, mainly in the form of reduced graphene oxide (rGO) interfaced with MO*_x_* phases, have gained prominence in recent years, including in the detection of toxic air pollutants such as NO_2_, CO, H_2_S, and NH_3_.^201,449^ Besides rGO, MO*_x_*’s are also deployed along with carbon nanotubes (CNTs) of different wall thicknesses, i.e., single-walled CNTs (SWCNTs) and multiwalled CNTs (MWCNTs).^450^ As shown in Figure 10a, Haldar et al.^215^ developed an optimized 5 wt % rGO/CuO p-p heterojunction for enhanced low-temperature (25–70 °C) CO_2_ detection, which also displayed notable selectivity against interferants including NH_3_, ethanol, methane, CO, NO_2_, and H_2_S (Figure 10b). This was attributed to the initial Φ-mismatch between the rGO decoration and CuO, which drives electrons from the rGO side to the CuO side, forming a hole-accumulation and hole-depletion layer, respectively (Sections 3.1.1 and 3.1.2). Therein, it is favorable for O_2_ to ionosorb onto the electron-enriched (*p*-)­CuO side, that is, also the main sensor constituent (5 wt % rGO), to restore some of the initial surface hole population (drop in resistance), corroborated by Cu’s inherent oxygen affinity.^400^ As shown in Figure 10c, the authors associate improved performance with the (junction-)­built-in larger O_β_^α–^ concentration, which provides more interaction partners for CO_2_’s sensing reaction—an oxidation to possibly adsorb CO_3(ads)_^2–^ species—although *in situ* spectroscopy was not performed, manifesting as a phenomenological resistance increase.

**Figure 10:**
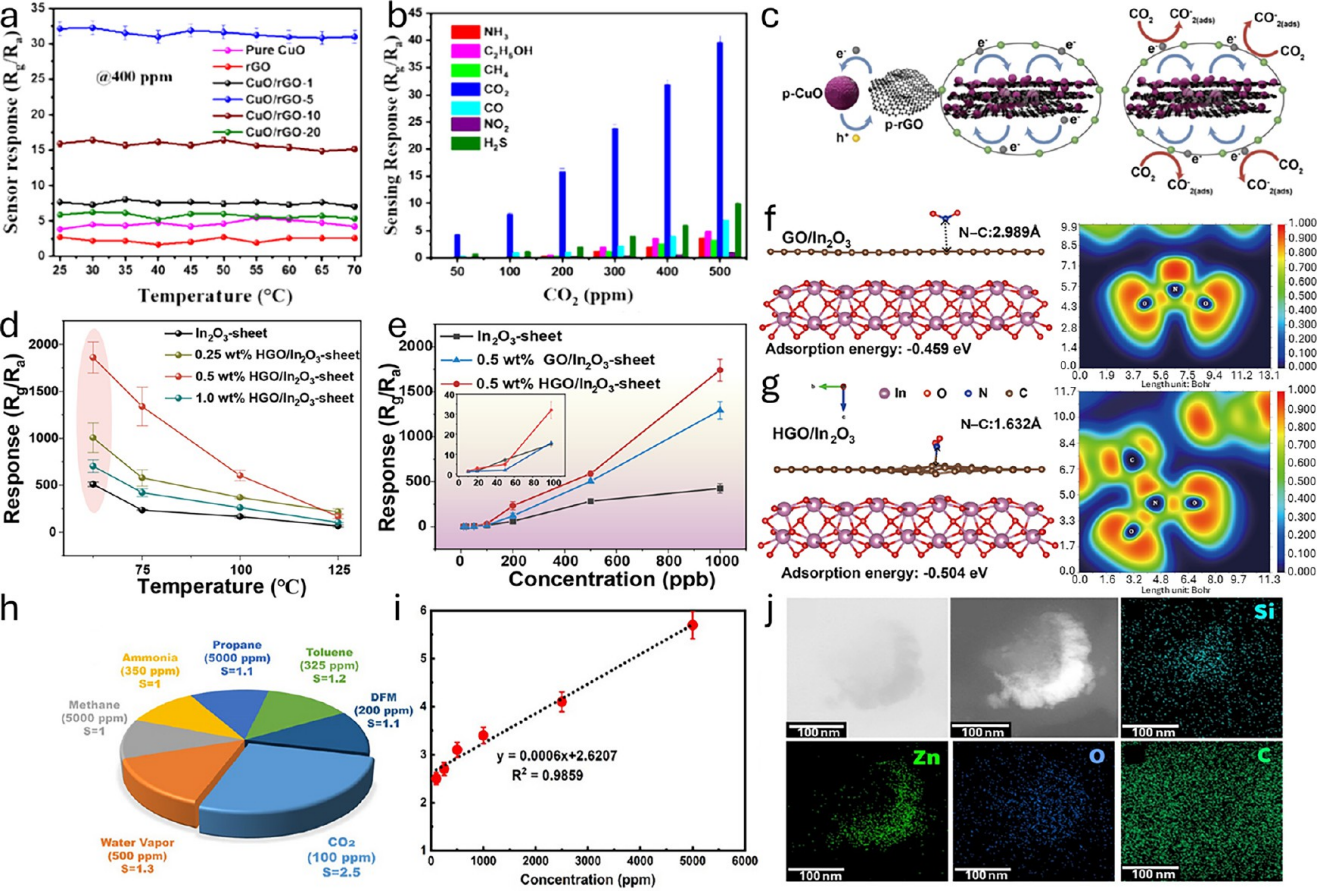
(a) Responses at different operating temperatures to 400 ppm of CO_2_ and (b) Selectivity tests for various gases of the synthesized samples. (c) Scheme of the proposed mechanism for the detection of CO_2_ molecules using the CuO/rGO structure. Reproduced from ref 215. Available under a CC-BY 4.0 license. Copyright 2024 The authors. (d) NO_2_ responses in function of temperature of the synthesized In_2_O_3_-sheet, 0.25 wt % HGO/In_2_O_3_-sheet, 0.5 wt % HGO/In_2_O_3_-sheet, and 1.0 wt % HGO/In_2_O_3_-sheet materials. (e) Sensing performance of In_2_O_3_-sheet, 0.5 wt % GO/In_2_O_3_-sheet, and 0.5 wt % HGO/In_2_O_3_-sheet toward different NO_2_ concentrations. Adsorption simulations and electron localization functions of (f) GO/In_2_O_3_ and (g) HGO/In_2_O_3_, respectively. Reproduced from ref 231. Copyright 2024 American Chemical Society. (h) CO_2_ selectivity tests at 150 °C and (i) responses at different concentrations for the MWCNTs-ZnO sensor. (j) EDX elemental mapping of the MWCNTs-ZnO structure. Reproduced from ref 220. Available under a CC-BY 4.0 license. Copyright 2025 The authors.

Engineering carbon-based loading is an effective strategy to tune the directionality of electron flow in C/MO*_x_* composites. For instance, *p*-type holey graphene oxide (HGO) exhibits a lower Φ (4.64 eV) than that of rGO (5.20 eV). Consequently, 0.5 wt % HGO/In_2_O_3_ ^231^ forms a heterojunction in which electrons flow from *n*-type In_2_O_3_ (Φ = 4.53 eV) toward HGO, creating an electron-depletion region in In_2_O_3_ and a corresponding hole-depletion region in HGO, that is, an *initial* band bending configuration. As discussed in Section 3.1.2, NO_2_ adsorption and the resulting accumulation of negative surface charge (due to electron withdrawal from the In_2_O_3_ CB) further enhance the NO_2_ chemoresponsivity. The authors reported exceptionally high responses (>1800) at 60 °C for 1000 ppb NO_2_ (Figure 10d) and achieved quantification down to guideline-relevant levels, e.g., a response > 4 at 10 ppb NO_2_ (Figure 10e).

The DFT analysis (Figure 10f,g) revealed that HGO loading optimizes the NO_2_ adsorption energetics (*E*_ads_ = −0.504 eV vs −0.459 eV for GO), favoring stronger substrate–adsorbate charge transfer and tighter N-C bonding, as evident from the electron localization function (ELF) contours. Although rarely employed in the sensing literature, bond-contact analysis—particularly the integration of the crystal orbital Hamilton population (COHP) up to *E*_F_, yielding the integrated COHP (ICOHP)^451^—is a valuable computational descriptor of total bond strength that enables the investigation of bond activation and formation mechanisms.^452^ Aleksanyan et al.^220^ fabricated a MWCNTs-functionalized ZnO that was additionally decorated with Pd catalytic nanoparticles for CO_2_ detection. The sensor was moderately robust to higher-concentration interferents (Figure 10h) and displayed the air-quality-relevant CO_2_ dynamic range (Figure 10i), which was attributed to the cofunctionalization with Pd and MWCNTs that were uniformly distributed on the nanostructured support (Figure 10j).

### 3.6 Polymer and Organic Material-Based Sensors

Conducting polymer and organic materials have gained increasing attention in recent years, mainly for gas sensing applications, including environmental monitoring and air quality assessment.^453,454^ Conducting polymers and organic-based nanostructured sensors can detect a wide range of species, including heavy metal ions, explosive materials, toxic gases such as CO, SO_2_, NO_2_, H_2_S, and NH_3_, as well VOCs, water pollutants, and others—mostly at room temperature.^454,455^ In this context, Figure 11 and Table 2 present some state-of-the-art gas sensors based on conducting polymers and organic materials developed over the past five years for the detection of toxic gases such as SO_2_ and NH_3_.

**Figure 11:**
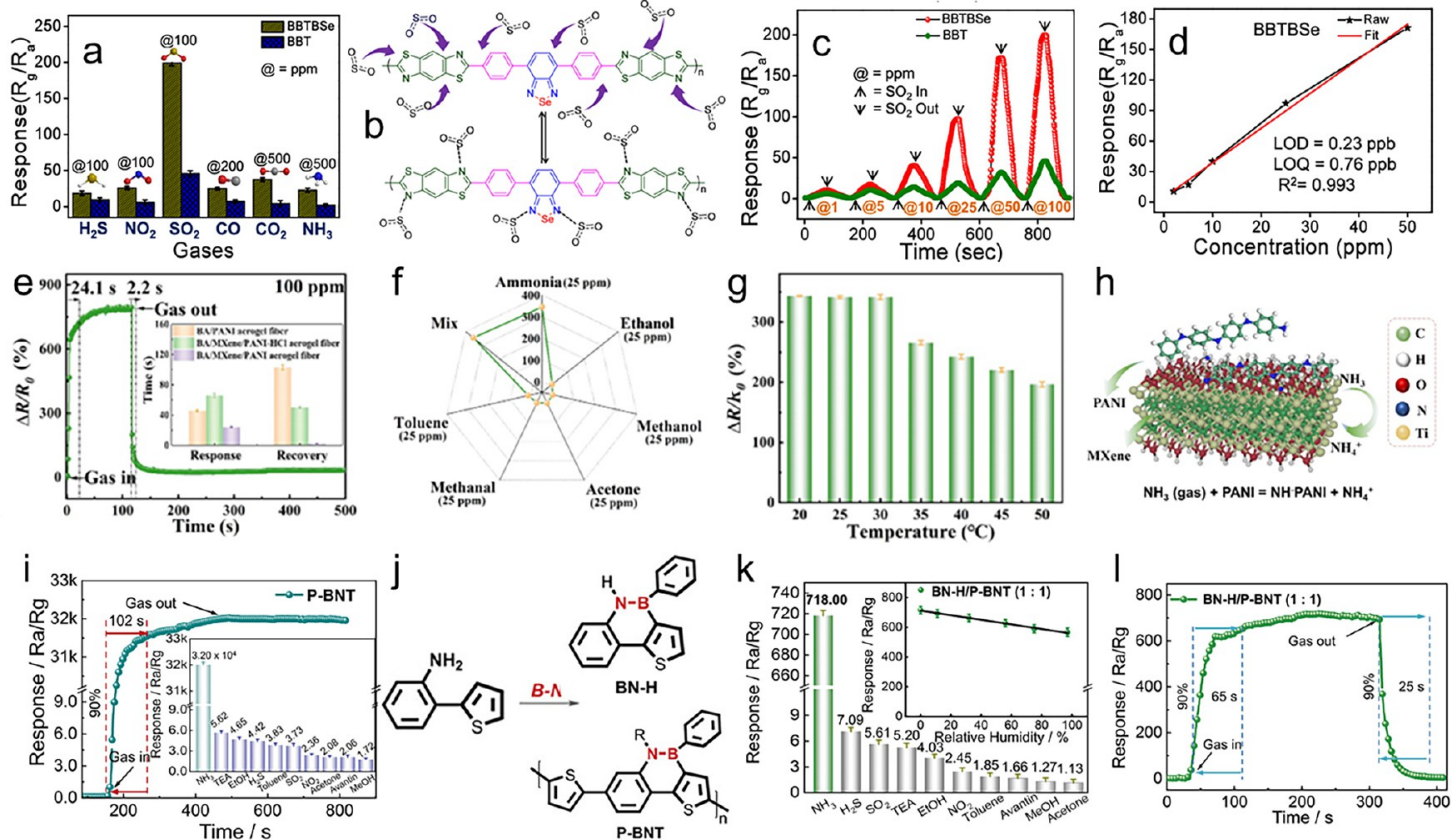
Conducting polymers and organic composite materials chemiresistive gas sensors exhibiting the best sensing performance reported in the literature over the past five years. (a) Sensitivity and Selectivity of BBTBSe and BBT sensors for H_2_S, NO_2_, SO_2_, CO, CO_2_, and NH_3_. (b) Scheme of interaction between BBTBSe and SO_2_ Molecules. (c) Dynamic response and transient behavior for BBTBSe and BBT sensors for SO_2_ in different concentrations (1–100 ppm). (d) Fitting linear relation between SO_2_ concentration (1–50 ppm) and the response of the BBTBSe sensor. Reproduced from ref 224. Copyright 2024 American Chemical Society. (e) Response and recovery curve of the BA/MXene/PANI aerogel fiber at the NH_3_ (100 ppm) at 20 °C. (Inset is a comparison graph of response/recovery time between the BA/PANI aerogel fiber, the BA/MXene/PANI-HCl aerogel fiber, and the BA/MXene/PANI aerogel fiber). (f) Selectivity of the BA/MXene/PANI sensor for NH_3_, ethanol, methanol, acetone, methanal, toluene, and sensitivity of a mix with all gases (the mixed gas is composed of 25 ppm of NH_3_ and 100 ppm of other toxic gases). (g) Sensing response of the BA/MXene/PANI at temperatures between 20–50 °C to detect NH_3_ (25 ppm). (h) Schematic sensing mechanism of MXene/PANI to detect NH_3_. Reproduced from ref 256. Copyright 2025 American Chemical Society (i) Response-recovery curve of P-BNT - Inset: Selectivity of P-BNT to NH_3_, TEA, EtOH, H_2_S, Toluene, SO_2_, NO_2_, Acetone, Avantin, and MeOH (40 ppm). (j) Organic gas sensing material with conventional building blocks based on B-N units. (k) Response-recovery curve of BN-H/P-BNT. (l) Selectivity analysis of the BN-H/P-BNT sensor to the same testing gases at 40 ppm and sensor response in different humidity conditions (0–100% RH). Reproduced from ref 263. Available under a CC BY 4.0 license. Copyright 2024 The authors.

The conducting polymer BBTBSe, which combines a thiazole-decorated conjugated polymer (BBT) with a benzo­[2,1,3]­selenadiazole ring (BSe), was utilized as a room-temperature sensor for detecting SO_2_ selectively, as shown in Figures 11a–d.^224^ Therein, the combination of BBT and BSe significantly enhanced the SO_2_ sensing performance (100 ppm) and exhibited a response value (199.4) that was 4.3 times higher than that of the BBT sensor (45.7). Moreover, the BBTBSe sensor demonstrated rapid response-recovery times of 60 and 70 s, respectively. The BBTBSe sensor exhibits selective detection of SO_2_ compared to other interfering toxic gases such as H_2_S (100 ppm), NO_2_ (100 ppm), CO (200 ppm), CO_2_ (500 ppm), and NH_3_ (500 ppm) (Figure 11a). The authors attributed improved sensitivity and selectivity toward SO_2_ to the higher number of N atoms in the aromatic rings of the BBTBSe structure compared to BBT (Figure 11b), resulting in enhanced delocalization of electron density from N to electron-deficient S during SO_2_ reception. As a result, electron density is withdrawn from neighboring C = N, which disrupts the π resonance of the conjugated polymer rings, producing an *n*-type response (electrical resistance increases) with rather fast response/recovery dynamics between 1–100 ppm SO_2_ at 25 °C as shown in Figure 11c. BBTBSe chemoresponsive signal is higher than BBT (11.3 vs 6.3 at 1 ppm of SO_2_), and exhibits an experimental and theoretical LLOD of 0.76 and 0.23 ppb, respectively (Figure 11d), i.e., ∼9000 times lower than the guideline (2000 ppb).

PANI materials are considered promising sensing candidates due to their high electrical conductivity, good reversibility, ease of fabrication, low cost, and excellent environmental stability.^456^ Figure 11e–h shows a BA/MXene/PANI aerogel fiber-based sensor for selective NH_3_ detection,^256^ exhibiting an outstanding sensing performance with high responsiveness of 807% and fast response/recovery times of 24.1 s/2.2 s, respectively (Figure 11e), with a LLOD of 1 ppb that is 25000 times lower than exposure guidelines. In addition, the sensor demonstrates excellent selectivity toward NH_3_ against critical gases (Figure 11f), and advantageous robustness to higher concentration interferants and even in gas mixtures.

Increasing the temperature between 20–50 °C (Figure 11g), NH_3_ response decreases from 341% to 196%, attributed to lower NH_3_ adsorption on the semiconductor polymer composite at higher temperature. When the sensor is exposed to high-humidity conditions, it shows only a slight variation in response at 90% RH, associated with the dissociation of water molecules and fewer sites available for NH_3_ adsorption. The polymer is a mixed electronic-ionic semiconductor, and the reception mechanism behind NH_3_ detection at the MXene/PANI surface is based on the deprotonation and protonation of PANI during adsorption and desorption, respectively (Figure 11h). Upon NH_3_ exposure, protons are captured from PANI to form NH_4_^+^ ions, leading to a concurrent decrease in both hole- and H^+^-density. The change in *E*_F_ yields measurable electrophysical signals that are amplified through the p–n heterointerface, with a built-in EDL and HDL on MXene (lower Φ) and PANI (higher Φ) sides, respectively.

Another conducting polymer is P-BNT, which is based on a novel π-conjugated poly­(triarylboron–boron–nitride–phenylene) characterized by B–N bonds, fabricated by Wang et al.^263^ (Figure 11i–l). It is one of many significant contributions to the field of organic solar cells^457^ and high-performance to NH_3_ detection, similar to other conducting polymer materials reported in the literature.^458^ This material exhibits an outstanding sensor response of 32’000 to 40 ppm NH_3_ that is 3000 times higher than the monomer BN-H (Figure 11i and j), along with exceptional selectivity (>5 × 10^3^) and a fast response time of 102 s, with, however, long recoveries due to strong analyte adsorption possibly limiting sensor’s reusability. This was overcome by combining small monomer BN-H with P-BNT (1:1) to produce a BN-H/P-BNT heterostructure. Despite having a lower NH_3_ sensitivity, the hybrid sensor exhibits much faster transients with response/recovery times of 65/25 s (Figure 11k) and preserved a very good selectivity (>100), with proven performance under fully H_2_O-saturated environments (Figure 11l) and a theoretical LLOD of 13 ppb. Therein, the authors suggested that the monomer BN-H induces interfacial effects that suppress strong acid–base interactions promoting NH_3_ oxidation, while enhancing the electronic properties of the sensing layer.^263^

## 4 From Chemoresistive Materials to Functional Devices

The widespread use of air quality sensors in everyday applications is still limited, mainly because current devices lack sufficient selectivity to quantify trace pollutant concentrations in complex indoor or outdoor air that contain hundreds of potential interferants (e.g., >250 VOCs in typical indoor air^459^). Here, we highlight strategies to bridge the critical gap between (a) identifying promising sensing materials or chemistries (Section 3) and (b) turning them into devices that can operate reliably in real-world settings. Before doing so, we briefly review the engineering challenges of implementing semiconductor gas sensors in compact, low-power formats and how MEMS-type microhot-plate (MHP) technology makes their power consumption compatible with battery-operated and portable systems.

### 4.1 Sensing Substrates and Film Deposition

We give a general overview of how modern MHP gas sensors are built and the criticality of their mechanical and thermal design. In Figure 12a, a typical MHP is shown with a small silicon island suspended on a thin dielectric membrane obtained by etching. An embedded PMOS transistor serves as a heater and a polysilicon resistor acts as a temperature sensor for the control loop. A nanocrystalline semiconductor film on metallic (commonly Pt or Au) interdigitated electrodes (IDEs) serves as the sensing layer.^460^ Thanks to the low thermal mass of the membrane and its suspension, the power needed to reach a few hundred degrees °C is in the order of some tens of mW, whereas the surrounding chip with its readout electronics remains unheated. Figure 12b shows the cross-section of such a MHP coated with a porous, flame-aerosol-deposited MO*_x_* layer obtained by focused ion beam SEM.^243^ Precise deposition of the sensing layer exclusively on the interdigitated electrode area is critical and has been accomplished by shadow masks or photoresist windows so that drop-casting, sputtering, or aerosol deposition only coat the IDE zone, and not the bond pads or cold support frame.

**Figure 12:**
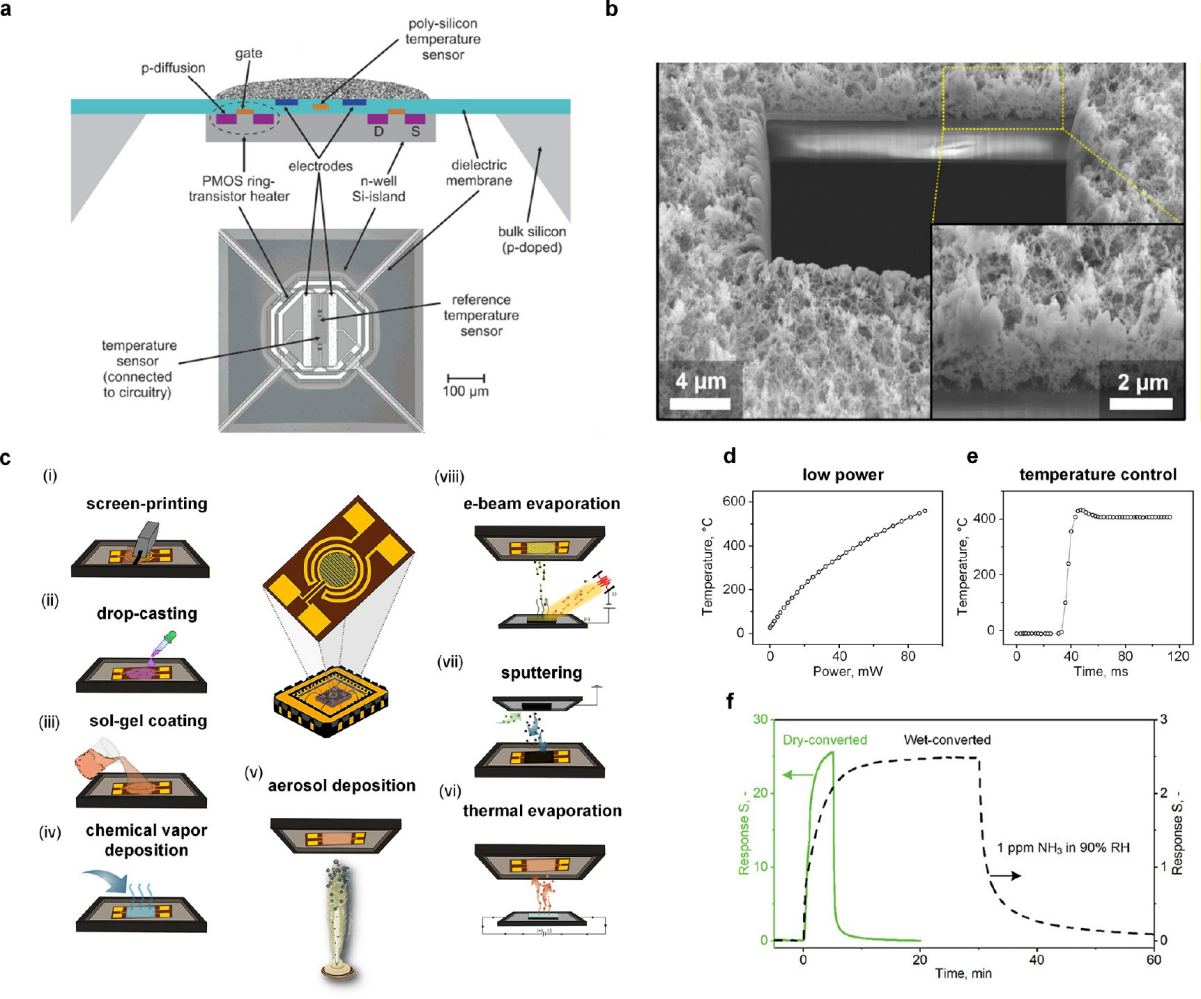
(a) Schematics of a MEMS-type MHP: a small silicon island on a thin dielectric membrane is heated by an integrated MOS transistor, as monitored by a polysilicon temperature sensor, and contacted by interdigitated electrodes that support a nanocrystalline semiconductor film. Reproduced from ref 460. Copyright 2006 American Chemical Society. (b) Cross-section SEM image of MHP-supported MO*_x_* film after cutting a square with a focused ion beam; the empty gap beneath the membrane provides strong thermal insulation, while careful mask-design and alignment ensure that the film covers the electrode area. Reproduced from ref 461. Available under a CC-BY 4.0 license. Copyright 2020 The authors. (c) Illustration of representative film-deposition techniques, including solution-based methods, physical vapor deposition, chemical vapor deposition, and aerosol-based routes. (d, e) Exemplary power-temperature characteristics and fast thermal transients achievable with suspended MHPs. Adapted with permission from ref 462. Copyright 2018 The authors. (f) Impact of film architecture (porous vs compact) on sensing performance. Reproduced from ref^45^. Available under a CC-BY 4.0 license. Copyright 2020 The authors.

From a historical perspective, two types of MHP have been extensively studied. In so-called *closed-type* membrane MHPs, the thin diaphragm spans the entire cavity. According to finite element analysis calculations,^463^ this design is mechanically robust and relatively easy to fabricate, at the expense of broad heat-induced dome-shaped deflection, significant mechanical stress at the clamped edges, and higher heat transfer into the substrate that leads to larger power consumption.^464^ On the other hand, in a *suspended-type* membrane design, only a small central platform is left and held by narrow beams that strongly reduce heat transfer, yielding lower power consumption, and the hot zone is sharply confined to the sensing area. However, stress and bending induced by thermal deflection are concentrated in the beams, so careful engineering is required to avoid failure. Moving from closed to suspended membranes has been a key achievement in the microfabrication of gas sensors to reduce power consumption from hundreds to a few tens of mW at typical operational temperatures, while also keeping the embedded electronics for signal conditioning safely below their maximum operating temperatures.

A key step is the chemoresistive film preparation (Figure 12c), a demanding task especially in the small area of interdigitated electrodes of suspended-membrane MHPs. Therein, a reliable, consistent, reproducible preparation of the sensing layer is key and essential also for thicker ceramics-based chips used in “fundamental” sensor research with the aim to obtain mechanistic insights and further tailor materials design, as discussed throughout Section 3. Screen-and inkjet printing of suspensions containing semiconductive nanoparticles are robust and probably most established for the deposition of sensing films on MHPs. Thereby, the formulation of stable and functional suspensions is particularly challenging to avoid, for instance, in the case of inkjet printing, the formation of satellite droplets or splashing under operational conditions.^465,466^ Also frequently used are drop-casting of suspensions or sol–gels that can be confined to the interdigitated electrode area, but film uniformity can be compromised by the coffee-ring effect.^467^ Sputtering and thermal or *e*-beam evaporation provide dense or only moderately porous thin films with excellent thickness control. Chemical vapor deposited films can be grown very conformally, also on complex MEMS topographies. Aerosol deposition allows direct deposition of very porous nanoparticle films by thermophoresis^468^ or denser films by impaction^469^ with variable film adhesion strength to the membrane.

As shown in Figure 12d,e, a suspended-membrane MHP can reach 400 °C with less than 60 mW, and its temperature can be achieved within ms.^462^ Such fast and efficient heating makes it easy to run temperature-cycling schemes,^470,471^ where the sensor is periodically heated and cooled to extract richer response patterns and to speed up gas adsorption and desorption. Furthermore, the film morphology can critically affect sensor performance. In a previous study, a dry bromination of an aerosol-deposited CuO film to CuBr yielded much higher porosity than wet-bromination (43 vs 78%).^45^ As shown in Figure 12f, the highly porous CuBr sensor showed a larger and faster response to the air pollutant NH_3_ (Table 1) in humid air, attributed to its open structure facilitating fast mass transfer within the mesoporous film. As a result, deposition methods that promote porous or hierarchical architectures can boost performance; yet, their interplay with the mechanical and thermal constraints of MEMS MHPs remains only partially explored.

### 4.2 Toward Sensor Systems: Strategies to Enhance the Selectivity

The critical challenge for semiconductor gas sensors remains their often insufficient chemical specificity, especially when analyzing real-world gas mixtures with many potential interferants. Once the MHP platform is in place, selectivity can be further enhanced by combining several, differently selective sensors to arrays (so-called electronic noses)^472,473^ that have, for instance, led to promising discrimination in wearable sensor devices.^474,475^ Processing their signals, in some cases with integrated ML–assisted methods,^476^ enables enhanced selectivity^477^ and/or simultaneous multitracer detection,^478,479^ concepts that have been reviewed recently elsewhere.^109,480^ Another simple but powerful approach is to precondition the gas mixture before it reaches the sensor. Filters before and overlayers on top of the sensing film alter the analyte matrix by exploiting (1) differences in physi/chemisorption strength or (2) chemical reactivity. In this section, we focus on sorption^481^ and catalytic filters,^67^ which have shown particular promise for turning laboratory sensor concepts into deployable devices.

In a sorption filter, a packed-bed of adsorbent particles (for example, Tenax TA, a hydrophobic polymer)^482^ acts as a miniaturized gas chromatography (GC) column placed upstream of an otherwise nonspecific sensor. As shown in Figure 13a, in the simplest linear-chromatography description, and neglecting axial dispersion and mass transfer, the inverse analyte speed, *dt*/*dz*, scales with a combination of bed-void fraction (ε), analyte’s thermodynamic adsorption strength (*H*_i_), and the inverse interstitial velocity (*u*).^483^ Consequently, not only the choice of sorbent but also filter-assembly parameters such as packing density, diameter, and length must be tuned to obtain the desired separation (Figure 13b)—a design space that is, of course, well covered by high-performance GC manufacturers but still needs more attention in the context of low-cost sensor systems.

**Figure 13:**
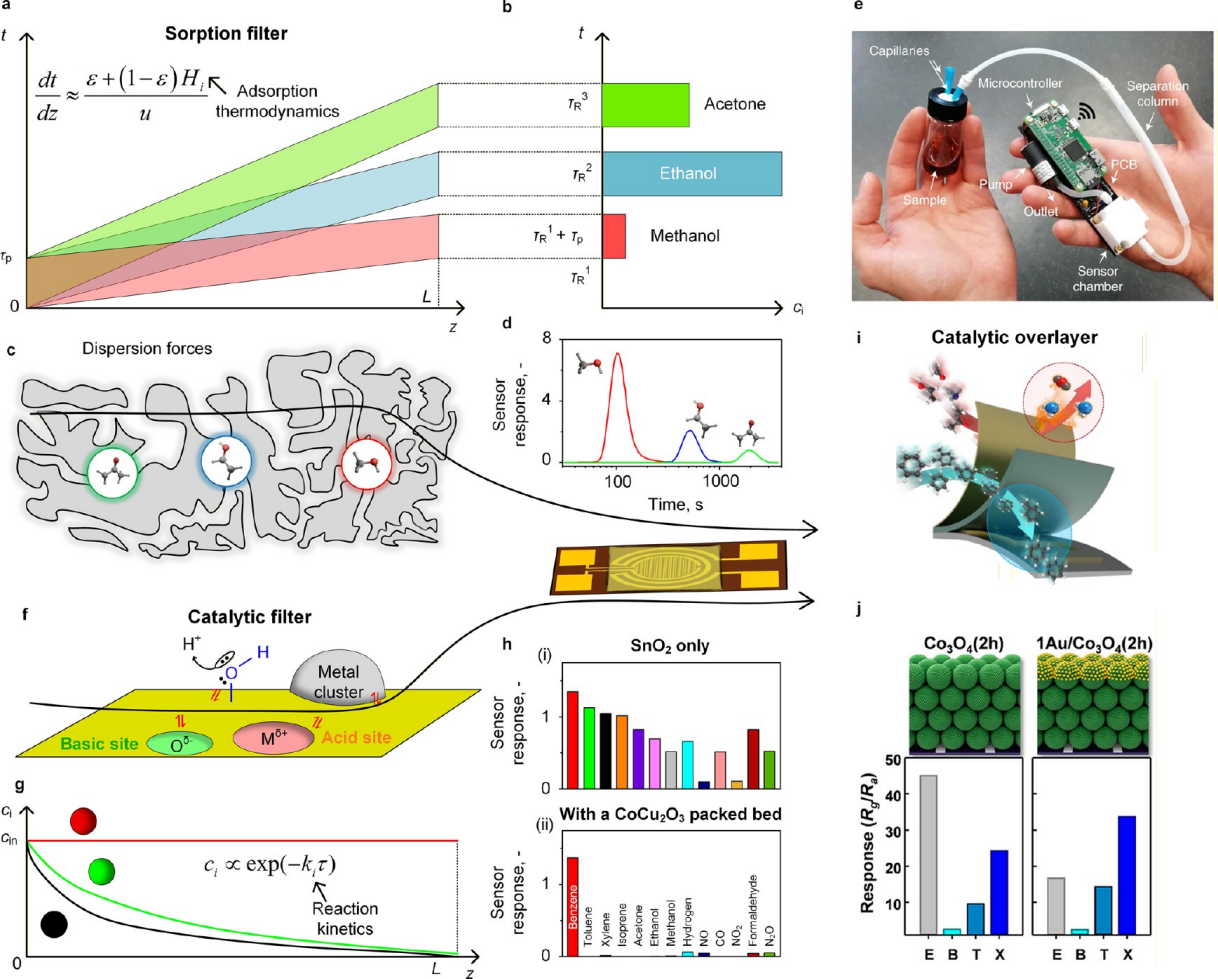
(a) Working principle of a sorption filter: a packed bed behaves like a miniaturized GC column and temporally separates analytes according to their different retention time (τ_R_). (b) Schematic elution order for three VOCs with different τ_R_. (c) Example separation of methanol (red), ethanol (blue), and acetone (green) on a hydrophobic sorption bed, where methanol elutes first. (d) The separated peaks are detected downstream by a single MO*_x_* sensor, which now reads a sequence of partially resolved transients rather than a single, mixed response. Reproduced from ref 484. Available under a CC-BY 4.0 license. Copyright 2019 The authors. (e) Device-level implementation of a Tenax TA sorption column integrated upstream of a VOC sensor for the selective quantification of methanol in the headspace of adulterated alcoholic beverages. Adapted with permission from ref 485. Copyright 2020 Nature. (f) Principle of catalytic filters, which precondition the gas matrix based on (g) differences in chemical reactivity over the catalyst surface. (h) Example: when a CoCu_2_O_3_ catalyst is operated at 170 °C at an appropriate space velocity, a broadly cross-sensitive SnO_2_ sensor becomes highly selective to benzene, even against chemically similar aromatic compounds at much higher concentrations. Reproduced from ref 363. Available under a CC-BY 4.0 license. Copyright 2024 The authors. (i) Concept of a catalytic overlayer directly on top of a chemoresistive sensor. Reproduced from ref 486. Available under a CC-BY 4.0 license. Copyright 2023 The authors. (j) Example implementation, where an Au-overlayered Co_3_O_4_ strongly enhances *p*-xylene (X) selectivity over ethanol (E), toluene (T), and benzene (B). Reproduced from ref 487. Copyright 2019 American Chemical Society.

In the example of Figure 13c, when feeding a methanol:ethanol:acetone mixture for a certain duration (GC pulse, τ_p_), their distinct retention times (τ_R_) allow the fairly nonselective MO*_x_* sensor downstream to record a time-resolved fingerprint instead of a superimposed signal (Figure 13d).^484^ That way, the sensor system discriminates methanol within a chosen time window from interfering analytes, the simple working principle of a research-prototype hand-held adulterated liquor analyzer shown in Figure 13e.^485^ There, a short Tenax TA column, a mini-pump, and a thin Pd/SnO_2_ film deposited on a suspended-membrane MHP together deliver exceptional methanol selectivity despite the presence of orders of magnitude higher concentrated ethanol background. This sensor system is capable of quantifying methanol in alcoholic distillates,^488^ hand-sanitizers,^489^ and exhaled breath for intoxication diagnostics.^353^ Such concepts can be rapidly translated into commercial products^490^ following interlaboratory validation according to ISO 5725.^491^ By exploiting the degrees of freedom in the design space of such packed-bed sorption columns, performance can be flexibly altered to achieve sensor systems for other analytes including formaldehyde.^492^

Catalytic filters offer a very promising route to meet the stringent selectivity requirements of real-world gas sensing and to overcome some intrinsic limitations of sorption-based preseparation. In fact, in miniaturized sensor systems with sorption columns, strongly retained analytes elute only after long delay times; their peaks are broadened and flattened by axial dispersion (reducing sensitivity), and the measurement itself is inherently discontinuous because the sensor can only be read out during short elution windows. In contrast, catalytic filters discriminate species through oxidation kinetics over heated catalyst beds rather than τ_R_. As the gas matrix passes through the catalyst (Figure 13f), interferants and target molecules are, in principle, converted at (i) *different rates* and (ii) *different products*. For trace analytes in air, where O_2_ is present in excess, the surface reaction rate is well approximated as pseudo-first-order;^493^ integrating the reaction rate along the residence time coordinate yields an exponentially decaying concentration profile. In some cases, target analytes may be only mildly reactive and pass through the catalyst almost unaffected (e.g., red line in Figure 13g), while unwanted interferants are converted to “*sensor-inactive*” species, such as CO_2_ (so-called oxidative filtering).^67^ In other nuanced circumstances, the target analyte is reformed on the catalyst surface to more “*sensor-active*” species, while the interferants are still converted to *inactive* CO_2_ (so-called reforming enhancement).^494^

As illustrated in Figure 13h, a CoCu_2_O_3_ catalytic fixed-bed reactor operated at 170 °C strongly suppresses a broad range of VOCs yet leaves benzene intact, rendering a typical SnO_2_ sensor placed downstream as a selective benzene detector. In this configuration, benzene was quantified down to 15 ppb even in the presence of up to 5000 ppb of other VOCs, including chemically similar aromatics such as toluene and xylene.^363^ Despite this excellent performance, packed-bed catalytic filters require their own resistive heaters, unless operated at room temperature,^309^ which compromises power consumption and increases the complexity of system integration.

As schematically illustrated in Figure 13i, catalytic filters can also be implemented as a catalytic overlayer on top of the sensing film to yield more compact integration and to avoid additional heaters.^486^ Gas molecules must diffuse through this reactive layer before reaching the sensing material, so the same oxidation kinetics apply but with an effective residence time set by diffusion and film pore size rather than by convective flow through a macroscopic packed bed. In many reported systems, however, the dominant mechanism remains unclear, largely because detailed catalytic characterization (e.g., conversion–selectivity measurements under realistic conditions) in such μm-thick overlayers is challenging. We see a clear opportunity for more systematic reaction studies to guide the design of overlayer architectures that deliberately shape individual sensor response patterns—for instance, tuning xylene selectivity as demonstrated in Figure 13j. Such overlayer-modified sensors also provide a more informative starting point for sensor array development, where multiple different overlayer structures have already been combined for air quality tasks such as selective detection and discrimination of aromatic VOCs.^487^

## 5 Conclusions

Air pollution driven by the release of hazardous volatiles from anthropogenic sources has become a global concern, threatening human health and our ecosystem. Air quality detectors are needed for emission control, but the research and development of suitable sensors has been a challenge due to their interdisciplinary nature at the cross-section of chemistry, physics, materials science, and engineering. Nevertheless, the scientific communities have made great advances in recent years across disciplines ranging from the development of new sensing concepts, materials, systems, and devices to achieve air pollutant detection under real-world conditions.

Our review has identified various chemoresistive material classes, ranging from metal oxides (MO*_x_*) to conducting polymers (Table 2), that are capable of approaching or even fulfilling present air pollutant exposure guidelines. Notably, a considerable number of these materials can detect gases such as NO_2_, NH_3_, CH_4_, H_2_S, acetone, and formaldehyde, some even at low operational temperatures and under realistic humidity conditions. Yet, the chemoresistive sensing of other critical pollutants, for instance, volatile halide compounds or organochlorines such as chloroform (CHCl_3_) and carbon tetrachloride (CCl_4_) as well as aromatic ethylbenzene and styrene, remains mostly unexplored and offers opportunities for further research. We therefore position this review as a framework to (i) identify under-investigated pollutant targets and (ii) evaluate emerging sensor concepts against best-in-class chemoresistive benchmarks in guideline-relevant concentration regimes.

## 6 Challenges and Future Outlook

From a sensor mechanistic perspective, a deeper investigation to obtain useful structure–activity correlations that can inform a predictive sensor design will be needed. Despite a large number of studies performing electronic structure and energy calculations to obtain, for instance, adsorption energies and/or net charge transfer, experimental validation is frequently missing, for instance, by X-ray-based spectroscopic techniques. Even fewer cases, albeit with some notable exceptions,^385–387^ perform such material and surface characterization under *in situ* and *operando* conditions. This could be explored in future work combining standard catalytic characterizations such as temperature-programmed chemisorption and comprehensive reaction analysis, elucidating both the catalyst kinetics (turnover frequency, apparent activation energy, reaction orders) and product distribution, which have been largely overlooked across the literature.

Other challenges include the reproducibility of sensing performance outside a well-controlled laboratory space, which requires thorough formulation and, most importantly, communication of experimental protocols. Finally, future efforts will enable us to integrate these sensor materials into suitable platforms for alarm systems and mobile robotic devices (such as drones and robot dogs) for distributed and automated air quality monitoring. In summary, the field of air quality sensor research offers significant opportunities for the scientific community to drive innovations with an immediate impact on industry and our society.

## References

[1] World Health Organization . World Health Statistics 2025: Monitoring Health for the SDGs, Sustainable Development Goals; Geneva, 2025 https://www.who.int/publications/i/item/9789240110496 (accessed 2025–11–18).

[2] Tran H. M., Tsai F. J., Lee Y. L., Chang J. H., Chang L. Te., Chang T. Y., Chung K. F., Kuo H. P., Lee K. Y., Chuang K. J., Chuang H. C. (2023). The Impact of Air Pollution on Respiratory
Diseases in an Era of Climate Change: A Review of the Current Evidence. Sci. Total Environ..

[3] Fuller R., Landrigan P. J., Balakrishnan K., Bathan G., Bose-O’Reilly S., Brauer M., Caravanos J., Chiles T., Cohen A., Corra L., Cropper M., Ferraro G., Hanna J., Hanrahan D., Hu H., Hunter D., Janata G., Kupka R., Lanphear B., Lichtveld M., Martin K., Mustapha A., Sanchez-Triana E., Sandilya K., Schaefli L., Shaw J., Seddon J., Suk W., Téllez-Rojo M. M., Yan C. (2022). Pollution and Health:
A Progress Update. Lancet Planet. Health.

[4] World Health Organization . Ambient (outdoor) air pollution, World Health Organization, https://www.who.int/news-room/fact-sheets/detail/ambient-%28outdoor%29-air-quality-and-health. (accessed 2025–11–18).

[5] Cascio W. E. (2018). Wildland
Fire Smoke and Human Health. Sci. Total Environ..

[6] Schollaert C., Connolly R., Cushing L., Jerrett M., Liu T., Marlier M. (2025). Air Quality Impacts
of the January 2025 Los Angeles
Wildfires: Insights from Public Data Sources. Environ. Sci. Technol. Lett..

[7] Reid C. E., Brauer M., Johnston F. H., Jerrett M., Balmes J. R., Elliott C. T. (2016). Critical Review
of Health Impacts of Wildfire Smoke
Exposure. Environ. Health Perspect..

[8] Lin F., Li G., Wang Y., Dong P., Yang K., Liu H., Xie N., Liu J., Chen H., Liu X., Li H., Li X., Li D., Sun S., Wang X., Sun Y., Li J., Zhao G., Chen Z., Pu J. (2025). Impacts of Air Pollutions
on Cardiovascular and Cerebrovascular Diseases through Inflammation:
A Comprehensive Analysis of One Million Chinese and Half Million UK
Individuals. J. Transl. Med..

[9] Liu J. C., Wilson A., Mickley L. J., Ebisu K., Sulprizio M. P., Wang Y., Peng R. D., Yue X., Dominici F., Bell M. L. (2017). Who Among the Elderly Is Most Vulnerable
to Exposure
to and Health Risks of Fine Particulate Matter From Wildfire Smoke?. Am. J. Epidemiol..

[10] Hajat A., Hsia C., O’Neill M. S. (2015). Socioeconomic
Disparities and Air
Pollution Exposure: A Global Review. Curr. Environ.
Health Rep..

[11] Kim J. T., Lee C. W., Jung H. J., Choi H. J., Salman A., Padmajan Sasikala S., Kim S. O. (2022). Application of 2D
Materials for Adsorptive
Removal of Air Pollutants. ACS Nano.

[12] Wang M., Kim R. Y., Kohonen-Corish M. R.
J., Chen H., Donovan C., Oliver B. G. (2025). Particulate Matter Air Pollution
as a Cause of Lung Cancer: Epidemiological and Experimental Evidence. Br. J. Cancer.

[13] Saxena P., Sonwani S. (2019). Criteria Air Pollutants
and Their Impact on Environmental
Health. Crit. Air Pollut. Impact Environ. Health.

[14] Halios C. H., Landeg-Cox C., Lowther S. D., Middleton A., Marczylo T., Dimitroulopoulou S. (2022). Chemicals
in European Residences
– Part I: A Review of Emissions, Concentrations and Health
Effects of Volatile Organic Compounds (VOCs). Sci. Total Environ..

[15] Hernik A., Smets J., Wang Z., Semenova Y., Tan J., Ameloot R., Naydenova I. (2025). Optical Detection
of Volatile Organic
Compounds: A Review of Methods and Functionalized Sensing Materials. Adv. Opt. Mater..

[16] Janfaza S., Khorsand B., Nikkhah M., Zahiri J. (2019). Digging Deeper into
Volatile Organic Compounds Associated with Cancer. Biol. Methods Protoc..

[17] Xiong Y., Zhou J., Xing Z., Du K. (2021). Cancer Risk Assessment
for Exposure to Hazardous Volatile Organic Compounds in Calgary, Canada. Chemosphere.

[18] Xia C., Capozzi S. L., Romanak K. A., Lehman D. C., Dove A., Richardson V., Greenberg T., McGoldrick D., Venier M. (2024). The Ins and Outs of
Per- and Polyfluoroalkyl Substances
in the Great Lakes: The Role of Atmospheric Deposition. Environ. Sci. Technol..

[19] Zhou X., Yue X., Tian C., Lu X. (2024). Global Assessment of Climatic Responses
to Ozone-Vegetation Interactions. Atmos. Chem.
Phys..

[20] Lie Z., Huang W., Zhou G., Zhang D., Yan J., Jiang J., Neilson R., Zhou S., Zhang W., Ramos Aguila L. C., Chu G., Liu S., Meng Z., Zhang Q., Liu J. (2023). Acidity of Soil and Water Decreases
in Acid-Sensitive Forests of Tropical China. Environ. Sci. Technol..

[21] Lewis A., Edwards P. (2016). Validate Personal Air-Pollution
Sensors. Nature.

[22] Najafi P., Ghaemi A. (2024). Chemiresistor Gas Sensors:
Design, Challenges, and
Strategies: A Comprehensive Review. Chem. Eng.
J..

[23] Fennell J. F., Liu S. F., Azzarelli J. M., Weis J. G., Rochat S., Mirica K. A., Ravnsbæk J. B., Swager T. M. (2016). Nanowire Chemical/Biological
Sensors: Status and a Roadmap for the Future. Angew. Chem., Int. Ed..

[24] Bulemo P. M., Kim D. H., Shin H., Cho H. J., Koo W. T., Choi S. J., Park C., Ahn J., Güntner A. T., Penner R. M., Kim I. D. (2025). Selectivity
in Chemiresistive Gas
Sensors: Strategies and Challenges. Chem. Rev..

[25] Banga I., Paul A., Poudyal D. C., Muthukumar S., Prasad S. (2023). Recent Advances in Gas Detection Methodologies with
a Special Focus on Environmental Sensing and Health Monitoring Applications—A
Critical Review. ACS Sens..

[26] Brattain W. H., Bardeen J. (1953). Surface Properties
of Germanium. Bell Syst. Tech. J..

[27] Heiland G. (1954). On the influence
of adsorbed oxygen on the electrical conductivity of zinc oxide crystals. J. Phys..

[28] Seiyama T., Kato A., Fujiishi K., Nagatani M. (1962). A New Detector for
Gaseous Components Using Semiconductive Thin Films. Anal. Chem..

[29] Ghaderi A., Sabbaghzadeh J., Dejam L., Behzadi Pour G., Moghimi E., Matos R. S., da Fonseca Filho H. D., Ṭălu Ştefan., Salehi shayegan A., Aval L. F., Astani Doudaran M., Sari A., Solaymani S. (2024). Nanoscale
Morphology, Optical Dynamics and Gas Sensor of Porous Silicon. Sci. Rep..

[30] Yoon J. W., Choi S. H., Kim J. S., Jang H. W., Kang Y. C., Lee J. H. (2016). Trimodally Porous SnO2 Nanospheres with Three-Dimensional
Interconnectivity and Size Tunability: A One-Pot Synthetic Route and
Potential Application as an Extremely Sensitive Ethanol Detector. NPG Asia Mater..

[31] Chen K., Zhu L., Wang J., Xie W., Deng Y., Xue L., Long H., Wan H., Ren J., Yuan K., Wang W., Yao Q., Zhao D., Chen X., Deng Y. (2025). Active Polymer-Templated Porous Metal Oxide Nanospheres with Tailored
Single-Atom Modification for Olfactory Intelligence. J. Am. Chem. Soc..

[32] Güntner A. T., Pineau N. J., Chie D., Krumeich F., Pratsinis S. E. (2016). Selective
Sensing of Isoprene by Ti-Doped ZnO for Breath Diagnostics. J. Mater. Chem. B.

[33] Sun Y., Zhao Z., Suematsu K., Zhang W., Zhang W., Zhuiykov S., Shimanoe K., Hu J. (2022). MOF-Derived Au-NiO/In2O3
for Selective and Fast Detection of Toluene at Ppb-Level in High Humid
Environments. Sens. Actuators, B.

[34] Suematsu K., Harano W., Oyama T., Shin Y., Watanabe K., Shimanoe K. (2018). Pulse-Driven Semiconductor
Gas Sensors Toward Ppt Level
Toluene Detection. Anal. Chem..

[35] Yoo R., Güntner A. T., Park Y., Rim H. J., Lee H. S., Lee W. (2019). Sensing of
Acetone by Al-Doped ZnO. Sens. Actuators,
B.

[36] Lee K., Kim M., Kwon Y., Park S., Lim Y., Kwak D., Jeong J., Kim B., Ahn J., Kim J., Cho Y.-H., Kim I.-D., Park I. (2025). Enabling Weather-Independent
Gas Detection through Deep Learning on Light-Activated Sensors. ACS Nano.

[37] Kassem O., Saadaoui M., Rieu M., Viricelle J. P. (2019). A Novel
Approach to a Fully Inkjet Printed SnO2-Based Gas Sensor on a Flexible
Foil. J. Mater. Chem. C.

[38] Nematbakhsh
Abkenar G., Rieu M., Breuil P., Viricelle J. P. (2021). Development
of a Selective Ammonia YSZ-Based Sensor and Modeling of Its Response. Sens. Actuators, B.

[39] Sanghikian
Marques dos Santos G., dos Santos Theodoro R., Gera G. O., Micheli
Perfecto T., Paschoalini Volanti D. (2025). Ethyl Acetate Detection Using Mixed-Phase
In2O3 Nanorods. ACS Appl. Nano Mater..

[40] Srinivasan P., Samanta S., Krishnakumar A., Rayappan J. B. B., Kailasam K. (2021). Insights into
G-C3N4 as a Chemi-Resistive Gas Sensor for VOCs and Humidity –
a Review of the State of the Art and Recent Advancements. J. Mater. Chem. A.

[41] Banagiri S., Doctor Z., Kalogera V. al., Lai J.-Y., Saka N., Chun J.-H., Reuter C., Vieira J. D., Spilker J. S., Wright J. S., Lim W., Norton D. P., Pearton S. J., Ren F., Johnson J. L., Ural A. (2010). Nitride and Oxide Semiconductor Nanostructured
Hydrogen Gas Sensors. Semicond. Sci. Technol..

[42] Chatterjee B., Bandyopadhyay A. (2023). Review on
the Synthesis of Metal Sulfides Gas Sensors
and Their Performances at Room Temperature. Mater. Sci. Eng., B.

[43] Tang H., Sacco L. N., Vollebregt S., Ye H., Fan X., Zhang G. (2020). Recent Advances in 2D/Nanostructured
Metal Sulfide-Based Gas Sensors:
Mechanisms, Applications, and Perspectives. J. Mater. Chem. A.

[44] Pervaiz S., Saeed M. U., Ali H., Saeed Y., Khan A., Alanazi Y. M. (2025). Monolayer CuBr-Based
Gas Sensor to Detect Habitat and
Industry-Relevant Molecules with High Sensitivity and Selectivity:
A First-Principles Study. RSC Adv..

[45] Güntner A. T., Wied M., Pineau N. J., Pratsinis S. E. (2020). Rapid and
Selective NH_3_ Sensing by Porous CuBr. Adv. Sci..

[46] de
Sá B. S., Perfecto T. M., de Oliveira D. S., Volanti D. P. (2023). Microwave-Assisted Solvothermal Synthesis of In-MIL-68
Derived Hollow In2O3 Microrods for Enhanced 1-Pentanol Sensing Performance. Mater. Today Commun..

[47] Theodoro R. S., Sá B. S., Perrone O. M., Perfecto T. M., Volanti D. P. (2023). Tuning
Humidity for Highly Selective Detection of Methanol and 2-Butanone
Using MOF-Derivatives NiO Microrods. J. Mater.
Sci.: Mater. Electron..

[48] Wang S., McGuirk C. M., d’Aquino A., Mason J. A., Mirkin C. A. (2018). Metal–Organic
Framework Nanoparticles. Adv. Mater..

[49] Benedetto G., Stolz R. M., Meng Z., Chan J. Y. M., Shehayeb E. O., Morrell C. T., Fabusola G., Elsaesser N., Simon C. M., Mirica K. A. (2025). Conductive Covalent Organic Frameworks
as Chemiresistive Sensor Arrays for the Detection and Differentiation
of Gasotransmitters. J. Am. Chem. Soc..

[50] Zhong, Z. ; Damacet, P. ; Sánchez-González, E. ; Eagleton, A. M. ; Vereshchuk, N. ; Wongratanaphisan, R. ; Anderson, J. T. ; Goncalves, S. ; Peterson, G. W. ; Blount, B. ; Monti, S. ; Barcaro, G. ; Ibarra, I. A. ; Mirica, K. A. Scalable Templated Fabrication of a Cu-Based MOF on Textiles for Simultaneous Sensing, Filtration, and Detoxification of SO2. Chem 2025, 11 (10). 102580 10.1016/j.chempr.2025.102580. 41393254 PMC12700617

[51] Park C., Woo J., Jeon M., Baek J. W., Shin E., Kim J., Park S., Kim I.-D. (2025). Dual-MOF-Layered Films via Solution
Shearing Approach: A Versatile Platform for Tunable Chemiresistive
Sensors. ACS Nano.

[52] Güntner A. T., Abegg S., Wegner K., Pratsinis S. E. (2018). Zeolite
Membranes for Highly Selective Formaldehyde Sensors. Sens. Actuators, B.

[53] Sahner K., Hagen G., Schönauer D., Reiß S., Moos R. (2008). Zeolites — Versatile Materials
for Gas Sensors. Solid State Ionics.

[54] Luo S. X. L., Swager T. M. (2024). Wireless Detection
of Trace Ammonia: A Chronic Kidney
Disease Biomarker. ACS Nano.

[55] Perfecto T. M., Zito C. A., Mazon T., Volanti D. P. (2018). Flexible Room-Temperature
Volatile Organic Compound Sensors Based on Reduced Graphene Oxide–WO3·0.33H2O
Nano-Needles. J. Mater. Chem. C.

[56] Zito C. A., Perfecto T. M., Volanti D. P. (2017). Impact
of Reduced Graphene Oxide
on the Ethanol Sensing Performance of Hollow SnO2 Nanoparticles under
Humid Atmosphere. Sens. Actuators, B.

[57] Perfecto T. M., Zito C. A., Volanti D. P. (2016). Room-Temperature
Volatile Organic
Compounds Sensing Based on WO3·0.33H2O, Hexagonal-WO3, and Their
Reduced Graphene Oxide Composites. RSC Adv..

[58] Degler D., Weimar U., Barsan N. (2019). Current Understanding
of the Fundamental
Mechanisms of Doped and Loaded Semiconducting Metal-Oxide-Based Gas
Sensing Materials. ACS Sens..

[59] Humayun M., Bououdina M., Usman M., Khan A., Luo W., Wang C. (2024). Designing
State-of-the-Art Gas Sensors: From Fundamentals to Applications. Chem. Rec..

[60] Goel N., Kunal K., Kushwaha A., Kumar M. (2023). Metal Oxide Semiconductors
for Gas Sensing. Eng. Rep..

[61] da
Silva Gropelo H., dos Santos Theodoro R., Sanghikian Marques dos
Santos G., Micheli Perfecto T., Paschoalini Volanti D. (2025). Crystallite
Size Control in MOF-5-Derived Nanostructured ZnO Sensors for Detecting
1-Pentanol. J. Alloys Compd..

[62] Lim S., Nguyen K. V., Lee W. H. (2024). Enhancing
Sensitivity in Gas Detection:
Porous Structures in Organic Field-Effect Transistor-Based Sensors. Sensors.

[63] Ahmad N., Kanjariya P., Priya G. P., Kumar A., Thakur R., Sharma R., Kumari M., Kaur S., Mishra M. K. (2025). Recent
Advances on the Gas-Sensing Properties and Mechanism of Perovskite
Oxide Materials - A Review. ACS Omega.

[64] Sharma A., Eadi S. B., Noothalapati H., Otyepka M., Lee H. D., Jayaramulu K. (2024). Porous Materials as Effective Chemiresistive Gas Sensors. Chem. Soc. Rev..

[65] Li T., Zeng W., Wang Z. (2015). Quasi-One-Dimensional
Metal-Oxide-Based
Heterostructural Gas-Sensing Materials: A Review. Sens. Actuators, B.

[66] Ji H., Zeng W., Li Y. (2019). Gas Sensing Mechanisms of Metal Oxide
Semiconductors: A Focus Review. Nanoscale.

[67] Weber I. C., Güntner A. T. (2022). Catalytic
Filters for Metal Oxide Gas Sensors. Sens. Actuators,
B.

[68] Fu X., Qiao Z., Zhou H., Xie D. (2024). Defect Engineering
in Transition Metal Dichalcogenide-Based Gas Sensors. Chemosensors.

[69] Ciftyurek E., Li Z., Schierbaum K. (2023). Adsorbed Oxygen
Ions and Oxygen Vacancies: Their Concentration
and Distribution in Metal Oxide Chemical Sensors and Influencing Role
in Sensitivity and Sensing Mechanisms. Sensors.

[70] Ding W., Liu D., Liu J., Zhang J. (2020). Oxygen Defects in Nanostructured
Metal-Oxide Gas Sensors: Recent Advances and Challenges†. Chin. J. Chem..

[71] Zhang C., Liu G., Geng X., Wu K., Debliquy M. (2020). Metal Oxide Semiconductors
with Highly Concentrated Oxygen Vacancies for Gas Sensing Materials:
A Review. Sens. Actuators, A.

[72] Jo Y. M., Jo Y. K., Lee J. H., Jang H. W., Hwang I. S., Yoo D. J. (2023). MOF-Based Chemiresistive
Gas Sensors: Toward New Functionalities. Adv.
Mater..

[73] Dong H., Koenig G. M. (2020). A Review on Synthesis and Engineering of Crystal Precursors
Produced via Coprecipitation for Multicomponent Lithium-Ion Battery
Cathode Materials. CrystEngComm.

[74] Guex L. G., Sacchi B., Peuvot K. F., Andersson R. L., Pourrahimi A. M., Ström V., Farris S., Olsson R. T. (2017). Experimental
Review: Chemical Reduction of Graphene Oxide (GO) to Reduced Graphene
Oxide (RGO) by Aqueous Chemistry. Nanoscale.

[75] Sui R., Charpentier P. (2012). Synthesis
of Metal Oxide Nanostructures by Direct Sol–Gel
Chemistry in Supercritical Fluids. Chem. Rev..

[76] Güntner A. T., Pineau N. J., Pratsinis S. E. (2022). Flame-Made Chemoresistive Gas Sensors
and Devices. Prog. Energy Combust. Sci..

[77] Lang K., Liu T., Padilla D. J., Nelson M., Landorf C. W., Patel R. J., Ballentine M. L., Kennedy A. J., Shih W. S., Scotch A., Zhu J. (2024). Nanofibers
Enabled Advanced Gas Sensors: A Review. Adv.
Sens. Energy Mater..

[78] Lee J., Kim M., Park S., Ahn J., Kim I. (2025). Materials
Engineering
for Light-Activated Gas Sensors: Insights, Advances, and Future Perspectives. Adv. Mater..

[79] Dennler N., True A., van Schaik A., Schmuker M. (2025). Neuromorphic Principles
for Machine Olfaction. Neuromorph. Comput. Eng..

[80] Güntner A. T., Abegg S., Königstein K., Gerber P. A., Schmidt-Trucksäss A., Pratsinis S. E. (2019). Breath
Sensors for Health Monitoring. ACS Sens..

[81] Bilgin M. B., Shin H., Jutzeler C. R., Kessler T. M., Slack E., Egli A., Güntner A. T. (2025). Microbial and Antimicrobial Resistance
Diagnostics by Gas Sensors and Machine Learning. Cell Biomater..

[82] Güntner A. T., Gerber P. A., Dittrich P. S., Serra N., Figalli A., Puhan M. A., Beuschlein F. (2025). Challenges
and Opportunities of Wearable
Molecular Sensors in Endocrinology and Metabolism. Nat. Rev. Endocrinol..

[83] Nasiri N., Clarke C. (2019). Nanostructured Chemiresistive
Gas Sensors for Medical
Applications. Sensors.

[84] Nasiri N., Clarke C. (2019). Nanostructured Gas
Sensors for Medical and Health Applications:
Low to High Dimensional Materials. Biosensors.

[85] Qian L., Rahmati F., Li F., Zhang T., Wang T., Zhang H., Yan S., Zheng Y. (2025). Recent Advances in
2D MXene-Based Heterostructures for Gas Sensing: Mechanisms and Applications
in Environmental and Biomedical Fields. Nanoscale.

[86] Wang J., Wang R. (2025). Development of Gas
Sensors and Their Applications in Health Safety,
Medical Detection, and Diagnosis. Chemosensors.

[87] Verma G., Gupta A. (2025). Next-Generation Chemiresistive
Wearable Breath Sensors for Non-Invasive
Healthcare Monitoring: Advances in Composite and Hybrid Materials. Small.

[88] Mahapatra C. (2025). Recent Advances
in Medical Gas Sensing with Artificial Intelligence-Enabled Technology. Med. Gas Res..

[89] Elias
Abi-Ramia Silva T., Burisch F., Güntner A. T. (2024). Gas Sensing
for Space: Health and Environmental Monitoring. TrAC, Trends Anal. Chem..

[90] Isaac N. A., Pikaar I., Biskos G. (2022). Metal Oxide Semiconducting
Nanomaterials
for Air Quality Gas Sensors: Operating Principles, Performance, and
Synthesis Techniques. Microchim. Acta.

[91] Barot, V. ; Kapadia, V. Air Quality Monitoring Systems Using IoT: A Review. In 2020 International Conference on Computational Performance Evaluation, ComPE 2020; IEEE, 2020; pp 226–231 10.1109/COMPE49325.2020.9200053.

[92] Saini J., Dutta M., Marques G. (2021). Sensors for Indoor Air Quality Monitoring
and Assessment through Internet of Things: A Systematic Review. Environ. Monit. Assess..

[93] Saini J., Dutta M., Marques G. (2020). Indoor Air Quality
Monitoring Systems
Based on Internet of Things: A Systematic Review. International J. Environ. Res. Public Health.

[94] Garcia A., Saez Y., Harris I., Huang X., Collado E. (2025). Advancements
in Air Quality Monitoring: A Systematic Review of IoT-Based Air Quality
Monitoring and AI Technologies. Artif. Intell.
Rev..

[95] Zhang H., Srinivasan R. (2020). A Systematic
Review of Air Quality Sensors, Guidelines,
and Measurement Studies for Indoor Air Quality Management. Sustainability.

[96] Saini J., Dutta M., Marques G. (2020). A Comprehensive Review on Indoor
Air Quality Monitoring Systems for Enhanced Public Health. Sustainable Environ. Res..

[97] Amin
Beigh F., Rasool Shah S., Ahmad K. (2025). A Perspective on Indoor
Air Quality Monitoring, Guidelines, and the Use of Various Sensors. Environ. Forensics.

[98] Noruzzade E., Mirzaei N., Janghorbanian S., Hassanvand M., Nikkhoo N., Khanizadeh M. (2025). A Systematic
Global Review of Fixed
Air Quality Monitoring Stations: Spatial Distribution, Typologies,
Measured Pollutants, Technologies, Regulatory Standards, and Research
Gaps. J. Air Pollut. Health.

[99] Panda S., Mehlawat S., Dhariwal N., Sanger A., Kumar A. (2024). Comprehensive
Review on Gas Sensors: Unveiling Recent Developments and Addressing
Challenges. Mater. Sci. Eng., B.

[100] Sheoran K., Siwal S. S., Kapoor D., Singh N., Saini A. K., Alsanie W. F., Thakur V. K. (2022). Air Pollutants
Removal
Using Biofiltration Technique: A Challenge at the Frontiers of Sustainable
Environment. ACS Eng. Au.

[101] Montero-Montoya R., López-Vargas R., Arellano-Aguilar O. (2018). Volatile Organic
Compounds in Air: Sources, Distribution, Exposure and Associated Illnesses
in Children. Ann. Global Health.

[102] Saxena P., Shukla P. (2023). A Review on Recent
Developments and
Advances in Environmental Gas Sensors to Monitor Toxic Gas Pollutants. Environ. Prog. Sustainable Energy.

[103] Feng T., Shi Y., Wang X., Wan X., Mi Z. (2025). Synergies of Air Pollution Control Policies: A Review. J. Environ. Manage..

[104] Račić N., Terzić I., Karlović N., Bošnjaković A., Terzić T., Jakovljević I., Pehnec G., Horvat T., Gajski G., Gerić M., Vitko S., Šunić I., Forsmann M., Avino P., Banić I., Lipej M., Malev O., Žegura B., Switters J., Mureddu F., Lovrić M. (2025). Volatile Organic
Compounds (VOCs) and Polycyclic Aromatic Hydrocarbons (PAHs) in Indoor
Environments: A Review and Analysis of Measured Concentrations in
Europe. Indoor Air.

[105] Banga I., Paul A., Poudyal D. C., Muthukumar S., Prasad S. (2023). Recent Advances in Gas Detection
Methodologies with
a Special Focus on Environmental Sensing and Health Monitoring Applications—A
Critical Review. ACS Sens..

[106] Zhou X., Zhou X., Wang C., Zhou H. (2023). Environmental
and Human Health Impacts of Volatile Organic Compounds: A Perspective
Review. Chemosphere.

[107] Zhou L., Jiao X., Yang B., Yuan W., Zhao W., Zhang L., Huang W., Long S., Xu J., Shen H., Wang C. (2025). The Impact
of Indoor Environments
on the Abundance of Urban Outdoor VOCs. Environ.
Sci. Technol..

[108] Mangotra A., Singh S. K. (2024). Volatile Organic Compounds: A Threat
to the Environment and Health Hazards to Living Organisms –
A Review. J. Biotechnol..

[109] Verma G., Gokarna A., Kadiri H., Nomenyo K., Lerondel G., Gupta A. (2023). Multiplexed Gas Sensor:
Fabrication
Strategies, Recent Progress, and Challenges. ACS Sens..

[110] U.S. Environmental Protection Agency Health Effects Notebook for Hazardous Air Pollutants: Methanol: Washington, DC, 2000; https://www.epa.gov/sites/default/files/2016-09/documents/methanol.pdf. (accessed 2025–11–21).

[111] European Chemicals Agency (ECHA) . Substance Infocard - Methanol, European Chemicals Agency, https://echa.europa.eu/substance-information/-/substanceinfo/100.000.599. (accessed 2025–11–21).

[112] National Health Commission (NHC), C. GBZ 2.1–2019: Occupational Exposure Limits for Hazardous Agents in the Workplace — Part 1: Chemical Hazardous Agents; Beijing, 2019 https://files.chemicalwatch.com/GBZ2.1-2019.pdf(accessed 2025–11–21).

[113] U.S. Occupational Safety and Health . OSHA Occupational Chemical Database: Ethyl Alcohol (CAS 64–17–5), IMIS 1034; Washington, DC, 2023 https://www.osha.gov/chemicaldata/1034(accessed 2025–11–19).

[114] National Institute for Occupational Safety and Health (NIOSH) . NIOSH Pocket Guide to Chemical Hazards: Ethyl Alcohol, Centers for Disease Control and Prevention (CDC), https://www.cdc.gov/niosh/npg/npgd0262.html#print. (accessed 2025–11–21).

[115] Labbox Labware, S. L. Safety Data Sheet: NU1170 Ethanol 96% AGR, ACS, Ph.Eur., CAS 64–17–5; Barcelona, 2022 https://labbox.es/wp-content/uploads/FDS/SDS_EN_10204.pdf(accessed 2025–11–21).

[116] American Conference of Governmental Industrial Hygienists (ACGIH) . Acetone, ACGIH, https://www.cdc.gov/niosh/idlh/67641.html. (accessed 2025–11–21).

[117] European Commission . COMMISSION DIRECTIVE 2000/39/EC of 8 June 2000; Brussels, 2000 https://eur-lex.europa.eu/legal-content/EN/TXT/?uri=CELEX%3A02000L0039-20210520(accessed 2025–11–21).

[118] American Conference of Governmental Industrial Hygienists (ACGIH) . Formaldehyde, ACGIH, https://www.acgih.org/formaldehyde-2/. (accessed 2025–11–21).

[119] U.S. Environmental Protection Agency . Health Effects Notebook for Hazardous Air Pollutants: Formaldehyde; Washington, DC, 2000 https://www.epa.gov/sites/default/files/2016-09/documents/formaldehyde.pdf(accessed 2025–11–20).

[120] Institute for Occupational Safety and Health of the German Social Accident Insurance (IFA) . Verbindliche Arbeitsplatzgrenzwerte der EU-Kommission, DGUV/IFA, https://www.dguv.de/ifa/fachinfos/occupational-exposure-limit-values/verbindliche-arbeitsplatzgrenzwerte-der-eu-kommission/index.jsp. (accessed 2025–11–21).

[121] European Chemicals Agency (ECHA) . Formaldehyde (CAS 50–00–0, ID 100.000.002), ECHA, https://echa.europa.eu/substance-information/-/substanceinfo/100.000.002. (accessed 2025–11–21)..

[122] National Development and Reform Commission of China (NDRC) . (Indoor Air Quality Standard); Beijing, 2022 https://www.ndcpa.gov.cn/jbkzzx/c100201/1666357812062392320/ZgwODgwF.pdf(accessed 2025–11–22).

[123] U.S. Environmental Protection Agency . Health Effects Notebook for Hazardous Air Pollutants: Acetaldehyde; Washington, DC, 2000 https://www.epa.gov/sites/default/files/2016-09/documents/acetaldehyde.pdf(accessed 2025–11–21).

[124] American Conference of Governmental and Industrial Hygienists (ACGIH) . Acetaldehyde, ACGIH, https://www.acgih.org/acetaldehyde/. (accessed 2025–11–21).

[125] International Labour Organization; International Programme on Chemical Safety/World Health Organization . ICSC 0009: Acetaldehyde (Ethanal), ILO-WHO ICSC Database, https://chemicalsafety.ilo.org/dyn/icsc/showcard.display?p_card_id=0009. (accessed 2025–11–21).

[126] European Chemicals Agency (ECHA) . Acrylaldehyde (CAS 107–02–8), ECHA, https://echa.europa.eu/substance-information/-/substanceinfo/100.003.141. (accessed 2025–11–21).

[127] U.S. Environmental Protection Agency; National Institute for Occupational Safety and Health . Health Effects Notebook for Hazardous Air Pollutants: Acrolein. 2009 https://www.epa.gov/sites/default/files/2016-08/documents/acrolein.pdf. (accessed 2025–11–21).

[128] U.S. Occupational Safety and Health Administration (OSHA) . Acrolein (CAS No. 107–02–8), OSHA, https://www.osha.gov/chemicaldata/51. (accessed 2025–11–21).

[129] American Conference of Governmental Industrial Hygienists (ACGIH) . Benzene, ACGIH, https://www.acgih.org/benzene-2/. (accessed 2025–11–22).

[130] European Chemicals Agency (ECHA) . Benzene (CAS 71–43–2), ECHA, https://echa.europa.eu/substance-information/-/substanceinfo/100.000.685. (accessed 2025–11–22).

[131] U.S. Environmental Protection Agency . Health Effects Notebook for Hazardous Air Pollutants: Benzene; Washington, DC, 2012 https://echa.europa.eu/substance-information/-/substanceinfo/100.003.297(accessed 2025–11–22).

[132] Committee on Hazardous Substances (AGS) . TRGS 910 “Risk-Related Concept of Measures for Activities Involving Carcinogenic Hazardous Substances”. 2023 https://www.baua.de/EN/Service/Technical-rules/TRGS/TRGS-910(accessed 2025–11–22).

[133] European Commission . EU air quality standards, European Commission, https://environment.ec.europa.eu/topics/air/air-quality/eu-air-quality-standards_en. (accessed 2025–11–22).

[134] American Conference of Governmental Industrial Hygienists (ACGIH) . Toluene, ACGIH, https://www.acgih.org/toluene/. (accessed 2025–11–22).

[135] European Chemicals Agency (ECHA) . Toluene (CAS 108–88–3), ECHA, https://echa.europa.eu/substance-information/-/substanceinfo/100.003.297. (accessed 2025–11–22).

[136] U.S. Environmental Protection Agency . Health Effects Notebook for Hazardous Air Pollutants: Toluene; Washington, DC, 2012 https://www.epa.gov/sites/default/files/2016-09/documents/toluene.pdf(accessed 2025–11–22).

[137] American Conference of Governmental Industrial Hygienists (ACGIH) . Xylene (all isomers) (CAS Nos. 95–47–6, 108–38–3, 106–42–3), ACGIH, https://www.acgih.org/xylene-all-isomers/. (accessed 2025–11–22).

[138] U.S. Environmental Protection Agency . Health Effects Notebook for Hazardous Air Pollutants: Xylenes (CAS Nos. 95–47–6, 108–38–3, 106–42–3); Washington, DC, 2000 https://www.epa.gov/haps/health-effects-notebook-hazardous-air-pollutants(accessed 2025–11–22).

[139] European Parliament & Council . Regulation (EC) No 1831/2003 — Annexes, Official Journal of the European Union/European Union, https://www.legislation.gov.uk/eudr/2019/1831/annex. (accessed 2025–11–22).

[140] American Conference of Governmental Industrial Hygienists (ACGIH) . Styrene (CAS 100–42–5), ACGIH, https://www.acgih.org/styrene. (accessed 2025–11–22).

[141] Plastics Europe . Styrene Monomer: Safe Handling Guide; Brussels, 2024 https://plasticseurope.org/wp-content/uploads/2018/09/Styrene_SAFE_HANDLING_GUIDE_2024_FINAL_24102024_v2.pdf?(accessed 2025–11–22).

[142] U.S. Environmental Protection Agency . Health Effects Notebook for Hazardous Air Pollutants: Styrene; Washington, DC, 2000 https://www.epa.gov/sites/default/files/2020-05/documents/styrene_update_2a.pdf(accessed 2025–11–22).

[143] U.S. Environmental Protection Agency . Health Effects Notebook for Hazardous Air Pollutants: Ethylbenzene; Washington, DC, 2000 https://www.epa.gov/sites/default/files/2016-09/documents/ethylbenzene.pdf(accessed 2025–11–22).

[144] Health and Safety Executive (HSE) . EH40/2005 Workplace Exposure Limits; London, 2020 https://www.hse.gov.uk/pubns/priced/eh40.pdf?(accessed 2025–11–22).

[145] American Conference of Governmental Industrial Hygienists (ACGIH) . Hydrogen Sulfide (CAS No. 7783–06–4), ACGIH, https://www.acgih.org/hydrogen-sulfide/. (accessed 2025–11–22).

[146] European Chemicals Agency (ECHA) . Hydrogen sulphide (ID 100.029.070), ECHA, https://echa.europa.eu/substance-information/-/substanceinfo/100.029.070. (accessed 2025–11–22).

[147] U.S. Environmental Protection Agency . Health Effects Notebook for Hazardous Air Pollutants: Chloroform; Washington, DC, 2000 https://www.epa.gov/sites/default/files/2016-09/documents/chloroform.pdf(accessed 2025–11–22).

[148] European Chemicals Agency (ECHA) . Chloroform (CAS 67–66–3), ECHA, https://echa.europa.eu/substance-information/-/substanceinfo/100.000.603. (accessed 2025–11–22).

[149] American Conference of Governmental Industrial Hygienists (ACGIH) . Carbon tetrachloride (CAS 56–23–5), ACGIH, https://www.acgih.org/carbon-tetrachloride/. (accessed 2025–11–22).

[150] European Chemicals Agency (ECHA) . Carbon tetrachloride (CAS 56–23–5), ECHA, https://echa.europa.eu/substance-information/-/substanceinfo/100.000.239. (accessed 2025–11–22).

[151] U.S. Environmental Protection Agency . Health Effects Notebook for Hazardous Air Pollutants: Carbon Tetrachloride; Washington, DC, 2000 https://www.epa.gov/sites/default/files/2016-09/documents/carbon-tetrachloride.pdf(accessed 2025–11–22).

[152] National Research Council. Committee on Toxicology . Emergency and Continuous Exposure Limits for Selected Airborne Contaminants, National Academies Press: Washington, D.C., 1984; Vol. 1, 10.17226/690.

[153] International Agency for Research on Cancer (IARC) . Outdoor Air Pollution, Mattock, H. ; Müller, K. , Eds.; IARC/World Health Organization: Lyon, 2015; Vol. 109.

[154] World Health Organization . WHO Global Air Quality Guidelines: Particulate Matter (PM2.5 and PM10), Ozone, Nitrogen Dioxide, Sulfur Dioxide and Carbon Monoxide, World Health Organization: Geneva, 2021. 34662007

[155] American Conference of Governmental Industrial Hygienists (ACGIH) . Carbon Monoxide (CAS No. 630–08–0), ACGIH, https://www.acgih.org/carbon-monoxide-3/. (accessed 2025–11–22).

[156] European Chemicals Agency (ECHA) . Carbon monoxide (CAS 630–08–0), ECHA, https://echa.europa.eu/substance-information/-/substanceinfo/100.010.118. (accessed 2025–11–22).

[157] Ministry of Ecology and Environment of the People’s Republic of China (MEE) . Ambient Air Quality Standard; Beijing, 2016 https://english.mee.gov.cn/Resources/standards/Air_Environment/quality_standard1/201605/W020160511506615956495.pdf(accessed 2025–11–22).

[158] Occupational Safety and Health Administration (OSHA) . Carbon Dioxide (CAS 124–38–9), OSHA, https://www.osha.gov/chemicaldata/183. (accessed 2025–11–22).

[159] European Chemicals Agency (ECHA) . Carbon dioxide (CAS 124–38–9), ECHA, https://echa.europa.eu/substance-information/-/substanceinfo/100.004.271. (accessed 2025–11–22).

[160] American Conference of Governmental Industrial Hygienists (ACGIH) . Nitrogen Dioxide (CAS No. 10102–44–0), ACGIH, https://www.acgih.org/nitrogen-dioxide. (accessed 2025–11–22).

[161] European Chemicals Agency (ECHA) . Nitrogen dioxide (CAS 10102–44–0), ECHA, https://echa.europa.eu/substance-information/-/substanceinfo/100.030.234. (accessed 2025–11–22).

[162] Occupational Safety and Health Administration (OSHA) . Ammonia (CAS 7664–41–7), U.S. Department of Labor/OSHA, https://www.osha.gov/chemicaldata/623. (accessed 2025–11–22).

[163] European Commission . Directive 2000/39/EC of 8 June 2000 Establishing a First List of Indicative Occupational Exposure Limit Values in Implementation of Council Directive 98/24/EC on the Protection of the Health and Safety of Workers from the Risks Related to Chemical Agents at Work; Brussels, 2000 https://eur-lex.europa.eu/legal-content/EN/TXT/?uri=CELEX%3A02000L0039-20210520(accessed 2025–11–22).

[164] American Conference of Governmental Industrial Hygienists (ACGIH) . Ozone (CAS 10028–15–6), ACGIH, https://www.acgih.org/ozone/. (accessed 2025–11–22).

[165] American Conference of Governmental Industrial Hygienists (ACGIH) . Sulfur dioxide (CAS 7446–09–5), ACGIH, https://www.acgih.org/sulfur-dioxide. (accessed 2025–11–22).

[166] Liu J., Yu L., He X., Zhang Y., Shi C., Li S., Li C., Zhou C. (2023). Hollow Urchin-Like Ag-Doped In_2_O_3_ Nanomaterials for Enhanced Low-Temperature Methanol
Sensing Under UV Irradiations. ACS Appl. Nano
Mater..

[167] Barceloux D. G., Bond G. R., Krenzelok E. P., Cooper H., Vale J. A. (2002). American Academy of Clinical Toxicology
Practice Guidelines on the Treatment of Methanol Poisoning. J. Toxicol. Clin. Toxicol..

[168] Rostrup M., Edwards J. K., Abukalish M., Ezzabi M., Some D., Ritter H., Menge T., Abdelrahman A., Rootwelt R., Janssens B., Lind K., Paasma R., Hovda K. E. (2016). The Methanol Poisoning Outbreaks
in Libya 2013 and Kenya 2014. PLoS One.

[169] Schultz, K. ; Kumar, H. Over 90 Killed in India by Toxic Homemade Liquor, The New York Times. February 23, 2019; https://www.nytimes.com/2019/02/23/world/asia/india-poison-alcohol.html. (accessed 2025–11–20).

[170] Almeida, D. Brazil Reports 209 Suspected Cases of Methanol Poisoning. Agência Brasil. Brasília October 6, 2025 https://agenciabrasil.ebc.com.br/en/saude/noticia/2025-10/brazil-reports-209-suspected-cases-methanol-poisoning(accessed 2025–11–20).

[171] National Institute for Occupational Safety and Health (NIOSH) . Acetone - IDLH, Centers for Disease Control and Prevention (CDC)/NIOSH, https://www.cdc.gov/niosh/idlh/67641.html#print. (accessed 2025–11–20).

[172] Salthammer T., Mentese S., Marutzky R. (2010). Formaldehyde in the
Indoor Environment. Chem. Rev..

[173] Gupta K.
C., Ulsamer A. G., Preuss P. W. (1982). Formaldehyde in
Indoor Air: Sources and Toxicity. Environ. Int..

[174] Zervas E. (2008). Regulated and Non-Regulated Pollutants Emitted from
Two Aliphatic and a Commercial Diesel Fuel. Fuel.

[175] PAZ O., JANES H. W., PREVOST B. A., FRENKEL C. (1982). Enhancement
of Fruit
Sensory Quality by Post-Harvest Applications of Acetaldehyde and Ethanol. J. Food Sci..

[176] Sittig, M. Handbook of Toxic and Hazardous Chemicals and Carcinogens, 2nd ed.; Noyes Publications, 1985.

[177] Salthammer T. (2023). Acetaldehyde in the Indoor Environment. Environ. Sci.: Atmos..

[178] U.S. Environmental Protection Agency . Health Effects Notebook for Hazardous Air Pollutants: Acetaldehyde; Washington, DC, 2000 https://www.epa.gov/sites/default/files/2016-09/documents/acetaldehyde.pdf (accessed 2025–11–20).

[179] Zhang D., Pan W., Zhou L., Yu S. (2021). Room-Temperature
Benzene Sensing with Au-Doped ZnO Nanorods/Exfoliated WSe_2_ Nanosheets and Density Functional Theory Simulations. ACS Appl. Mater. Interfaces.

[180] Wallace L. A. (1989). Major Sources of Benzene Exposure. Environ. Health Perspect..

[181] World Health Organization; International Programme on Chemical Safety . Environmental Health Criteria 150: Benzene; Geneva, 1993 https://www.inchem.org/documents/ehc/ehc/ehc150.htm(accessed 2025–11–20).

[182] International Agency for Research on Cancer (IARC) . List of Classifications by Cancer Site; Lyon, 2025 https://monographs.iarc.who.int/wp-content/uploads/2019/07/Classifications_by_cancer_site.pdf(accessed 2025–11–20).

[183] Oliveira K., Guevara M., Jorba O., Petetin H., Bowdalo D., Tena C., Montané Pinto G., López F., Pérez García-Pando C. (2024). On the Uncertainty
of Anthropogenic Aromatic Volatile Organic Compound Emissions: Model
Evaluation and Sensitivity Analysis. Atmos.
Chem. Phys..

[184] Liu J. S., Wang N., Zhang X. F., Deng Z. P., Xu Y. M., Huo L. H., Gao S. (2021). Facile Tree Leaf-Templated
Synthesis of Mesoporous CeO_2_ Nanosheets for Enhanced Sensing
Detection of p-Xylene Vapors. J. Alloys Compd..

[185] Agency for Toxic Substances and Disease Registry (ATSDR) . Toxicological Profile for Toluene; Atlanta, GA, 2017 https://www.atsdr.cdc.gov/toxprofiles/tp56.pdf(accessed 2025–11–20). 37289924

[186] Agency for Toxic Substances and Disease Registry (ATSDR) . Toxicological Profile for Xylenes; Atlanta, GA, 2007 https://www.atsdr.cdc.gov/toxprofiles/tp71.pdf(accessed 2025–11–20).

[187] Ali F. I. M., Awwad F., Greish Y. E., Mahmoud S. T. (2019). Hydrogen
Sulfide (H 2 S) Gas Sensor: A Review. IEEE Sens.
J..

[188] Sen A., Albarella J. D., Carey J. R., Kim P., McNamara W. B. (2008). Low-Cost
Colorimetric Sensor for the Quantitative Detection of Gaseous Hydrogen
Sulfide. Sens. Actuators, B.

[189] Kim K. H., Choi Y., Jeon E., Sunwoo Y. (2005). Characterization
of Malodorous Sulfur Compounds in Landfill Gas. Atmos. Environ..

[190] Lewis R. J., Copley G. B. (2015). Chronic Low-Level
Hydrogen Sulfide
Exposure and Potential Effects on Human Health: A Review of the Epidemiological
Evidence. Crit. Rev. Toxicol..

[191] Han J., Li H., Cheng J., Ma X., Fu Y. (2025). Advances in
Metal Oxide Semiconductor Gas Sensor Arrays Based on Machine Learning
Algorithms. J. Mater. Chem. C.

[192] Patra C., Guna V. K., Chakraborty S., Mondal S., Nandakishora Y. (2025). Advances in
2D Transition Metal Dichalcogenide-Based
Gas Sensors. ACS Sens..

[193] Hou J., Lee C. H., Park S., Kim H., Hilal M., Cai Z. (2025). Recent Advances in Engineering 2D
Transition Metal Dichalcogenides
for High-Performance Gas Sensing. Sens. Actuators,
A.

[194] Shafiee S. A., Perry S. C., Hamzah H. H., Mahat M. M., Al-lolage F. A., Ramli M. Z. (2020). Recent Advances
on Metal Nitride
Materials as Emerging Electrochemical Sensors: A Mini Review. Electrochem. Commun..

[195] Zhou K., Tang L., Zhu C., Tang J., Su H., Luo L., Chen L., Zeng D. (2024). Recent Advances in
Structure Design and Application of Metal Halide Perovskite-Based
Gas Sensor. ACS Sens..

[196] Rawat A., Chourasia N. K., Rajput G., Sneha N., Kulriya P. K. (2025). Review of MXene-Based
Nanocomposites for Gas Sensing. ACS Appl. Nano
Mater..

[197] Nguyen L. H. T., Mirzaei A., Kim J. Y., Phan T. B., Tran L. D., Wu K. C. W., Kim H. W., Kim S. S., Doan T. L. H. (2025). Advancements in MOF-Based Resistive Gas Sensors: Synthesis
Methods and Applications for Toxic Gas Detection. Nanoscale Horiz..

[198] Majhi S. M., Ali A., Rai P., Greish Y. E., Alzamly A., Surya S. G., Qamhieh N., Mahmoud S. T. (2022). Metal–Organic
Frameworks for Advanced Transducer Based Gas Sensors: Review and Perspectives. Nanoscale Adv..

[199] Peng X., Wu X., Zhang M., Yuan H. (2023). Metal–Organic
Framework Coated Devices for Gas Sensing. ACS
Sens..

[200] Alzate-Carvajal N., Luican-Mayer A. (2020). Functionalized
Graphene Surfaces
for Selective Gas Sensing. ACS Omega.

[201] Yeow J. T. W., Wang Y. (2009). A Review of Carbon Nanotubes-Based
Gas Sensors. J. Sens..

[202] Park S. J., Park C. S., Yoon H. (2017). Chemo-Electrical
Gas
Sensors Based on Conducting Polymer Hybrids. Polymers.

[203] Yan Y., Yang G., Xu J. L., Zhang M., Kuo C. C., Wang S. D. (2020). Conducting Polymer-Inorganic Nanocomposite-Based Gas
Sensors: A Review. Sci. Technol. Adv. Mater..

[204] Yang X. F., Wang A., Qiao B., Li J., Liu J., Zhang T. (2013). Single-Atom Catalysts: A New Frontier in Heterogeneous
Catalysis. Acc. Chem. Res..

[205] Walther A., Müller A. H. E. (2013). Janus
Particles: Synthesis, Self-Assembly,
Physical Properties, and Applications. Chem.
Rev..

[206] Zhu L., Wang H., Qiu C., Li M., Li Q., An F., Song Y., Wang T., Jiao G., Ding Z., Yang Z., Zhang D. (2024). Carbon Monoxide Gas Sensor Based
on Hydrothermally Synthesized Pd–CuO Nanorods/SnSe_2_ Nanoflower Heterojunctions. ACS Appl. Nano
Mater..

[207] Zhang Y., Xiong H., Chen Q., Tang M., Wang Z., Bian J., Zhang D. (2025). Hollow-Sphere NiO/Ti_3_C_2_T_x_-Based Gas Sensor for CO Sensing. ACS Appl. Nano Mater..

[208] Lian M., Luan K., He T., Chen D. (2025). Rational Design
of NiCo_2_O_4_/Ti_3_C_2_ MXene
Heterostructure for Unmanned Aerial Vehicles-Enabled Real-Time CO
Monitoring. ACS Sens..

[209] Rudra P., Rahaman M., Velaga S., Mondal S. (2025). Mesoporous
Boron Subphosphide: Intrinsic Electron Deficiency Enabling Selective
Low-Ppm of Chemiresistive CO Detection in Harsh Environments. ACS Appl. Mater. Interfaces.

[210] Liu X., Wu J., Fan C., Zhang Y., Quan W., Li J., Hu N., Yang J., Zeng M., Yang Z. (2025). One-Dimensional
Conductive Metal–Organic Frameworks Enable Real-Time Chemiresistive
Detection of Thermal Runaway Gases. ACS Sens..

[211] Qin Y., Zhang Y., Lei J., Liu X. (2025). Achieving Switchable
CO/H_2_ Detection with Selectivity-Tunable SnO_2_–Co_3_O_4_ Sensors. ACS Appl. Mater. Interfaces.

[212] Qin C., Wei Z., Zhao X., Sun J., Cao J., Wang Y. (2025). Enhancing the Surface Activity of Co_3_O_4_ by
Gallium Doping to Increase the Sensitivity of Conductometric CO Gas
Sensors. Sens. Actuators, B.

[213] Wang W., Cao J., Wang S., Zhang R., Zhang Y. (2024). CuO–SnO_2_ Sensor
for Room-Temperature CO Detection:
Experiments and DFT Calculations. Sens. Actuators,
B.

[214] Pineau N. J., Keller S. D., Güntner A. T., Pratsinis S. E. (2020). Palladium Embedded in SnO_2_ Enhances the
Sensitivity of Flame-Made Chemoresistive Gas Sensors. Microchim. Acta.

[215] Haldar T., Shiu J. W., Yang R. X., Wang W. Q., Wu H. T., Mao H. I., Chen C. W., Yu C. H. (2024). Exploring
MOF-Derived CuO/RGO Heterostructures for Highly Efficient Room Temperature
CO_2_ Sensors. ACS Sens..

[216] Wang X., Xu X., Zhou T., Zhang T. (2025). Room-Temperature
CO_2_ Monitoring Platform Enabled by Alkali Metal Functionalization
of a Mg-MOF-74-Based QCM Sensor. ACS Sens..

[217] Haldar T., Chen C. W., Shiu J. W., Wu H. T., Rwei S. P., Yu C. H. (2025). Urchin-like TiO_2_ Microspheres
Coupled with MXene Nanosheets for CO_2_ Detection. ACS Appl. Nano Mater..

[218] Xu X., Zhou T., Yang A., Jiang H., Song Z., Wang X., Bing Y., Zhao L., Zhang T. (2025). Mixed-Matrix
Membrane-Based Piezoelectric CO_2_ Sensor with Self-Humidity
Compensation. ACS Sens..

[219] Ding Z., Chen Q., Zhang H., Wang W., Zhang D. (2025). Carbon Dioxide Gas Sensor Based on
the LaCoO_3_/MXene Heterojunction. ACS Appl. Nano Mater..

[220] Aleksanyan M., Sayunts A., Shahkhatuni G., Simonyan Z., Kananov D., Papovyan R., Kopecký D. (2025). Fabrication
and Characterization of MWCNTs Decorated ZnO Nanograins Based Sensor
for Enhanced Performance Toward CO_2_ Gas. Adv. Mater. Interfaces.

[221] Xu H., Li J., Li P., Shi J., Gao X., Luo W. (2021). Highly Efficient SO_2_ Sensing
by Light-Assisted Ag/PANI/SnO_2_ at Room Temperature and
the Sensing Mechanism. ACS Appl. Mater. Interfaces.

[222] Leangtanom P., Wisitsoraat A., Tuantranont A., Chanlek N., Pookmanee P., Satienperakul S., Phanichphant S., Kruefu V. (2023). Microwave-Assisted
Hydrothermal/Impregnation
Synthesis of Cu_2_O-Decorated RGO/In_2_O_3_ Nanorices for Sensitive SO_2_ Gas Sensors. ACS Appl. Nano Mater..

[223] He X., Ren X., An X., Guo P., Hu Y., Liu T., Zhang J. (2025). Detection of Ppb-Level
SO_2_ at Room Temperature
Based on Bi_2_O_2_Se Nanosheets. ACS Sens..

[224] Sharma M., Patel C., Samal A., Sriram S., Mukherjee S., Das A. K. (2024). Tailoring Thiazole
Decorated Polymer
with Benzoselenadiazole for Enhanced SO_2_ Sensing. ACS Appl. Polym. Mater..

[225] Gond R., Barala S., Shukla P., Bassi G., Kumar S., Kumar M., Kumar M., Rawat B. (2025). Fe_2_O_3_-Functionalized MoS2 Nanostructure Sensor
for High-Sensitivity
and Low-Level SO_2_ Detection. ACS
Sens..

[226] Wang S., Fu Y., Wang F., Wang X., Yang Y., Wang M., Wang J., Lin E., Ma H., Chen Y., Cheng P., Zhang Z. (2024). Scalable Melt Polymerization
Synthesis of Covalent Organic Framework Films for Room Temperature
Low-Concentration SO_2_ Detection. J. Am. Chem. Soc..

[227] Zhou W., Gu G., Gao Y., Han J., Yu N., Liao C., Lu G. (2025). Humidity-Tolerant Ppb-Level
NOx Sensors
Based on Ag_2_Te/CeO_2_ Nanocomposites for Smart
Greenhouse Farming. ACS Sens..

[228] Xiao R., Pang L., Lai X., Fan W., Lu Z., Gao J. (2025). Antimony Doping in SnO_2_ Nanoparticles for
Sensitive NO_2_ Sensors. ACS Sens..

[229] Chu S. Y., Wu M. J., Yeh T. H., Lee C. T., Lee H. Y. (2024). Sensing
Mechanism and Characterization of NO_2_ Gas Sensors Using
Gold-Black NP-Decorated Ga_2_O_3_ Nanorod Sensing
Membranes. ACS Sens..

[230] Zheng Q., Wang T., Zhang G., Zhang X., Huang C., Cheng X., Huo L., Cui X., Xu Y. (2024). Synergy of Active Sites and Charge Transfer in Branched
WO_3_/W_18_O_49_ Heterostructures for Enhanced
NO_2_ Sensing. ACS Sens..

[231] Han J., Gu G., Gao Y., Yu N., Zhou W., Wang Y., Kong D., Gao Y., Lu G. (2024). Prototype
Alarm Integrating Pulse-Driven Nitrogen Dioxide Sensor Based on Holey
Graphene Oxide/In_2_O_3_. ACS Sens..

[232] Hwa Y., Kim B., Park H., Je Y., Ryu M. D., Lee J. W., Jo Y. R., An H. R., Son B., Jeong B. Y., Kong J. S., Kim T. H., Ryou M., Kim Y. J., Ryu G. H., Jung H., Kang J., Chee S. S. (2025). Phase Engineering
of SnSe_X_ (X = 1,2) Microstructures
for High-Performance NO_2_ Chemiresistive Room-Temperature
Sensor Systems: Toward Highly Reliable and Robust Detection Properties
under Humidity and Interfering Gas Conditions. ACS Sens..

[233] Kumar S., Betal A., Kumar A., Chakkar A. G., Kumar P., Kwoka M., Sahu S., Kumar M. (2025). Enhancing
NO_2_ Gas Sensing: The Dual Impact of UV and Thermal Activation
on Vertically Aligned Nb-MoS_2_ for Superior Response and
Selectivity. ACS Sens..

[234] Kushwaha A., Bharti N. R., Sharma A., Kedia S. K., Gupta G., Goel N. (2024). Enhanced NO_2_ Gas Sensing
in Nanocrystalline MoS_2_ via Swift Heavy Ion Irradiation:
An Experimental and DFT Study. ACS Sens..

[235] Li X., Wang Y., Liu X., Li J., Wu J., Zeng M., Yang J., Hu N., Zhu H., Xu L., Yang Z. (2025). Trace Detection of
Nitrogen Dioxide via Porous Tin
Dioxide Nanopods with High Specific Surface Area and Enhanced Charge
Transfer. ACS Sens..

[236] Komorizono A. A., Leite R. R., De la
Flor S., Llobet E., Mastelaro V. R. (2025). Flexible Gas Sensor Based on RGO-ZnO
for NO_2_ Detection at Room Temperature. Mater. Sci. Semicond. Process..

[237] Wang S., Hu H., Tan T., Li X., Zhou W., Tian Z., Bao Y., Homewood K. P., Muhammad S., Xia X., Gao Y. (2025). Enhancing NO_2_ Sensing Performance through Interface Engineering in Cs_2_AgBiBr_6_/SnO_2_/ZnO-NRs Sensor. Sens. Actuators, B.

[238] Sun S., Li X., Sun Y., Wang N., Huang B., Li X. (2025). Fabrication of TeNT/TeO_2_ Heterojunction Based Sensor for
Ultrasensitive Detection of NO_2_. J. Hazard. Mater..

[239] Kim J., Li M., Lin C. H., Hu L., Wan T., Saeed A., Guan P., Feng Z., Kumeria T., Tang J., Su D., Wu T., Chu D. (2025). Synergetic
Phase Modulation and N-Doping of MoS_2_ for Highly Sensitive
Flexible NO_2_ Sensors. Adv. Sci..

[240] Cheng X., Liu Y., Zhong W., Li S., Li Y., Zhao Z., Zhang C., Shi J., Liu H., Zhu Z., Xu F. (2025). An Ultra-Selective and Humidity-Resistant Room-Temperature-Operated
NO_2_ Sensor Based on Black TiO_2_. Adv. Sci..

[241] Baut A., Pereira Martins M., Güntner A. T. (2025). Template-Free
Synthesis of Highly Porous Metal Nitride Architectures for Electronics
and Molecular Sensing. Small Struct..

[242] Hersberger S., Martins M. P., Fassbind S., Güntner A. T. (2025). Selective,
Stable and Humidity-Robust NO_2_ Sensing at Room Temperature
with Porous WS_2_ Films. ACS Sens..

[243] Abegg S., Cerrejon D. K., Güntner A. T., Pratsinis S. E. (2020). Thickness
Optimization of Highly Porous Flame-Aerosol
Deposited WO_3_ Films for NO_2_ Sensing at Ppb. Nanomaterials.

[244] Jung S., Roman C., Hierold C. (2024). Fast Nitrogen
Dioxide
Sensing with Ultralow-Power Nanotube Gas Sensors. Adv. Sens. Res..

[245] Hu T., Dong Q., Gong X., Zhang Y., Jiang Z., Wang X., Chu Z. (2025). Efficient Synthesis of Transition
Metal Dichalcogenides/Reduced Graphene Oxide van Der Waals Heterojunctions
and Their Exceptional Sensitivity for NO_2_ Detection. Sens. Actuators, B.

[246] Cao P. J., Cai Y. Z., Pawar D., Han S., Xu W. Y., Fang M., Liu X. K., Zeng Y. X., Liu W. J., Lu Y. M., Zhu D. L. (2022). Au@ZnO/RGO Nanocomposite-Based
Ultra-Low Detection Limit Highly Sensitive and Selective NO_2_ Gas Sensor. J. Mater. Chem. C.

[247] Kumar S., Dmitrieva V. A., Meng G., Evlashin S. A., Sukhanova E. V., Kvashnin D. G., Popov Z. I., Bannov A. G., Fedorov F. S., Nasibulin A. G. (2023). Structured Graphene Oxide/Reduced
Graphene Oxide Interfaces for Improved NO_2_ Sensing. ACS Appl. Nano Mater..

[248] Wu Y., Li W., Chang Y., Gao Y., Wang F., Li H., French P. J., Lee Y. K., Akbar S. A., M Umar
Siddiqui A., Wang Y., Zhou G. (2025). NO Detection on Exposed
Fe–N4 Sites Deposited on Nanometer-Sized Cu-Hemin MOFs Coated
on Reduced Graphene Oxide at Room Temperature. ACS Appl. Nano Mater..

[249] Wang R., Ma T., Jin Q., Xu C., Yang X., Wang K., Wang X. (2024). Waveguide-Based Microwave
Nitric Oxide Sensor for COVID-19 Screening: Mass Transfer Modulation
Effect on Hollow Confined WO_3_ Structures. ACS Sens..

[250] Zhao J., Li P., Zhang Q., Ye Z., Wang Y., Tian J., Xu Z., Peng N., Ren H., Zhang X. (2024). MOF Nanoflowers-Based Flexible Portable NO Sensors
for Human Airway Inflammation Detection. Chem.
Eng. J..

[251] Si R., Jiang H., Xie S., Guo X., Zhang S. (2025). Extremely
Sensitive, Highly Selective and Fast Recovery NO Sensor Based on WO_3_ Modified Nanomaterials for Application to Exhaled Breath
Analyzers. Sens. Actuators, B.

[252] Dhall S., Prakash J., Nigam A., Astakala A. K., Sood K. (2025). WO_3_-Based Chemiresistive
Sensors for NO Detection at Low
Temperatures. Microchem. J..

[253] Turlybekuly A., Sarsembina M., Mentbayeva A., Bakenov Z., Soltabayev B. (2023). CuO/TiO_2_ Heterostructure-Based
Sensors for Conductometric NO_2_ and N_2_O Gas Detection
at Room Temperature. Sens. Actuators, B.

[254] Ma J., He Z., Dai L., Liu Y., Zhu J., Liu H., Li Y., Meng W., Wang L. (2025). Mixed Potential Sensor
Using YSZ and BaMoO_4_-SE for Detection of Low Concentrations
N2O. Sens. Actuators, B.

[255] Ramu A. G., Umar A., Gopi S., Algadi H., Albargi H., Ibrahim A. A., Alsaiari M. A., Wang Y., Choi D. (2021). Tetracyanonickelate (II)/KOH/Reduced
Graphene Oxide Fabricated Carbon
Felt for Mediated Electron Transfer Type Electrochemical Sensor for
Efficient Detection of N_2_O Gas at Room Temperature. Environ. Res..

[256] Huang J., Wu H., Su Z. (2025). Ultra-High Performance
Fibrous Ammonia Sensor with Full Degradability. ACS Sens..

[257] Lin T., Li J. (2025). High-Performance Ammonia Sensing
Electrode Based on
a Graphene/Porphyrin–Polyaniline Nanocomposite Material. ACS Appl. Electron. Mater..

[258] Thangavel R., Sivaperuman K., Thirumalaisamy L., Pandiaraj S., Alruwaili M., Alanazi N., Alodhayb A. N., Abeykoon C., Grace A. N. (2025). Unleashing
Synergy: RGO Decorated
on a Cobalt-Doped ZnFe_2_O_4_ Film to Detect Trace-Level
NH_3_ at Ambient Temperature with High Response and Cross-Selectivity. ACS Appl. Electron. Mater..

[259] Liang J., Wu T., Chen H., Han Y., Fatima A., Afzal U., Zhang Y., Gao X., Yan W. (2025). Ag Nanoparticles for Room-Temperature NH_3_ Sensing Using
a Two-Dimensional Layered Ti_3_C_2_T_x_ MXene/V_2_O_5_ Nanostructured Composite. ACS Appl. Nano Mater..

[260] Wu H., Li D., Liu J., Gong X., Wang T., Zhao L., Wang T., Yan X., Liu F., Sun P., Lu G. (2024). Portable and Hand-Held
Ammonia Gas Sensor Enables Noninvasive
Prediagnosis of Helicobacter Pylori Infection. ACS Sens..

[261] Luo S., Liu B., Hu J., Zhang Y. (2025). Foldable and
Wearable
Fabric Ammonia Sensor Based on a Polyaniline/Polyacrylonitrile Nanofiber
for Smart Home Applications. Anal. Chem..

[262] Chi Cao L. T., Zhou M.-H., Opaprakasit P., Sreearunothai P., Nagao Y., Boonruang S., Fallah H., Tseng S.-F., Hsu S.-H., T Cao L. C., Opaprakasit P., Sreearunothai P., Fallah H., Hsu S., Zhou M., Tseng S., Nagao Y., Boonruang S. (2023). Facile Fabrication
of Oxygen-Enriched MXene-Based Sensor and Their Ammonia Gas-Sensing
Enhancement. Adv. Mater. Interfaces.

[263] Wang L. J., Wang M., Zheng K., Ye W., Zhang S., Wang B., Long X. (2024). High-Performance Room
Temperature Ammonia Sensors Based on Pure Organic Molecules Featuring
B-N Covalent Bond. Adv. Sci..

[264] Bai J., Shen Y., Zhao S., Li A., Kang Z., Cui B., Wei D., Yuan Z., Meng F. (2023). Room-Temperature NH_3_ Sensor Based on SnO_2_ Quantum
Dots Functionalized
SnS_2_ Nanosheets. Adv. Mater. Technol..

[265] Li X., Zeng W., Zhuo S., Qian B., Chen Q., Luo Q., Qian R. (2024). Highly Sensitive
Room-Temperature Detection of Ammonia
in the Breath of Kidney Disease Patients Using Fe_2_Mo_3_O_8_/MoO_2_@MoS_2_ Nanocomposite
Gas Sensor. Adv. Sci..

[266] Argyrou A., Giappa R. M., Gagaoudakis E., Binas V., Remediakis I., Brintakis K., Kostopoulou A., Stratakis E. (2025). Cs_2_AgBiBr_6_ Perovskites:
Designing Stable, Sensitive and Selective Eco-friendly Ozone Sensors. Adv. Sens. Res..

[267] Wang S., Yang Y., Li X., Wang T., Li J., Shi G., Wang G. (2024). Room Temperature
Solid Electrolyte
Ozone Sensor Based on Ag-Doped SnO_2_. Sens. Actuators, A.

[268] Ding J., Xie M., Li Z., Wang Y. (2025). Fabrication
of WO_3_ Nanosheets with Hexagonal/Orthorhombic Homojunctions
for Highly Sensitive Ozone Gas Sensors at Low Temperature. J. Alloys Compd..

[269] Tseng Z. C., Jiang Y. Y., Lin C. Y., Do J. Y., Hsu T. H., Shih C. W., Chang Y. Z., Liao S. Y., Huang C. Y. (2024). Highly Stable Flexible Ozone Gas Sensors Using Mn_3_O_4_ Nanoparticles-Decorated IGZO Thin Films through
the SILAR Method. Ceram. Int..

[270] Gagaoudakis E., Kampitakis V., Moschogiannaki M., Sfakianou A., Anthopoulos T., Tsetseris L., Kiriakidis G., Deligeorgis G., Iacovella F., Binas V. (2022). Low-Energy Consumption
CuSCN-Based Ultra-Low-Ppb Level Ozone Sensor,
Operating at Room Temperature. Sens. Actuators,
A.

[271] Zhao Z., Deng Z., Zhang R., Klamchuen A., He Y., Horprathum M., Chang J., Mi L., Li M., Wang S., Fang X., Meng G. (2023). Sensitive and Selective
Ozone Sensor Based on CuCo_2_O_4_ Synthesized by
a Facile Solution Combustion Method. Sens. Actuators,
B.

[272] Komorizono A. A., de Lima B. S., Mastelaro V. R. (2023). Assessment
of the Ozonolysis Effect of RGO-ZnO-Based Ozone Sensors. Sens. Actuators, B.

[273] Huang Q., Deng Z., Zhang R., Klamchuen A., Horprathum M., Wang S., Fang X., You L., Huang S., Meng G. (2024). Highly Sensitive and Selective Ppb-Level
Ozone Sensor Based on Porous CuO Nanoparticles. Sens. Actuators, B.

[274] Wang X., Li Y., Jin X., Sun G., Cao J., Wang Y. (2024). Effectively Improved CH4 Sensing Performance of In_2_O_3_ Porous Hollow Nanospheres by Doping with Cd. Langmuir.

[275] Chen R., Wang Z., Xia Y., Xiang L. (2025). Controlled
Doping Sites to Enhance Charge Transfer of ZnO for Ultrarapid Methane
Sensing. ACS Appl. Mater. Interfaces.

[276] Sahoo S. J., Maji B., Das A., Sharma R. K., Dash P. (2025). Metal–Organic
Framework-Templated Growth of Cation-Substituted
Metal Oxide Shell MO (M = Co, Ni, and Zn) on a CuO Core: An In-Depth
Understanding of Methane Gas-Sensing Performance. ACS Appl. Electron. Mater..

[277] Li A., Zhao S., Bai J., Wu M., Gao S., Shen Y., Yuan Z., Meng F. (2025). SnO_2_ Nanospheres
Coated with Pt or Pd as Electrode Materials for Detecting Hydrogen
and Methane. ACS Appl. Nano Mater..

[278] Li Z., Qi T., Zhao X., Zhang Y., Zhang Z., Wang T., Xiao X., Yang D. (2026). CeO_2_ Hollow
Nanospheres Decorated with Pd and PdO Nanodots for Fast Methane Sensing. Adv. Funct. Mater..

[279] Yuan C., Ma J., Cheng X., Cheng J., Yu H., Xu H., Deng Y. (2025). Rational Design of PdPt Nanoalloys
Sensitized Mesoporous SnO_2_ for High-Performance Methane
Sensing Applications. Adv. Mater. Technol..

[280] Liu Z., Shao X., Zhai J., Ding Z., Jiao G., Zhang D. (2025). A High Sensitivity and Fast Response Methane Sensor Based on SnO_2_/Zn_2_SnO_4_ Nanocomposite for Lithium-Ion
Battery Fault Detection. J. Alloys Compd..

[281] Wang D., Zhang D., Li Y., Zhang H., Lin L., Zhang X., Wang H., Yu Q. (2025). V_2_CT_x_ MXene/Ni-Metal Organic Framework-Derived
V_2_O_5_/NiO Nanohybrid Methane Sensors for Fault
Detection of Lithium-Ion
Battery. Sens. Actuators, B.

[282] Zhang Y., Chen F., Wang D., Wang T., Zhang D. (2023). Vanadium Dioxide/Molybdenum Telluride
Heterojunction Gas Sensor for
Methane Detection. J. Alloys Compd..

[283] Kgomo M. B., Shingange K., Nemufulwi M. I., Swart H. C., Mhlongo G. H. (2023). Belt-like In_2_O_3_ Based Sensor for Methane Detection: Influence
of Morphological,
Surface Defects and Textural Behavior. Mater.
Res. Bull..

[284] Sun X., Zhu L., Qin C., Cao J., Wang Y. (2023). Room-Temperature
Methane Sensors Based on ZnO with Different Exposed Facets: A Combined
Experimental and First-Principle Study. Surf.
Interfaces.

[285] Li X., He H., Tan T., Zou Z., Tian Z., Zhou W., Bao Y., Xia X., Gao Y. (2023). Annealing
Effect on the Methane Sensing Performance of Pt–SnO_2_/ZnO Double Layer Sensor. Appl. Surf. Sci..

[286] Sun X., Zhang Y., Wang Y., Li M., Qin C., Cao J., Wang Y. (2024). UV-Activated AuAg/ZnO
Microspheres for High-Performance
Methane Sensor at Room Temperature. Ceram. Int..

[287] Siraj S., Rybicki F. J., Sonkusale S., Sahatiya P. (2024). Room Temperature Based
SnO_2_/MoS_2_ Chemiresistive Sensor for Highly Selective
Detection of Acetone
for Disease Management through Simulated Gas Mixture Analysis. ACS Appl. Electron. Mater..

[288] Kim M., Park S., Ahn J., Baek J. W., Kim D. H., Shin H., Ko J., Song L., Park C., Shin E., Kim I. D. (2024). Vitalizing
Perovskite Oxide-Based
Acetone Sensors with Metal–Organic Framework-Derived Heterogeneous
Oxide Catalysts. ACS Sens..

[289] Xie J., Xie T., An C., Zhao Y., Wu J., Wu X. (2024). Amorphous Pt-Decorated
NiFe_2_O_4_ Nanorods for
Acetone Sensing. ACS Appl. Nano Mater..

[290] Zhang J., Shen H., Li Y., Wang L., Xie Z., Chen A., Zheng J., Miao S. (2025). High-Performance Acetone
Response in B-TiO_2_/SnS_2_ Heterojunction Nanosheets
Driven by Visible Light at Room Temperature. ACS Appl. Nano Mater..

[291] Zhang S., An Y., Pu Y., Cao S., Zhu D. (2025). MOFs-Derived Ti_0.5_Sn_0.5_O_2_ Nanoparticles
for Acetone Gas Detection with a ppb-Level Detection Limit. ACS Appl. Nano Mater..

[292] Mojumder S., Das T., Das S., Das S., Biswas M., Ghosh S., Pal M. (2025). Spinel Chromite MCr_2_O_4_ (M = Cu, Mg, Zn) Nanoparticle-Based Sensors
for Trace Acetone Detection and Noninvasive Diabetes Diagnosis from
Exhaled Breath. ACS Appl. Nano Mater..

[293] Zhang T., Wang P., Yang Y., Wang Y., Li R., An B., Wu Z., Han R., Xie E. (2025). Stoichiometric-Induced
Cationic Inversion of Cobalt Ferrite for High-Sensitivity Acetone
Gas Sensor and Mechanistic Insight. ACS Appl.
Electron. Mater..

[294] Li F., Yang B., Li J., Long Y., Zhang Z., Wang Z., Wang J., Chen G., Zhang Z., Yang R., Wang K., Zou W., Fang F., Zhang Y., Wang P., Zhan Z. (2025). Hard-Template Synthesis
of Inverse Opal Macroporous NiO-SnO_2_ Heterojunction for
Enhanced Acetone Detection. ACS Sens..

[295] Wang O., Ma Z., Xue Z., Yan M., An B. L., Zhao Y., Xu J., Wang X. (2025). Catalytic
Activation Function of Noble-Metal-Free High-Entropy Alloy for Enhancing
SnO_2_ Acetone Detection Capability. ACS Nano.

[296] Zhao L., Zhi S., Yu C., Xing Y., Zhang H., Fei T., Liu S., Zhang H., Zhang T. (2025). Modulation of the Surface Catalytic Activity of Boron-Doped Cobalt
Oxide with Crystalline/Amorphous Interfaces for High-Stability Acetone
Detection. ACS Sens..

[297] Wang T., Tao W., Kou X., Zhao L., Sun P., Lu G. (2024). Bi_2_Sn_2_O_7_ Overlayer
Assists Bilayer Chemiresistor in Humidity-Independent and Highly Selective
Detection of Expiratory Acetone. ACS Sens..

[298] Punginsang M., Chaemsai A., Inyawilert K., Siriwalai M., Wisitsoraat A., Liewhiran C. (2025). Performance
and Selectivity toward Acetone against Various Gaseous Markers for
Liver Diseases of Flame-Made Ir-Loaded In_2_O_3_ Nanoparticulate Sensors. ACS Sens..

[299] Lv L., Wang J., Guo L., Zhang C., Shi C., Xiao X., Yang S., Fan X., Yu L., Wang P., Li C., Zhou C. (2025). MOF-Derived
Vertically
Aligned Ce-Doped NiO Porous Nanowalls for Acetone Sensing. ACS Appl. Nano Mater..

[300] Hu J., Jin L., Kou H., Li H. J., Xu J., Hu Q., Li G., Wang D. (2025). Dual-Motor Oxygen-Deficiency-Engineering
Al-Doped W_18_O_49–x_ Circular Nanorod Arrays
for Parts per Billion-Level Acetone Detection. Nano Lett..

[301] Liu X., Li J., Wang H., Liu C., Tang Y., Zhu L., Alifu N., Dong B., Liu W. (2025). Macroporous ZnO Nanoreactors
Decorated with Amorphous CoO_x_ Clusters for Room Temperature
Sensors of Acetone in Breath. ACS Appl. Nano
Mater..

[302] Liao Z., Chu N., Hu Y., Yuan Z., Shen Y., Meng F. (2025). Performance and Mechanism Analysis
of Hexagonal Co­(OH)­F/Carbon Quantum Dots Composite Sensor Synthesized
by PVP-Assisted Method for Acetone Gas Detection. ACS Sens..

[303] Na E., Tao S., Wang W., Li J., Guo Y., Gao R., Li Q., Wang F., Zhang C., Li G. D. (2024). Ultrasensitive
Acetone Gas Sensor Based on a K/Sn–Co_3_O_4_ Porous Microsphere for Noninvasive Diabetes Diagnosis. ACS Sens..

[304] Das, A. ; Das, T. ; Parida, R. ; Kumar, A. ; Lee, J. Y. ; Pal, M. ; Dash, P. Trace Level Acetone Detection via a Schottky-Contacted SrFeO_3_–Ti_3_C_2_T_x_ Nanocomposite Sensor. ACS Appl. Nano Mater. 2025, 8. 19342 10.1021/acsanm.5c03341.

[305] Pargoletti E., Vertova A., Tricoli A., Starvaggi A., John A. T., Minelli S., Longhi M., Cappelletti G. (2025). Boosting Gaseous
Acetone Detection by Nanoheterojunctions of P-Type MWCNTs/PANI Integrated
into 3D Flame-Synthesized n-Type ZnO. ACS Sens..

[306] Li M., Guo Y., Guo Y., Li H., French P. J., Wang Y. (2025). Oxygen Vacancies Modulation
in CoFe_2_O_4_ via
Two-Step Incorporation of Synergistic TiO_2_/MXene and Carbon
for Enhanced Acetone Sensing. Adv. Mater. Technol..

[307] Wang H., Zhang H., Zhang J., Han J. P., Fang H., Yu M. H., Chang Z., Bu X. H. (2025). Innovative
Microstructure and Morphology Engineering of MOF-Derived Single-Atom
Sn-Doped ZnO Nanosheet Showing Highly Sensitive Acetone Sensing. Adv. Funct. Mater..

[308] Zhang S., An Y., Pu Y., Cao S., Zhu D. (2025). MOFs-Derived Ti_0.5_Sn_0.5_O_2_ Nanoparticles
for Acetone Gas Detection with a Ppb-Level Detection Limit. ACS Appl. Nano Mater..

[309] Weber I. C., Wang C. T., Güntner A. T. (2021). Room-Temperature
Catalyst Enables Selective Acetone Sensing. Materials.

[310] Weber I. C., Braun H. P., Krumeich F., Güntner A. T., Pratsinis S. E. (2020). Superior Acetone Selectivity in Gas Mixtures by Catalyst-Filtered
Chemoresistive Sensors. Adv. Sci..

[311] Zhu Y., Wu J., Zhang Z., Wu W., Wang X., Zhao Y., Zhao C. (2025). PtCu Nanoparticles
Sensitizing Mesoporous
In_2_O_3_ Hexagonal Hollow Nanotubes for Ppb-Level
Formaldehyde Evaluation in Aquatic Products. ACS Sens..

[312] Zhang S., Sun S., Huang B., Wang N., Li X. (2023). UV-Enhanced Formaldehyde Sensor Using Hollow In_2_O_3_@TiO_2_ Double-Layer Nanospheres at Room Temperature. ACS Appl. Mater. Interfaces.

[313] Wang G., Cai H., Wei J., Wang X., Zhang X., Ni T., Zhu Y. (2025). Controlled
Assembly
of Bimetallic PtRh-Modified Tin Oxide Hollow Nanotubes with High Sensing
Activity for Ultrasensitive Formaldehyde Detection. ACS Sens..

[314] Venkatraman M., Kadian A., Ganesan A., Dong C. L., Singh A., Dev K., Selvaraj M., Subramanian A., Marappan S. (2025). Ru-Doped CeO_2_ Nanoparticles
as Sensing Materials
for the Detection of Formaldehyde. ACS Appl.
Nano Mater..

[315] Han Q., Sui X., Jiao G., Ding Z., Zhang H., Zhang D. (2025). Ppb-Level Detection of a Formaldehyde Gas Sensor Based on the Honeycomb
SnO_2_/SnSe_2_ Heterostructure. ACS Appl. Electron. Mater..

[316] Zhang Y., Xu D., Zhou T., Song Z. L., Deng Z., Zi B., Zhang J., Zhao J., Liu Q., Hu G. (2023). Nonstoichiometric Doping
of La_0.9_Fe_x_S_n1–x_O_3_ Hollow Microspheres for
an Ultrasensitive Formaldehyde Sensor. ACS Sens..

[317] Qin C., Zhang Y., Wang Y., Zhang Y., Cao J. (2023). Nanoporous
Mixed-Phase In_2_O_3_ Nanoparticle Homojunctions
for Formaldehyde Sensing. ACS Appl. Nano Mater..

[318] D’Andria M., Krumeich F., Yao Z., Wang F. R., Güntner A. T. (2024). Structure-Function
Relationship of Highly Reactive
CuO_x_ Clusters on Co_3_O_4_ for Selective
Formaldehyde Sensing at Low Temperatures. Adv.
Sci..

[319] Ma X., Chen D., Li W., He L., Jin L., Zhang J., Zhang K. (2025). PEI Doped In_2_O_3_ Nanospheres Based Gas Sensor for High-Performance Formaldehyde
Detection. Sens. Actuators, B.

[320] Shuai Y., Peng W., Tang Q., Shen W. (2025). High-Performance
Formaldehyde Gas Sensor Based on Flower-like ZnCo_2_O_4_/In_2_O_3_ Heterostructures. Sens. Actuators, B.

[321] Xiao C., Zhang X., Ma Z., Yang K., Gao X., Wang H., Jia L. (2022). Formaldehyde Gas Sensor with 1 Ppb
Detection Limit Based on In-Doped LaFeO_3_ Porous Structure. Sens. Actuators, B.

[322] Chu N., Wang Z., Gu F. (2025). Oxygen Vacancies Enabled
MOF-Derived
Tb–SnO_2_ Compound for a High-Response, Low Detection
Limit, and Humidity-Tolerant Chemiresistive Gas Sensor of Formaldehyde. ACS Appl. Electron. Mater..

[323] Li W., Yuan Q., Xia Z., Ma X., He L., Jin L., Chu X., Zhang K. (2024). ZnO Quantum
Dots Sensitized ZnSnO_3_ for Highly Formaldehyde Sensing
at a Low Temperature. Sens. Actuators, B.

[324] Deng Z., Zhang Y., Xu D., Zi B., Zeng J., Lu Q., Xiong K., Zhang J., Zhao J., Liu Q. (2022). Ultrasensitive Formaldehyde Sensor
Based on SnO_2_ with Rich Adsorbed Oxygen Derived from a
Metal Organic Framework. ACS Sens..

[325] Huang J., Ma Z., Li J., Zhang Z., Tang J., Cao X., Xu W., Zhao X., Yang Y., Pan X., Du Y. (2023). Au Nanocage/In_2_O_3_ Nanoparticle-Based Hybrid Structures for Formaldehyde
Sensors. ACS Appl. Nano Mater..

[326] Adhikari M., Saha D., Chattopadhyay D., Pal M. (2025). Rationally Designed Polypyrrole-Encapsulated MoO_3_ Hollow
Nanostructures for Enhanced Formaldehyde Detection at Room Temperature. ACS Appl. Eng. Mater..

[327] He Z.-K., Zhao J., Li K., Zhao J., He H., Gao Z., Song Y.-Y. (2023). Rational
Integration of SnMOF/SnO_2_ Hybrid on TiO_2_ Nanotube
Arrays: An Effective Strategy
for Accelerating Formaldehyde Sensing Performance at Room Temperature. ACS Sens..

[328] Nakate U. T., Yu Y. T., Park S. (2021). High Performance
Acetaldehyde
Gas Sensor Based on P-n Heterojunction Interface of NiO Nanosheets
and WO_3_ Nanorods. Sens. Actuators,
B.

[329] Zhang S., Pu Y., Cao S., Zhu D. (2023). SnO_2_ Nanoparticles Derived from Metal–Organic Precursors
as an
Acetaldehyde Gas Sensor with Ppb-Level Detection Limit. ACS Appl. Nano Mater..

[330] Li Q., He R., Feng F., Jiang C., Song S., Peng H., Tang X. (2024). Copper Oxide/Reduced
Graphene Oxide/Graphene
Composite Structure for the Chemiresistive Detection of Acetaldehyde
at Room Temperature. Sens. Actuators, B.

[331] Jin L., Yang K., Chen L., Yan R., He L., Ye M., Qiao H., Chu X., Gao H., Zhang K. (2023). Flexible Synergistic
MoS_2_ Quantum Dots/PEDOT: PSS Film Sensor for Acetaldehyde
Sensing at Room Temperature. Anal. Chem..

[332] Zhou K., Yang H., Du Z., Yang Y., Zhu C., Su H., Dong W., Zeng D. (2025). Ultrahigh Selectivity
H_2_S Gas Sensor Based CsPbBr_3_ Perovskites via
Pb–S Bonding Interaction. ACS Sens..

[333] Zheng M., Cheng Y., Zhang X., Liu H., Xu H., Dai X., Shi G., Rao Y., Gu L., Wang M.-S., Li C., Li K. (2025). Atomic Ru Species Driven
SnO_2_-Based Sensor for Highly Sensitive and Selective Detection
of H_2_S in the Ppb-Level. ACS Sens..

[334] Zafar Z., Li J.-P., Rabbani M. S., Hou X., Yue X.-Z. (2025). Ag Nanoparticle-Decorated
Spindle-Shaped BiVO_4_Nanorods for H_2_S Detection. ACS
Appl. Nano Mater..

[335] Song X., Pan Z., Wang Z., Huang W., Xing J. (2024). Metal–Organic Framework-Derived
Porous Hollow Co_3_O_4_/ZnO Nanofibers for H_2_S Sensing. ACS Appl. Nano Mater..

[336] Lee J. S., Lee S., Seo J. W., Choi S. H., Kim S. J., Choi S. J. (2025). Porous
One-Dimensional CuWO_4_-WO_3_ Nanofibers with Enhanced
Gas Accessibility and Catalytic
Sensitization for Selective H_2_S Sensors. Sens. Actuators, B.

[337] Tan W., Zhao L., Li F. fei., Cui G., Zhang P., Shi C. (2025). Low-Temperature-Enhanced and Ultrasensitive
Exhaled H_2_S Gas Sensor Based on Cu_2_O-CuFe_2_O_4_ Nanoarrays. ACS Sens..

[338] Qiu C., Zhu L., Li Q., Wang H., An F., Wang S., Fan C., Zhang D., Yang Z. (2025). One-Step Electrospun
WO_3_/CuO p–n Heterojunction Nanocomposites for Ultrasensitive
and Rapid H_2_S Detection. ACS Sens..

[339] Xue H., Huang F., He X., Yu Z., Zhang T., Zhu G., Xia C. (2025). Hierarchical Porous CdS–Co_3_O_4_ Heterostructures for H_2_S Gas Sensing at Room Temperature. ACS Appl. Nano Mater..

[340] Qiu C., Zhang H., Li Q., Song Y., An F., Wang H., Wang S., Zhu L., Zhang D., Yang Z. (2024). High Performance H_2_S Sensor
Based on Ordered Fe_2_O_3_/Ti_3_C_2_ Nanostructure at Room Temperature. ACS Sens..

[341] Deb M., Lu C. J., Zan H. W. (2024). Achieving Room-Temperature Ppb-Level
H_2_S Detection in a Au-SnO_2_ Sensor with Low Voltage
Enhancement Effect. ACS Sens..

[342] Ruksana S., Rajbhar M. K., Das B., Sharma C. S., Kumar M. (2024). MoSe_2_-Layered Nanosheet
Decorated SnO_2_ Hollow
Nanofiber-Based Highly Sensitive and Selective Room Temperature H_2_S Gas Sensor. ACS Appl. Mater. Interfaces.

[343] Zhu L., Rong Q., Yang Z., Zhang W., Jiao M., Song J., Wang C., Guo Y. (2022). ZnO Nanoparticle-Based
MEMS Sensors for H_2_S Detection. ACS
Appl. Nano Mater..

[344] Wang Y., Li J., Zhang D., Zhou T., Sun M., Chen S., Sun M. (2025). Low Temperature
Part Per Billion
H_2_S Sensor Based on P–N CuO–WO_3_ Heterostructured Microflowers. ACS Sens..

[345] Zhao W., Yao G., Wu H., Liu Y., Zhu H., Huang Z., Chen W., Liu H., Li X., Na J., Qin K., Yu J. (2025). Chemiresistive Room Temperature H_2_S Sensor
Based on Cu_n_O Nanoflowers Fabricated by
Laser Ablation. Sens. Actuators, B.

[346] Bai H., Feng C., Chen Y., Yan Y., Feng Y., Liu K., Zhang B., Wang J., Chen D., Zheng Y., Guo F. (2024). Chemiresistive Room
Temperature H_2_S Gas Sensor Based on
MoO_3_ Nanobelts Decorated with MnO_2_ Nanoparticles. Sens. Actuators, B.

[347] Shen Y., Lv Y., Zhuo Q. (2024). Extremely Flexible
Methanol Sensor Based on CNFs/NiCo_2_O_4_ Nanocomposite. ACS Appl. Nano Mater..

[348] Rong Q., Xiao B., Zeng J., Yu R., Zi B., Zhang G., Zhu Z., Zhang J., Wu J., Liu Q. (2022). Pt Single Atom-Induced Activation Energy and Adsorption
Enhancement
for an Ultrasensitive Ppb-Level Methanol Gas Sensor. ACS Sens..

[349] Liu M., Wang Z., Song P., Yang Z., Wang Q. (2021). In_2_O_3_ Nanocubes/Ti_3_C_2_T_x_ MXene
Composites for Enhanced Methanol Gas Sensing Properties at Room Temperature. Ceram. Int..

[350] Seekaew Y., Wisitsoraat A., Wongchoosuk C. (2023). ZnO Quantum
Dots Decorated Carbon Nanotubes-Based Sensors for Methanol Detection
at Room Temperature. Diam. Relat. Mater..

[351] Jeong S. Y., Jang J. (2025). CeO_2_-Filter-Based
Monolithic
Bilayer Gas Sensors for Selective, Sensitive, and Fast Methanol Detection. Chem. Eng. J..

[352] Wang Z., Jiang Y., Chang X., Li J., Zhu X., Gao W., Zhang Y., Wang D., Sun S. (2026). Ultra-Sensitive
and Selective Methanol Sensor Based on Crystallinity-Engineered LaFeO_3_ Porous Nanoarchitectures. Sens. Actuators,
B.

[353] Van Den Broek J., Bischof D., Derron N., Abegg S., Gerber P. A., Güntner A. T., Pratsinis S. E. (2021). Screening
Methanol Poisoning with a Portable Breath Detector. Anal. Chem..

[354] Yin L., Yu T., Liu J., Guo W., Liu C., Song H., Li X. (2025). Flower-like MoS_2_-Induced
α-Fe_2_O_3_/Fe_2_(MoO_4_)_3_ Nanocomposite as an Ultrafast Sensor for Ethanol Detection. Langmuir.

[355] Wang S. K., Wang A. C., Zhang C. Y., Liu Q. Y., Cheng J. Di., Wang Y. C., Gao X. P., Xie Q. F., Zhang Z. X., Sun G. Z., Pan X. J., Zhou J. Y. (2023). Sandwich-Structured
In_2_S_3_/In_2_O_3_/In_2_S_3_ Hollow Nanofibers as Sensing Materials for Ethanol
Detection. ACS Appl. Nano Mater..

[356] Chu Y. L., Young S. J., Huang Y. R., Arya S., Chu T. Te. (2025). Highly Sensitive Ethanol Gas Sensors
of Au Nanoparticle-Adsorbed
ZnO Nanorod Arrays via a Photochemical Deposition Treatment. ACS Appl. Electron. Mater..

[357] Jiang B., Zhou T., Zhang L., Han W., Yang J., Wang C., Sun Y., Liu F., Sun P., Lu G. (2023). Construction of Mesoporous In_2_O_3_-ZnO Hierarchical Structure Gas Sensor for Ethanol Detection. Sens. Actuators, B.

[358] Wang D., Zhang D., Mi Q. (2022). A High-Performance
Room Temperature Benzene Gas Sensor Based on CoTiO_3_ Covered
TiO_2_ Nanospheres Decorated with Pd Nanoparticles. Sens. Actuators, B.

[359] Kim K. B., Moon Y. K., Kim T. H., Yu B. H., Li H. Y., Kang Y. C., Yoon J. W. (2023). Highly Selective
and Sensitive Detection of Carcinogenic Benzene Using a Raisin Bread-Structured
Film Comprising Catalytic Pd-Co_3_O_4_ and Gas-Sensing
SnO_2_ Hollow Spheres. Sens. Actuators,
B.

[360] Manikandan V., Kadian A., Dev K., Annapoorni S. (2024). Room Temperature
Benzene Gas Sensing Properties Based on Sr-Substituted Ceria Oxide
Nanopetals. J. Environ. Chem. Eng..

[361] Yang T. Y., Yu G. X., Liu J., Li X., Chen L., Guo Z. (2025). Bimetallic Au-Pt Nanoparticle-Supported
ZnO Porous Nanobelts for Selective Gas Sensing Enhancement to Benzene. Sens. Actuators, B.

[362] Weber I. C., Rüedi P., Šot P., Güntner A. T., Pratsinis S. E. (2022). Handheld Device for Selective Benzene
Sensing over Toluene and Xylene. Adv. Sci..

[363] D’Andria M., Elias Abi-Ramia Silva T., Consogno E., Krumeich F., Güntner A. T. (2024). Metastable
CoCu_2_O_3_ Nanocrystals from Combustion-Aerosols
for Molecular Sensing
and Catalysis. Adv. Mater..

[364] Paciencia F. C., Theodoro R. d. S., Santos G. S. M., Perfecto T. M., Volanti D. P. (2025). Micrometric Co_3_O_4_/ZIF-67 with
High Toluene Detection Capability. Mater. Sci.
Semicond. Process..

[365] Shen Z., Guo X., Chai W., Jin D., Jin H. (2025). Mesoporous SnO_2_@Co_3_O_4_ Core–Shell
Nanospheres for High-Performance Toluene Gas Sensors. ACS Appl. Nano Mater..

[366] Hong J., Chen K., Yuan K., Lin M., Cao Q., Lin J., Wang X., Bai X., Ishida T., Murayama T., Xiu G. (2025). Detection of Toluene
at Ppb Levels
Using Au Catalyst Supported on Al-Doped ZnO. J. Environ. Chem. Eng..

[367] Zhang H., Hu J., Li M., Li Z., Yuan Y., Yang X., Guo L. (2021). Highly Efficient Toluene
Gas Sensor Based on Spinel Structured Hollow Urchin-like Core-Shell
ZnFe_2_O_4_ Spheres. Sens.
Actuators, B.

[368] Yang H., Zhao M. M., Li L., Zhang L. X. (2022). ZnO Nanorods
with Doubly Positive Oxygen Vacancies for Efficient Xylene Sensing. ACS Appl. Nano Mater..

[369] Theodoro R. d. S., Sanghikian Marques dos
Santos G., de Sá B. S., Perfecto T. M., Volanti D. P. (2024). Multiple-Yolk–Shell
NiO Microspheres for Selective Detection of m-Xylene. ACS Appl. Mater. Interfaces.

[370] Zhao C., Dong Z., Pan S., Wu X., Tang X. (2024). Surface Modification
of CuFe_2_O_4_ Nanotubes with
Well-Dispersive Au Nanoparticles for Sensitive and Selective Xylene
Gas Detection. ACS Appl. Nano Mater..

[371] Verma M., Bahuguna G., Saharan A., Gaur S., Haick H., Gupta R. (2023). Room Temperature Humidity Tolerant
Xylene Sensor Using a Sn-SnO_2_ Nanocomposite. ACS Appl. Mater. Interfaces.

[372] Yang W., Fang B., Zhang Y., Ma G., Meng H., Liu S. (2025). Chemiresistive Detection of Xylene
Vapor Using MOF-Derived Porous Co_3_O_4_ Microrods
Activated by Mo^6+^ Cations. Sens.
Actuators, B.

[373] Neethirajan S., Jayas D. S., Sadistap S. (2009). Carbon Dioxide (CO_2_) Sensors
for the Agri-Food Industry—A Review. Food Bioprocess Technol..

[374] Hübner M., Simion C. E., Tomescu-Stǎnoiu A., Pokhrel S., Bârsan N., Weimar U. (2011). Influence of Humidity
on CO Sensing with P-Type CuO Thick Film Gas Sensors. Sens. Actuators, B.

[375] Xie C., Yan D., Li H., Du S., Chen W., Wang Y., Zou Y., Chen R., Wang S. (2020). Defect Chemistry
in Heterogeneous Catalysis: Recognition, Understanding, and Utilization. ACS Catal..

[376] Jia Y., Jiang K., Wang H., Yao X. (2019). The Role of Defect
Sites in Nanomaterials for Electrocatalytic Energy Conversion. Chem.

[377] Yamazoe N., Shimanoe K. (2009). Receptor Function and Response of
Semiconductor Gas Sensor. J. Sens..

[378] Ashcroft, N. W. ; Mermin, N. D. Solid State Physics; Holt, Rinehart and Winston: New York, 1976.

[379] Fujio Y., Ayu Anggraini S., Bârsan N., Weimar U. (2003). Understanding the Fundamental
Principles of Metal Oxidebased
Gas Sensors; the Example of CO Sensing with SnO2 Sensors in the Presence
of Humidity. J. Phys.: Condens. Matter.

[380] Oprea A., Bârsan N., Weimar U. (2009). Work Function Changes
in Gas Sensitive Materials: Fundamentals and Applications. Sens. Actuators, B.

[381] Norsko J. K. (1990). Chemisorption on Metal Surfaces. Rep. Prog. Phys..

[382] Bârsan N., Huebner M., Weimar U. (2020). Conduction
Mechanism
in Semiconducting Metal Oxide Sensing Films: Impact on Transduction. Semicond. Gas Sens..

[383] Bârsan N., Hübner M., Weimar U. (2011). Conduction Mechanisms
in SnO_2_ Based Polycrystalline Thick Film Gas Sensors Exposed
to CO and H_2_ in Different Oxygen Backgrounds. Sens. Actuators, B.

[384] Bordiga S., Groppo E., Agostini G., Van Bokhoven J. A., Lamberti C. (2013). Reactivity of Surface Species in
Heterogeneous Catalysts
Probed by In Situ X-Ray Absorption Techniques. Chem. Rev..

[385] Piliai L., Dinhová T. N., Janata M., Balakin D., Vallejos S., Otta J., Štefková J., Fišer L., Fitl P., Novotný M., Hubálek J., Vorochta M., Matolinová I., Vrňata M. (2023). NAP-XPS Study
of Surface Chemistry of CO and Ethanol
Sensing with WO3 Nanowires-Based Gas Sensor. Sens. Actuators, B.

[386] Kucharski S., Ferrer P., Venturini F., Held G., Walton A. S., Byrne C., Covington J. A., Ayyala S. K., Beale A. M., Blackman C. (2022). Direct in Situ Spectroscopic
Evidence of the Crucial Role Played by Surface Oxygen Vacancies in
the O2-Sensing Mechanism of SnO2. Chem. Sci..

[387] Kucharski S., Vorochta M., Piliai L., Beale A. M., Blackman C. (2025). Interplay
between CO and Surface Lattice Oxygen Ions
in the Vacancy-Mediated Response Mechanism of SnO2-Based Gas Sensors. ACS Sens..

[388] Gurlo A. (2006). Interplay between O_2_ and
SnO_2_: Oxygen Ionosorption
and Spectroscopic Evidence for Adsorbed Oxygen. ChemPhysChem.

[389] Barsan N., Weimar U. (2001). Conduction Model of Metal Oxide Gas
Sensors. J. Electroceram..

[390] Weisz P. B. (1953). Effects of Electronic Charge Transfer
between Adsorbate
and Solid on Chemisorption and Catalysis. J.
Chem. Phys..

[391] Ruiz
Puigdollers A., Schlexer P., Tosoni S., Pacchioni G. (2017). Increasing
Oxide Reducibility: The Role of Metal/Oxide Interfaces in the Formation
of Oxygen Vacancies. ACS Catal..

[392] Blackman C. (2021). Do We Need “Ionosorbed”
Oxygen Species?
(Or, “A Surface Conductivity Model of Gas Sensitivity in Metal
Oxides Based on Variable Surface Oxygen Vacancy Concentration”). ACS Sens..

[393] Jovic V., Moser S., Papadogianni A., Koch R. J., Rossi A., Jozwiak C., Bostwick A., Rotenberg E., Kennedy J. V., Bierwagen O., Smith K. E. (2020). The Itinerant 2D Electron Gas of the Indium Oxide (111)
Surface: Implications for Carbon- and Energy-Conversion Applications. Small.

[394] Zhou P., Tsang J.-H., Blackman C., Shen Y., Liang J., Covington J. A., Saffell J., Danesh E. (2024). A Novel Mechanism
Based on Oxygen Vacancies to Describe Isobutylene and Ammonia Sensing
of P-Type Cr_2_O_3_ and Ti-Doped Cr_2_O_3_ Thin Films. Chemosensors.

[395] Yamazoe N. (1991). New Approaches for Improving Semiconductor
Gas Sensors. Sens. Actuators, B.

[396] Matsushima S., Teraoka Y., Miura N., Yamazoe N. (1988). Electronic
Interaction between Metal Additives and Tin Dioxide in Tin Dioxide-Based
Gas Sensors. Jpn. J. Appl. Phys..

[397] Morrison S. R. (1987). Selectivity in Semiconductor Gas
Sensors. Sens. Actuators.

[398] Chen H., Zhao Y., Shi L., Li G.-D., Sun L., Zou X. (2018). Revealing the Relationship
between Energy Level and
Gas Sensing Performance in Heteroatom-Doped Semiconducting Nanostructures. ACS Appl. Mater. Interfaces.

[399] Liu Y., Xiao S., Du K. (2021). Chemiresistive
Gas Sensors Based
on Hollow Heterojunction: A Review. Adv. Mater.
Interfaces.

[400] Kang L., Wang B., Bing Q., Zalibera M., Büchel R., Xu R., Wang Q., Liu Y., Gianolio D., Tang C. C., Gibson E. K., Danaie M., Allen C., Wu K., Marlow S., Sun L., He Q., Guan S., Savitsky A., Velasco-Vélez J. J., Callison J., Kay C. W. M., Pratsinis S. E., Lubitz W., Liu J., Wang F. R. (2020). Adsorption and Activation
of Molecular Oxygen over Atomic Copper­(I/II) Site on Ceria. Nat. Commun..

[401] Lappalainen J., Tuller H. L., Lantto V. (2004). Electronic Conductivity
and Dielectric Properties of Nanocrystalline CeO_2_ Films. J. Electroceram..

[402] Montini T., Melchionna M., Monai M., Fornasiero P. (2016). Fundamentals
and Catalytic Applications of CeO_2_-Based Materials. Chem. Rev..

[403] Recum P., Hirsch T. (2023). Graphene-Based Chemiresistive
Gas
Sensors. Nanoscale Adv..

[404] Nørskov J. K., Abild-Pedersen F., Studt F., Bligaard T. (2011). Density Functional
Theory in Surface Chemistry and Catalysis. Proc.
Natl. Acad. Sci. U.S.A..

[405] Yamazoe N., Fuchigami J., Kishikawa M., Seiyama T. (1979). Interactions of Tin Oxide Surface
with O_2_, H_2_O AND H_2_. Surf. Sci..

[406] Chu T., Rong C., Zhou L., Mao X., Zhang B., Xuan F. (2023). Progress and Perspectives of Single-Atom
Catalysts for Gas Sensing. Adv. Mater..

[407] Giulimondi V., Mitchell S., Pérez-Ramírez J. (2023). Challenges
and Opportunities in Engineering the Electronic Structure of Single-Atom
Catalysts. ACS Catal..

[408] Greiner M. T., Jones T. E., Beeg S., Zwiener L., Scherzer M., Girgsdies F., Piccinin S., Armbrüster M., Knop-Gericke A., Schlögl R. (2018). Free-Atom-like d States in Single-Atom
Alloy Catalysts. Nat. Chem..

[409] Lee B.-H., Park S., Kim M., Sinha A. K., Lee S. C., Jung E., Chang W. J., Lee K.-S., Kim J. H., Cho S.-P., Kim H., Nam K. T., Hyeon T. (2019). Reversible and Cooperative Photoactivation
of Single-Atom Cu/TiO_2_ Photocatalysts. Nat. Mater..

[410] Kaiser S. K., Chen Z., Faust Akl D., Mitchell S., Pérez-Ramírez J. (2020). Single-Atom
Catalysts across the Periodic Table. Chem. Rev..

[411] Lai W., Miao Z., Wang Y., Wang J., Chou S. (2019). Atomic-Local
Environments of Single-Atom Catalysts: Synthesis, Electronic Structure,
and Activity. Adv. Energy Mater..

[412] Yamazoe, N. ; Shimanoe, K. Fundamentals of Semiconductor Gas Sensors. In Semiconductor Gas Sensors; Elsevier, 2020; pp 3–38 10.1016/B978-0-08-102559-8.00001-X.

[413] Duan X., Zhang H. (2024). Introduction: Two-Dimensional
Layered
Transition Metal Dichalcogenides. Chem. Rev..

[414] Llobet E. (2024). Transition
Metal Dichalcogenide Based Toxic Gas Sensing. Curr. Opin. Environ. Sci. Health.

[415] Ashraf I., Rizwan S., Iqbal M. (2020). A Comprehensive
Review
on the Synthesis and Energy Applications of Nano-Structured Metal
Nitrides. Front. Mater..

[416] Sharma N., Pandey V., Gupta A., Tan S. T., Tripathy S., Kumar M. (2022). Recent Progress on
Group III Nitride
Nanostructure-Based Gas Sensors. J. Mater. Chem.
C.

[417] Tang W., Xing G., Xu X., Chen B. (2024). Emerging Hybrid
Metal Halide Glasses for Sensing and Displays. Sensors.

[418] Lee D., Lee S., Kim J. H., Park J., Kang Y., Song M., Lee H. W., Kim H. S., Choi J. W. (2022). Multimodal
Gas Sensor Detecting Hydroxyl Groups with Phase Transition Based on
Eco-Friendly Lead-Free Metal Halides. Adv. Funct.
Mater..

[419] Shinde P. V., Patra A., Rout C. S. (2022). A Review
on the
Sensing Mechanisms and Recent Developments on Metal Halide-Based Perovskite
Gas Sensors. J. Mater. Chem. C.

[420] Liu H., Bansal S. (2024). Pt and Pt-group Transition Metal 0D Vacancy Ordered
Halide Perovskites: A Review. EcoMat.

[421] Zhao X., Xu Z., Zhang Z., Liu J., Yan X., Zhu Y., Attfield J. P., Yang M. (2025). Titanium Nitride Sensor
for Selective NO_2_ Detection. Nat.
Commun..

[422] Gu H., Zhang T., Wang Y., Zhou T., Chen H. (2024). 2D Compounds
with Heterolayered Architecture for Infrared Photodetectors. Chem. Sci..

[423] de Sousa Silva L., Fileti E. E., Colherinhas G. (2025). Exploring
MXene Materials in Energy Storage Devices: A Review of Supercapacitor
Applications. ACS Mater. Au.

[424] Cai Z., Kim H. (2025). Recent Advances in
MXene Gas Sensors: Synthesis, Composites,
and Mechanisms. NPJ 2D Mater. Appl..

[425] Paul T. K., Khaleque M. A., Ali M. R., Aly Saad
Aly M., Bacchu Md. S., Rahman S., Khan Md. Z. H. (2025). MXenes from
MAX Phases: Synthesis, Hybridization, and Advances in Supercapacitor
Applications. RSC Adv..

[426] Anasori B., Lukatskaya M. R., Gogotsi Y. (2017). 2D Metal Carbides and
Nitrides (MXenes) for Energy Storage. Nat. Rev.
Mater..

[427] Khanal R., Irle S. (2023). Effect of Surface Functional Groups
on MXene Conductivity. J. Chem. Phys..

[428] Márquez F. (2025). MXenes in Solid-State Batteries:
Multifunctional Roles
from Electrodes to Electrolytes and Interfacial Engineering. Batteries.

[429] Devaraj M., Rajendran S., Hoang T. K. A., Soto-Moscoso M. (2022). A Review on
MXene and Its Nanocomposites for the Detection of Toxic Inorganic
Gases. Chemosphere.

[430] Liu Z., He T., Sun H., Huang B., Li X. (2022). Layered MXene
Heterostructured with In_2_O_3_ Nanoparticles for
Ammonia Sensors at Room Temperature. Sens. Actuators,
B.

[431] Kitagawa S., Kitaura R., Noro S. (2004). Functional
Porous Coordination
Polymers. Angew. Chem., Int. Ed..

[432] Zhu Q.-L., Xu Q. (2014). Metal–Organic Framework Composites. Chem. Soc. Rev..

[433] Long J. R., Yaghi O. M. (2009). The Pervasive Chemistry
of Metal–Organic
Frameworks. Chem. Soc. Rev..

[434] Santos G. S. M., de Sá B. S., Perfecto T. M., Volanti D. P. (2024). MOF-Derived
Co_3_O_4_-ZnO Heterostructure for 3-Methyl-1-Butanol
Detection. Sens. Actuators, B.

[435] Xia W., Mahmood A., Zou R., Xu Q. (2015). Metal–Organic
Frameworks and Their Derived Nanostructures for Electrochemical Energy
Storage and Conversion. Energy Environ. Sci..

[436] Suresh K., Carey C. A., Matzger A. J. (2024). Metal–Organic
Frameworks (MOFs) Morphology Control: Recent Progress and Challenges. Cryst. Growth Des..

[437] Ming Y., Purewal J., Liu D., Sudik A., Xu C., Yang J., Veenstra M., Rhodes K., Soltis R., Warner J., Gaab M., Müller U., Siegel D. J. (2014). Thermophysical Properties of MOF-5
Powders. Microporous Mesoporous Mater..

[438] Purewal J., Liu D., Sudik A., Veenstra M., Yang J., Maurer S., Müller U., Siegel D. J. (2012). Improved Hydrogen Storage and Thermal Conductivity
in High-Density MOF-5 Composites. J. Phys. Chem.
C.

[439] Bhuyan A., Ahmaruzzaman Md. (2024). Recent Advances in MOF-5-Based Photocatalysts
for Efficient Degradation of Toxic Organic Dyes in Aqueous Medium. Next Sustainability.

[440] Wang H., Pei X., Kalmutzki M. J., Yang J., Yaghi O. M. (2022). Large Cages of Zeolitic Imidazolate
Frameworks. Acc. Chem. Res..

[441] Zheng Z., Rong Z., Nguyen H. L., Yaghi O. M. (2023). Structural
Chemistry of Zeolitic Imidazolate Frameworks. Inorg. Chem..

[442] Ministry of Ecology, S. D. T. and H. . Decree No. 2011–1727 of December 2, 2011, Concerning Indoor Air Guideline Values for Formaldehyde and Benzene; Paris, 2011.

[443] Zhao F., Cao W., Wang P.-H., Wang J., Yu L., Qiao Z., Ding Z.-J. (2023). Fast and
Sensitive Detection of CO
by Bi-MOF-Derived Porous In_2_O_3_/Fe_2_O_3_ Core–Shell Nanotubes. ACS Sens..

[444] Barrett E. P., Joyner L. G., Halenda P. P. (1951). The Determination
of Pore Volume and Area Distributions in Porous Substances. I. Computations
from Nitrogen Isotherms. J. Am. Chem. Soc..

[445] Groen J. C., Peffer L. A. A., Pérez-Ramírez J. (2003). Pore Size
Determination in Modified Micro- and Mesoporous Materials. Pitfalls
and Limitations in Gas Adsorption Data Analysis. Microporous Mesoporous Mater..

[446] Kenvin J., Mitchell S., Sterling M., Warringham R., Keller T. C., Crivelli P., Jagiello J., Pérez-Ramírez J. (2016). Quantifying
the Complex Pore Architecture of Hierarchical Faujasite Zeolites and
the Impact on Diffusion. Adv. Funct. Mater..

[447] Šolcová O., Matějová L., Topka P., Musilová Z., Schneider P. (2011). Comparison
of Textural Information from Argon­(87 K) and Nitrogen­(77 K) Physisorption. J. Porous Mater..

[448] Thommes M., Köhn R., Fröba M. (2000). Sorption and
Pore Condensation Behavior of Nitrogen, Argon, and Krypton in Mesoporous
MCM-48 Silica Materials. J. Phys. Chem. B.

[449] Majhi S.
M., Mirzaei A., Kim H. W., Kim S. S. (2021). Reduced
Graphene Oxide (RGO)-Loaded Metal-Oxide Nanofiber Gas Sensors: An
Overview. Sensors.

[450] Zhang W.-D., Zhang W.-H. (2009). Carbon Nanotubes
as Active Components
for Gas Sensors. J. Sens..

[451] Dronskowski R., Bloechl P. E. (1993). Crystal Orbital Hamilton Populations
(COHP): Energy-Resolved Visualization of Chemical Bonding in Solids
Based on Density-Functional Calculations. J.
Phys. Chem. A.

[452] Yue Y., Yu M., Yao Z., Fang G., Wang B., Wang S., Jin C., Chang R., Sun T., Pan Z., Zhu Y., Wang F. R., Li X., Zhao J. (2025). Copper Integrative
Catalytic Pairs with Mixed-Valence Cu^2+^-Cu^3+^ Species for Selective Alkyne Conversion. Nat.
Commun..

[453] Song H. W., Choi W., Jeon T., Oh J. H. (2023). Recent
Advances in Smart Organic Sensors for Environmental Monitoring Systems. ACS Appl. Electron. Mater..

[454] Verma A., Gupta R., Verma A. S., Kumar T. (2023). A Review of
Composite Conducting Polymer-Based Sensors for Detection of Industrial
Waste Gases. Sens. Actuators Rep..

[455] Abdelhamid H. N. (2025). Organic-Based Nanostructure for Chemosensors
and Biosensors:
A Review. Microchem. J..

[456] Xu L., Liu C., Ma X., Xu Y., Zhou W., Guan W., Qiang Q., Lang T., Peng L., Zhong Y., Alexey Nikolaevich Y., Zhou Z., Liu B. (2023). Two-Birds-One-Stone:
Flexible PANI Film with Bionic Microstructures for Multifunctional
Sensing of Physical and Chemical Stimuli. Chem.
Eng. J..

[457] Pang S., Wang Z., Yuan X., Pan L., Deng W., Tang H., Wu H., Chen S., Duan C., Huang F., Cao Y. (2021). A Facile Synthesized
Polymer Featuring B-N Covalent Bond and Small Singlet-Triplet Gap
for High-Performance Organic Solar Cells. Angew.
Chem., Int. Ed..

[458] Kwak D., Lei Y., Maric R. (2019). Ammonia Gas
Sensors:
A Comprehensive Review. Talanta.

[459] Hänninen O. O., Alm S., Katsouyanni K., Künzli N., Maroni M., Nieuwenhuijsen M. J., Saarela K., Srám R. J., Zmirou D., Jantunen M. J. (2004). The EXPOLIS
Study: Implications for Exposure Research and Environmental Policy
in Europe. J. Exposure Sci. Environ. Epidemiol..

[460] Graf M., Frey U., Taschini S., Hierlemann A. (2006). Micro Hot
Plate-Based Sensor Array System for the Detection of Environmentally
Relevant Gases. Anal. Chem..

[461] Abegg S., Klein Cerrejon D., Güntner A. T., Pratsinis S. E. (2020). Thickness Optimization of Highly
Porous Flame-Aerosol
Deposited WO_3_ Films for NO_2_ Sensing at Ppb. Nanomaterials.

[462] Belmonte J. C., Puigcorbé J., Arbiol J., Vilà A., Morante J. R., Sabaté N., Gràcia I., Cané C. (2006). High-Temperature Low-Power Performing Micromachined
Suspended Micro-Hotplate for Gas Sensing Applications. Sens. Actuators, B.

[463] Spruit R. G., van Omme J. T., Ghatkesar M. K., Garza H. H. P. (2017). A Review on Development and Optimization of Microheaters
for High-Temperature In Situ Studies. J. Microelectromech.
Syst..

[464] Liu H., Zhang L., Li K. H. H., Tan O. K. (2018). Microhotplates for
Metal Oxide Semiconductor Gas Sensor Applications—Towards the
CMOS-MEMS Monolithic Approach. Micromachines.

[465] Huang S., Shen R., Qian B., Li L., Wang W., Lin G., Zhang X., Li P., Xie Y. (2018). Thermal Bubble Inkjet Printing of Water-Based Graphene Oxide and
Graphene Inks on Heated Substrate. J. Phys.
D: Appl. Phys..

[466] Matavž A., Malič B. (2018). Inkjet Printing
of Functional Oxide
Nanostructures from Solution-Based Inks. J.
Sol-Gel Sci. Technol..

[467] Gelderblom H., Diddens C., Marin A. (2022). Evaporation-Driven
Liquid Flow in Sessile Droplets. Soft Matter.

[468] Mädler L., Roessler A., Pratsinis S. E., Sahm T., Gurlo A., Barsan N., Weimar U. (2006). Direct Formation
of Highly Porous Gas-Sensing Films by in Situ Thermophoretic Deposition
of Flame-Made Pt/SnO2 Nanoparticles. Sens. Actuators,
B.

[469] Daneshian B., Gärtner F., Assadi H., Vidaller M. V., Höche D., Klassen T. (2022). Features of Ceramic Nanoparticle
Deformation in Aerosol Deposition Explored by Molecular Dynamics Simulation. Surf. Coat. Technol..

[470] Illyaskutty N., Knoblauch J., Schwotzer M., Kohler H. (2015). Thermally Modulated Multi Sensor
Arrays of SnO2/Additive/Electrode
Combinations for Enhanced Gas Identification. Sens. Actuators, B.

[471] Dennler N., Drix D., Warner T. P. A., Rastogi S., Casa C. D., Ackels T., Schaefer A. T., van Schaik A., Schmuker M. (2024). High-Speed Odor Sensing Using Miniaturized Electronic
Nose. Sci. Adv..

[472] Ijaz U., Ali M., Ahmad I., Hamza S. A., Kim H. D. (2025). A Comprehensive Review of Electronic Nose Systems:
Design, Sensors, and Future Directions. Chem.
Eng. J..

[473] Chakraborty U., Kaushik A., Chaudhary G. R., Mishra Y. K. (2024). Emerging Nano-Enabled
Gas Sensor for Environmental
Monitoring–Perspectives and Open Challenges. Curr. Opin. Environ. Sci. Health.

[474] Hakeem Anwer A., Saadaoui M., Mohamed A. T., Ahmad N., Benamor A. (2024). State-of-the-Art Advances and Challenges
in Wearable
Gas Sensors for Emerging Applications: Innovations and Future Prospects. Chem. Eng. J..

[475] Jannat A., Talukder M. M. M., Li Z., Ou J. Z. (2025). Recent
Advances in Flexible and Wearable Gas Sensors Harnessing the Potential
of 2D Materials. Small Sci..

[476] Chowdhury M. A. Z., Oehlschlaeger M. A. (2025). Artificial
Intelligence in Gas Sensing:
A Review. ACS Sens..

[477] Barakeh Z. Al., Breuil P., Redon N., Pijolat C., Locoge N., Viricelle J. P. (2017). Development
of a Normalized Multi-Sensors
System for Low Cost on-Line Atmospheric Pollution Detection. Sens. Actuators, B.

[478] Güntner A. T., Pineau N. J., Mochalski P., Wiesenhofer H., Agapiou A., Mayhew C. A., Pratsinis S. E. (2018). Sniffing
Entrapped Humans with Sensor Arrays. Anal. Chem..

[479] Pineau N. J., Kompalla J. F., Güntner A. T., Pratsinis S. E. (2018). Orthogonal
Gas Sensor Arrays by Chemoresistive Material
Design. Microchim. Acta.

[480] Mei H., Peng J., Wang T., Zhou T., Zhao H., Zhang T., Yang Z. (2024). Overcoming
the Limits of Cross-Sensitivity:
Pattern Recognition Methods for Chemiresistive Gas Sensor Array. Nano-Micro Lett..

[481] van den Broek J., Weber I. C., Güntner A. T., Pratsinis S. E. (2021). Highly Selective Gas Sensing Enabled by Filters. Mater. Horiz..

[482] Maier I., Fieber M. (1988). Retention Characteristics of Volatile
Compounds on Tenax TA. J. High Resolut. Chromatogr..

[483] Rajendran A., Paredes G., Mazzotti M. (2009). Simulated Moving Bed
Chromatography for the Separation of Enantiomers. J. Chromatogr. A.

[484] van den Broek J., Abegg S., Pratsinis S. E., Güntner A. T. (2019). Highly Selective Detection of Methanol over Ethanol
by a Handheld Gas Sensor. Nat. Commun..

[485] Abegg S., Magro L., van den Broek J., Pratsinis S. E., Güntner A. T. (2020). A Pocket-Sized Device Enables Detection
of Methanol Adulteration in Alcoholic Beverages. Nat. Food.

[486] Jeong S. Y., Moon Y. K., Wang J., Lee J. H. (2023). Exclusive
Detection of Volatile Aromatic Hydrocarbons Using Bilayer Oxide Chemiresistors
with Catalytic Overlayers. Nat. Commun..

[487] Moon Y. K., Jeong S. Y., Kang Y. C., Lee J. H. (2019). Metal Oxide
Gas Sensors with Au Nanocluster Catalytic Overlayer: Toward Tuning
Gas Selectivity and Response Using a Novel Bilayer Sensor Design. ACS Appl. Mater. Interfaces.

[488] Pineau N. J., Magro L., van den
Broek J., Anderhub P., Güntner A. T., Pratsinis S. E. (2021). Spirit
Distillation: Monitoring Methanol Formation with a Hand-Held Device. ACS Food Sci. Technol..

[489] Güntner A. T., Magro L., van den
Broek J., Pratsinis S. E. (2021). Detecting Methanol in Hand Sanitizers. iScience.

[490] Güntner A. T., D’Andria M., van den Broek J. (2023). The Road to
Commercializing the Mobile Methanol Detector Alivion Spark M-20. Nat. Rev. Bioeng..

[491] van den Broek J., Keller S. D., Goodall I., Parish-Virtue K., Bauer-Christoph C., Fuchs J., Tsipi D., Güntner A. T., Blum T., Mathurin J. C., Steiger M. G., Shirvani R., Gössinger M., Graf M., Anderhub P., Z’graggen D., Hüsser C., Faigle B., Agapios A. (2024). Handheld Methanol
Detector
for Beverage Analysis: Interlaboratory Validation. Anal. Methods.

[492] van den Broek J., Klein Cerrejon D., Pratsinis S. E., Güntner A. T. (2020). Selective Formaldehyde Detection
at Ppb in Indoor Air
with a Portable Sensor. J. Hazard. Mater..

[493] Ren Q., Mo S., Peng R., Feng Z., Zhang M., Chen L., Fu M., Wu J., Ye D. (2018). Controllable
Synthesis of 3D Hierarchical Co3O4 Nanocatalysts with Various Morphologies
for the Catalytic Oxidation of Toluene. J. Mater.
Chem. A.

[494] Jeong H.-M., Jeong S.-Y., Kim J.-H., Kim B.-Y., Kim J.-S., Abdel-Hady F., Wazzan A. A., Al-Turaif H. A., Jang H. W., Lee J.-H. (2017). Gas Selectivity Control in Co_3_O_4_ Sensor via Concurrent Tuning of Gas Reforming
and Gas Filtering Using Nanoscale Hetero-Overlayer of Catalytic Oxides. ACS Appl. Mater. Interfaces.

